# Improving model predictions for RNA interference activities that use support vector machine regression by combining and filtering features

**DOI:** 10.1186/1471-2105-8-182

**Published:** 2007-06-06

**Authors:** Andrew S Peek

**Affiliations:** 1 Department of Bioinformatics, Integrated DNA Technologies, Inc., 1710 Commercial Park, Coralville, IA 52241, USA

## Abstract

**Background:**

RNA interference (RNAi) is a naturally occurring phenomenon that results in the suppression of a target RNA sequence utilizing a variety of possible methods and pathways. To dissect the factors that result in effective siRNA sequences a regression kernel Support Vector Machine (SVM) approach was used to quantitatively model RNA interference activities.

**Results:**

Eight overall feature mapping methods were compared in their abilities to build SVM regression models that predict published siRNA activities. The primary factors in predictive SVM models are position specific nucleotide compositions. The secondary factors are position independent sequence motifs (*N*-grams) and guide strand to passenger strand sequence thermodynamics. Finally, the factors that are least contributory but are still predictive of efficacy are measures of intramolecular guide strand secondary structure and target strand secondary structure. Of these, the site of the 5' most base of the guide strand is the most informative.

**Conclusion:**

The capacity of specific feature mapping methods and their ability to build predictive models of RNAi activity suggests a relative biological importance of these features. Some feature mapping methods are more informative in building predictive models and overall *t*-test filtering provides a method to remove some noisy features or make comparisons among datasets. Together, these features can yield predictive SVM regression models with increased predictive accuracy between predicted and observed activities both within datasets by cross validation, and between independently collected RNAi activity datasets. Feature filtering to remove features should be approached carefully in that it is possible to reduce feature set size without substantially reducing predictive models, but the features retained in the candidate models become increasingly distinct. Software to perform feature prediction and SVM training and testing on nucleic acid sequences can be found at the following site: .

## Background

RNA interference (RNAi) describes the property of short (21 to 23 base) RNA molecules, or short interfering RNA (siRNA), to associate with naturally occurring cellular machinery, the RNA-Induced Silencing Complex (RISC) and reduce the quantity of a second RNA molecule, or the target gene RNA [1,2]. In the relationship between the siRNA and the target RNA, the siRNA must be able to Watson-Crick base pair with some segment of the target RNA using standard base pairing rules. The RISC then catalytically cleaves the target RNA.

In addition to the RISC mediated silencing mechanism, the siRNA can reduce target gene levels utilizing two other methods. First, siRNA can inhibit transcription of the target gene's DNA [2-4]. Second, it can utilize a mechanism similar to an endogenous and highly conserved class of small RNAs known as microRNAs (miRNAs). MicroRNAs mediate the reduction of target gene protein level by repressing target RNA translation through imperfect base pairing to the target gene transcript [5]. All of these various methods and mechanisms result in target gene knockdown [6]. In addition to the epigenetic gene knockdown, siRNA sequences can cause sequence expulsion from the genome [7] and small dsRNAs are implicated in the induction of transcription [8].

SiRNA molecules are not all equally effective in their ability to knockdown target genes [9-14]. Some combination of the properties of the siRNA, the target RNA sequence and their interacting components are thought to account for the differential effectiveness. Furthermore, it is not known whether specific characteristics of an siRNA molecule contribute differently to the 3 gene knockdown mechanisms of RISC mediated, transcription inhibition and translation repression, since presumably each mechanism interacts with distinct subsets of cellular components and possibly different optimality criteria [15,16]. In addition to the mechanism of knockdown, there is also possible variation among transcripts [17], organisms, cell type, developmental time course, transfection methods [18] and environmental treatment in gene knockdown, and many of these properties are not accounted for in siRNA effectiveness. Although several rules describing properties of functional siRNA sequences have been proposed and proven to work with variable effectiveness, the fundamental questions of what properties comprise an effective siRNA for gene knockdown, by any mechanism, are unsettled. More realistic models will be needed for further dissecting siRNA mechanism or mechanisms [19]. Once appropriate experiments are derived for taking each of the complex series of variables into account, researchers will need to identify the critical components to model RNA interference activities and then use those models to develop reagents with the desired properties.

Several methods for identifying the properties of effective versus ineffective siRNA molecules from empirical data have included the following:

a. classification by statistical grouping [9-14,20,21]

b. classification and regression by neural networks [22-24]

c. classification by boosted genetic programming [25]

d. classification by decision trees [26,27]

e. classification and regression by support vector machines (SVMs) [25,28,29].

Many of the classification approaches have taken empirically derived continuously distributed data, and used it to map "effective" versus "ineffective" siRNA sequences and their associated properties by cutoffs and binning. A comparison of various algorithms in predicting siRNA efficacy by classification [30] suggests a large variance in performance. Furthermore, several features have been shown to associate with predictive models of activity including the following:

a. position specific base composition [11-14,20,29,30]

b. guide strand thermodynamics [9,10,24,25,29]

c. guide strand secondary structure [30,31]

d. structure features that discriminate microRNAs [32]

*e. N*-grams [25,28,29]

f. target strand secondary structure [21,24,33-40]

g. the energetics of multiple guide strand binding sites within the target [24].

Support Vector Algorithms or Support Vector Machines (SVMs) are a group of machine learning methods that build a maximum margin hyperplane through *n*-dimensional space to separate the *m *elements in a discrete classification problem [41]. The *n*-dimensional space is comprised of some set of factors that describe the *m *elements being classified. In addition to discrete classification, SVMs can also be used to build regression models in *n*-dimensional space. Generally this can be done by describing the regression as a set of 2*m *classification support vectors that separate the *m*-elements in the dataset. In fact, the single hyperplane SVM classification problem is a special case solution of the more general multi-hyperplane SVM regression problem [41]. Finally, SVM methods can extend beyond linear models to describe the maximum margin hyperplane(s) of the support vector solution space by non-linearly mapping the initial vector into higher dimensional feature space [42].

SVM regression kernel methods produce varied results depending on the application, and kernel performance needs to be determined empirically [43]. Also, feature-mapping methods have an effect on SVM performance [42]. Given the observation that SVM kernel methods are effective at defining maximum margin hyperplanes and the knowledge that results can depend on feature mapping to vector space, this study investigates several feature mapping methods and examines their utility in creating predictive regression models for siRNA activity.

Given that several types of sequence based features can be used to build predictive models of RNAi, one of the main intentions of this study is to first ask what features individually correlate with RNAi efficacy to help identify additional siRNA properties that may have structural or functional importance previously not seen. A second intention is to ask if there is a consensus as to the feature mapping methods that can be used either alone or together and do they contribute to developing models generally predictive of activity on data not seen during model training. Furthermore, do feature selection methods, such as feature filtering, on large feature sets actually improves predictive models or if feature subsets are found in common. Two datasets are used in the present study. The first is a set of 2431 siRNA sequences of 21 nucleotides in length from [23], specifically from the corrigendum [44], referred to as dataset_2431_. The second is a compiled set of 579 siRNA sequences of 19 nucleotides in length from [25] referred to as dataset_579_.

## Methods

### RNA interference and target sequence data

Dataset_2431 _was from [23], the 21-mer sequence and activity data used was from the corrigendum [44]. Dataset_579 _was from the compiled 581 19-mer sequences and activities dataset used by [25], with the exception of five sequences that did not precisely correspond to their target gene DNA sequence. Of these five sequences, two were discarded due to ambiguity of matching to their target and three were changed at one or two positions to correctly correspond to the target mRNA sequence. The target mRNA sequences were either from [23] or downloaded from the NCBI [45].

### data mapping methods for SVM

The following eight general approaches, in Roman numerals, were used to map a sequence to a vector space, to result in 14 methods, labeled in Arabic numerals:

I. position specific base composition (method 1)

II. thermodynamics (method 2)

III. entropy (method 3)

IV. guide strand structure (method 4)

V. guide strand structure features (method 5)

VI. *N*-grams (methods 6–11)

a. *N*-grams *N *= 2 (method 6)

b. *N*-grams *N *= 3 (method 7)

c. *N*-grams *N *= 4 (method 8)

d. *N*-grams *N *= 5 (method 9)

e. *N*-grams *N *= 6 (method 10)

f. *N*-grams *N *= 2 through 5 (method 11)

VII. target strand structure (methods 12–13)

a. target strand structure – nondirectional (method 12)

b. target strand structure – directional (method 13)

VIII. target imprecise thermodynamics (method 14)

### method 1: position specific base composition

Each position in the siRNA sequence was mapped to four dimensions in vector space, where each dimension corresponded to one of the bases in the DNA alphabet. The relationship between the length of the sequence (*L*) and the number of dimensions of vector space (*M*) was then *M *= *S *x*L*, where *S *is the size of the alphabet, in this case 4 for nucleic acids. For example, using the coding system between DNA base and vector results in the following mapping:

A = < 1,0,0,0 >

C = < 0,1,0,0 >

G = < 0,0,1,0 >

U/T = < 0,0,0,1 >

### method 2: thermodynamics

The thermodynamics mapping method has 23 dimensions, with 20 of the dimensions corresponding to the Gibbs free energy stabilities of the nucleotide pairs of the 21-nucleotide RNA molecule. An additional two dimensions were for the stability energetics of the terminal 5' and 3' ends, encompassing 4 nucleotide sites. The final dimension is the Gibbs free energy stability of the entire sequence. The nearest neighbor model predicted Gibbs free energies with the RNA parameters of Xia [46].

### method 3: Shannon entropy

The Shannon entropy mapping method is similar in dimensionality and implementation to the thermodynamics method, but the 23 dimensions of the 20 nucleotide pairs, the 5' and 3' terminal ends and the final dimension of the entire 23 nucleotide sequence were populated with Shannon's measure of bitwise information content [47] by formula (1).

H(X)=−∑i=1lp(xi)log⁡2(p(xi))
 MathType@MTEF@5@5@+=feaafiart1ev1aaatCvAUfKttLearuWrP9MDH5MBPbIqV92AaeXatLxBI9gBaebbnrfifHhDYfgasaacH8akY=wiFfYdH8Gipec8Eeeu0xXdbba9frFj0=OqFfea0dXdd9vqai=hGuQ8kuc9pgc9s8qqaq=dirpe0xb9q8qiLsFr0=vr0=vr0dc8meaabaqaciaacaGaaeqabaqabeGadaaakeaacqWGibascqGGOaakcqWGybawcqGGPaqkcqGH9aqpcqGHsisldaaeWbqaaiabdchaWjabcIcaOiabdIha4naaBaaaleaacqWGPbqAaeqaaOGaeiykaKIagiiBaWMaei4Ba8Maei4zaC2aaSbaaSqaaiabikdaYaqabaGccqGGOaakcqWGWbaCcqGGOaakcqWG4baEdaWgaaWcbaGaemyAaKgabeaakiabcMcaPiabcMcaPaWcbaGaemyAaKMaeyypa0JaeGymaedabaGaemiBaWganiabggHiLdaaaa@4CD9@

Where *l *is the length of the sequence, *p*(*x*_*i*_) is the frequency of the character at position *i*.

### method 4: guide strand secondary structure

Nucleic acid secondary structure describes the ability of a single molecule of nucleic acid sequence to form one or more intramolecular bonds, thereby stabilizing some sequence segments as double stranded. siRNA sequence secondary structures were predicted with the RNAfold as implemented in the Vienna package [48]. Energetics were predicted by partition function and by minimal free energy algorithms for evaluation purposes. Partition function energetics produced models with higher predictive accuracy and was used in this study. First, a 21-length feature vector was produced with one dimension for each base position in the siRNA sequence corresponding to whether the position was involved in an intramolecular secondary structure. Second, a single dimension was added corresponding to the overall intramolecular stability as measured by the Gibbs free energy of folding. Finally, two additional dimensions were numerical counts of the number of bases in the 7 most 5' and 7 most 3' bases of siRNA sequence involved in a predicted secondary structure [31].

### method 5: guide strand secondary structure features

The guide strand secondary structure features mapping method is an implementation of the sequence feature method described by Xue *et al*. [32] for discriminating real and pseudo miRNAs. Briefly, a 32-length feature vector is comprised of the occurrence frequencies of three nucleotide sequence-structure features. The middle base of the 3 base triplet has one of 4 possibilities (A, C, G or T/U) and each position could be in either a bonded or non-bonded state resulting in a 32 (4 × 2^3^) dimensional feature space. The nomenclature used is the base at the middle position and then 3 binary symbols. For example, 'U000' indicates the middle position is 'U' and this 3 base triplet is not within a secondary structure, whereas 'C111' indicates the middle base position is a 'C' and this triple is completely paired within a structure. See Xue *et al*. [32] for complete details.

### methods 6–11: *N*-gram

The *N*-Gram approach mapped the presence or absence of each possible sub word of a given length and character composition from the original siRNA sequence[25]. For example, there are 4^2 ^= 16 possible 2-grams from the 4 base DNA alphabet, (generally, *A*^*N *^where A is the number of characters in the alphabet and *N *is the length of the word). The 16 length 2-gram vector for the DNA 'ACGT' alphabet would then be:

< AA, AC, AG, AT, CA, CC, CG, CT, GA, GC, GG, GT, TA, TC, TG, TT >

and mapping the previous example sequence of "ATGCATG" onto this vector space by presence or absence would yield:

< 0, 0, 0, 1, 1, 0, 0, 0, 0, 1, 0, 0, 0, 0, 1, 0 >

The *N*-Gram method is therefore position independent, and vector space can be adjusted to account for frequency and position in addition to simply presence or absence.

### methods 12–13: target strand secondary structure

The predicted secondary structures for the mRNA target sequences were determined in the same identical manner as the siRNA guide sequences. Regions of structure prediction were limited to the guide strand binding region plus 100 bases up and down stream. However the guide strand binding region was used to map structure to vector space. In the case of direction independent structure, this resulted in a total of 22 dimensions: 21 dimensions with one for each nucleotide position plus an additional dimension as the Gibbs free energy of structural stability. For directional binding in the target structure, the dimensions were 42 plus the Gibbs free energy, totaling 43.

### method 14: target strand multiple binding patches

The guide strand of the siRNA sequence could imperfectly pair with multiple regions of the target strand. The 22 most stable imperfect sites of guide strand to mRNA pairing were predicted by RNA thermodynamics [46] and their thermodynamic stabilities populated the dimensions of the feature vector.

### SVM regression kernel methods

Four regression kernel functions were tested:

1. Linear kernel

2. Polynomial kernel

3. Radial Basis Function (RBF) kernel – This is a similar in implementation to the radial basis function neural network.

4. Sigmoid kernel – This is similar to another type of neural network, a multilayer perceptron with no hidden layers.

SVM kernels were implemented with the libsvm library [49].

SV regression was used rather than SV classification, since the activity data were continuously distributed on the interval [0, 1]. Here we tested classification models to predict RNAi activities, but choosing arbitrary division points in the outcome classes resulted in highly variable model performance. This observation suggests that data categorization has a sufficient impact in model building and that the optimization of data categorization is important.

### *N*-fold cross validation within a dataset

Cross validation (CV) was performed by the method of dividing the original data into *N *equally sized (or as nearly as possible) partitions and trained on (*N*-1) partitions and tested on the *N*^th ^partition. This was performed for all *N *partitions and the Pearson correlation coefficient (*R*) and mean squared error (*MSE*) between predicted and observed on the testing partition was averaged for all *N *tests. Specifically, 10-fold cross validation on dataset_2431 _divided the dataset into 10 datasets of size 243. A model was then trained on a dataset of size 2187, and then tested on the remaining data of 243. This procedure was repeated 9 more times on the remaining partitions. Values of *R *and *MSE *are comparable within tables from cross validation in that the same pseudo-random number seed was used to produce the dataset divisions. Cross validations involving feature selection were performed by using the feature selection method only on the training set and applying this feature subset to the training set. Performing feature selection within the cross validation reduces the bias in CV model estimates, but can result in different feature sets being used among the partitions of cross validation. The average number of features used among partitions, and the similarities among the CV feature subsets is reported where appropriate.

### individual feature correlation to RNAi activity and feature filtering

Individual features were tested for their significance of correlation to activity by correlation and the *t*-test of significance, calculated by formula (2).

t=|R×((o−2(1−R2)))|

where

*R *= Pearson correlation coefficient

*o *= number of observations

*R*^2 ^= Pearson correlation coefficient squared (coefficient of determination)

Feature filtering used only the training portion of the dataset to perform feature subset selection, along with appropriate calculation metrics on the training dataset, and this feature subset was then applied to the naive testing dataset. Evaluating feature selection within cross validation reduces bias in assessing model performance metrics when the same dataset is not in both model training and then model testing. By contrast, when the entire dataset is used for both training and testing, the results are optimistically biased due to model over fitting. When the training and testing are performed alternatively between dataset_2431 _and dataset_579_, the results are likely to be pessimistically biased, principally due to the dissimilarities between the datasets.

The feature selection method of Correlation based Feature Selection (CFS) [50] was used to select feature subsets with presumed high effectiveness. CFS is a maximum-relevance minimum-redundancy method that greedily adds features to a feature subset by maximizing a scoring metric. CFS used equation (3) to maximize *G*_*s *_in selecting features for the subset.

Gs=krcik+k(k−1)rii
 MathType@MTEF@5@5@+=feaafiart1ev1aaatCvAUfKttLearuWrP9MDH5MBPbIqV92AaeXatLxBI9gBaebbnrfifHhDYfgasaacH8akY=wiFfYdH8Gipec8Eeeu0xXdbba9frFj0=OqFfea0dXdd9vqai=hGuQ8kuc9pgc9s8qqaq=dirpe0xb9q8qiLsFr0=vr0=vr0dc8meaabaqaciaacaGaaeqabaqabeGadaaakeaacqWGhbWrdaWgaaWcbaGaem4Camhabeaakiabg2da9maaliaabaGaem4AaSMaemOCai3aaSbaaSqaaiabdogaJjabdMgaPbqabaaakeaadaGcaaqaaiabdUgaRjabgUcaRiabdUgaRjabcIcaOiabdUgaRjabgkHiTiabigdaXiabcMcaPiabdkhaYnaaBaaaleaacqWGPbqAcqWGPbqAaeqaaaqabaaaaaaa@4319@

where *k *is the number of features in the subset, *r*_*ci *_is the mean correlation of the feature to the outcome and *r*_*ii *_is the mean feature intercorrelation or feature to feature cross correlation.

Multicollinearity exists within and between some of the feature mapping methods. For example, the base composition at positions 1 and 2 (method 1) correspond to the thermodynamics measurement for this area (method 2) and these share significant cross correlations.

### software architecture

A group of C++ classes are made available to the research community that performs the following functions:

1. SVM model construction, given a feature set and RNAi sequence dataset

2. Perform *N*-fold cross validation given a model, feature set and RNAi sequence dataset

3. Predict RNAi activities given an SVM model, feature set and a candidate RNAi sequence set

4. Predict siRNA sequences given a feature set, candidate gene sequence and a SVM model,

5. Predict various types of feature filters, feature comparisons as well as feature cross-correlation

The most recent library classes and associated main functions can be downloaded [51].

Software was developed with C++ under Linux kernel 2.6.9-5, with the gcc compiler 3.4.3. The classes for manipulating and modeling siRNA sequences and their activities compile without warnings with the -Wall -ansi -pedantic-errors compilation flags, including wrapper classes for libsvm-2.71 and libRNAfold-2.4 libraries. Additional platforms and compilers have not been systematically tested, but the package is distributed with the GNU autotools and should compile on supported architectures. Further development of additional functionality for this library is intended and the resulting code will also be released. Areas of development include interfaces to other machine learning techniques including ANN's, additional feature mapping methods and implementing wrapper methods for model construction and optimization. Contact the author if you intend to develop functionality, primarily to ensure a minimal duplication of effort, if the method has already been constructed and not released.

## Results

The results section is divided into three major sections with the following structure. The first investigates individual feature correlation with RNAi activity involving only dataset_2431_. This section specifically examines the methods of site-specific base composition (method 1), guide strand thermodynamics (method 2), guide strand entropy (method 3), guide strand secondary structure (method 4), guide strand secondary structure feature (method 5), target sequence secondary structure (methods 12 and 13) and finally *N*-Grams (methods 6 to 11).

The second section investigates these single feature mapping methods and their abilities to train and test SVM models on two datasets: dataset_2431 _and dataset_579_. The second section also introduces feature filtering by *t*-test, features removed by increasing stringency of *t*-test of individual feature to RNAi activity.

The final section investigates the effectiveness of both combining individual feature mapping methods and feature filtering by Correlation based Feature Selection (CFS) to produce feature subsets in the training and testing of SVM models on dataset_2431 _and dataset_579_. Also feature subset comparisons are made, investigating the commonality between predictive feature subsets derived from either within the same dataset between cross validations or between different datasets.

### I a. site specific base composition

**Figure 1:**
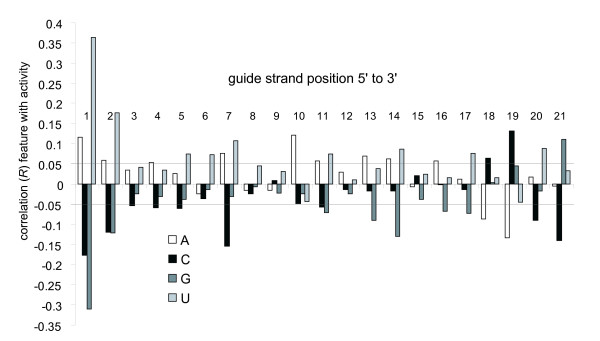
**Guide strand position specific base composition correlation coefficients with RNA interference activities**. Positive correlations can be interpreted as the presence of the nucleotide at the position leads to greater RNAi activity, while negative correlations are the presence of the nucleotide resulting in a decreased RNAi activity. Position 1 is the 5' most position of the guide strand.

The correlation of position specific base composition to RNAi activity was calculated for each of the 84 features in the position specific base composition vector. Overall, there are 45 features that have a correlation with RNAi activity with a *t*-test value of 2.0 (*P *< 0.05) or greater (Figure 1, horizontal lines at correlation *R *= +/-0.05 have *t*-test values of ~2.4 and simply provide visual landmarks). Statistical tests have not been corrected for multiple comparisons and there are several kinds of non-independence within the data, features, models and tests presented. Many of these bases and positions are consistent with previous observations of site-specific base composition (see Suppl1_comparison_position_specific_base_composition.xls), but several have not been previously identified as statistically significant. Previous analyses even from the same dataset yield inconsistencies in features found to be or not be significant.

Briefly, the method for identifying position specific biases in base composition from this data previously used the 200 most potent and 200 least potent siRNA sequences rather than the entire dataset [23], so differences are not unexpected. For example, sites that have not previously been shown as significantly associated with RNAi efficacy: C3 (namely a "C" base at the 3^rd ^position in the guide strand, starting from the 5' end of the guide strand), C5, C10, G11, G17 are overly associated with lower potency and U6, U8, A16, T20 are overly associated with higher potency, numbering from the 5' end of the guide strand. In general, from the 45 features that have values of *t *greater than 2.0, the features are relatively evenly distributed across bases: 11 A's, 12 C's, 9 G's and 13 U/T's, but not in their association with lower potency: 2 A's, 10 C's, 7 G's, 2 U/T's versus higher potency: 9 A's, 2 C's, 2'G's, 11 U/T's, and their distribution across positions are irregular (Figure 1).

In addition to the guide strand of the siRNA, site-specific base composition biases might exist in the target mRNA as well. Investigating this possibility in the target mRNA surrounding the guide strand-binding region resulted in 3 overall patterns. First, the guide strand binding area on the target strand has the largest magnitude of site-specific base composition biases, when compared to the surrounding 100 bases (Supplementary figure 1). Second, the magnitude of the positive correlation drops with distance from the guide strand whereas the magnitude of the negative correlation appears reasonably constant. Third, the overwhelming trend for positive correlations with activity relates to the bases A and T/U. The trend for negative correlations with activity relates to the bases G and C (Supplementary Figure 2). Despite these suggestive patterns, no dominant features of site-specific base composition were obvious outside of the guide strand binding area, and further study of site-specific base composition was limited to the guide strand region.

**Figure 2:**
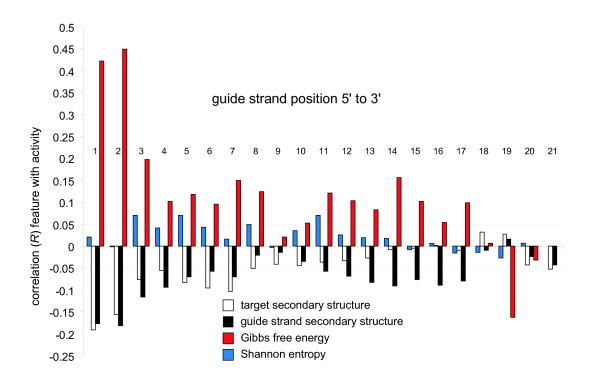
**Guide strand secondary structure, thermodynamics, entropy and target secondary structure position specific correlation coefficients with RNA interference activities**. Thermodynamics and entropy correlation measures comprise a 2 nucleotide sliding window. Secondary structures can be interpreted as the relative contribution of this position being within a secondary structure and that contribution leads to RNAi activity. Position 1 is the 5' most position of the guide strand.

### I b. guide strand thermodynamics, entropy, secondary structure

Guide strand thermodynamics (*R *= 0.283), guide strand sequence entropy (*R *= 0.074), guide strand secondary structure stability (*R *= 0.227) and overall target strand secondary structure stability (*R *= 0.248) all have correlations with RNAi activity that have high *t*-values. In addition, these features have position specific distributions from within the guide strand (Figure 2). Correlations between activity and guide strand thermodynamics, guide strand secondary structure and target secondary structure have been shown before and we see overall correlations between these features and RNAi activity as well. Also, position dependence of guide strand thermodynamics has also been shown previously and this is seen in the present data as well (Figure 2). Additionally, there is a general positive association between the entire guide sequence's information content (Shannon entropy) and activity, where guide sequences with higher information content (lower repeat structure, a more even distribution of bases, etc.) have higher potency. There is also a weak indication that this pattern is seen in positions 3 through 9 of the guide strand (Figure 2).

### I c. sequence structure features

Recently, a sequence structure mapping method was proposed that allowed the discrimination of real versus pseudo microRNAs [32] by combining sequence and secondary structure. Applying this method on the guide strand sequence, several sequence-structure features were observed that had positive or negative correlations with activity. Using the nomenclature described in the methods section, features such as U/T000 (*R *= 0.152) and A110 (*R *= 0.099) had a positive correlation as well as sequence-structure features that had a negative correlation C111 (*R *= -0.160) and G111 (*R *= -0.129). Generally, open structures are preferred to bonded structures and the bases A and U/T are preferred to C and G (see Suppl2_all_features_corr_descr_tval.txt for a list of individual feature to activity correlates for thermodynamics, structure, entropy, etc.).

### I d. target secondary structure

**Figure 3:**
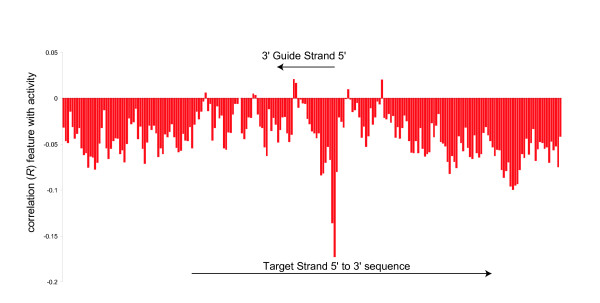
**Target secondary structure position specific correlation coefficients to RNA interference activities**. Individual positions within an intramolecular base pairing tend to have an overall negative correlation with RNAi activity.

Investigating the target strand secondary structure more fully, the target strand secondary structure was predicted and the positions surrounding the guide strand binding area were interrogated to see whether they form pairs in an intramolecular target strand structure. Intramolecular interactions that were limited to 100 nucleotide sites upstream and downstream of the guide strand binding area were used in the presented data. Folding areas of 20, 50, 75, 80, 125, 150 and the entire target strand were investigated and were, on the whole consistent. However, 100 sites resulted in the highest correlation between target strand structure stability and RNAi activity, similar to the observations of [37]. Graphing the correlations between each position in the target strand that is within an intramolecular structure and the RNAi activity resulted in two overall patterns (Figure 3). First, there is an overall negative correlation between any site within the local target area being paired and RNAi activity (with a few potentially positively correlating areas or anomalous regions near or within the guide strand binding area) that is consistent with the observation that there is a correlation between target strand structure stability and activity. Second, the most dominant negatively correlative position that results in lower potency siRNA sequences occurs where the 5' most site of the guide strand would pair to the target strand within an intramolecular Watson-Crick pair.

**Figure 4:**
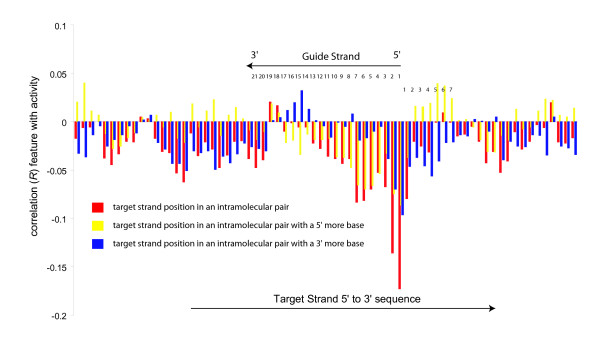
**Target secondary structure position specific correlation coefficients with directionality of base pairing to RNA interference activities**. Correlations of the site pairing with 5' more site are in yellow and pairing with a 3' more site are in blue.

Target secondary structure was further investigated by asking whether there are any structural patterns in the overall orientation of the Watson-Crick pairing within the immediate region of the guide strand. Intramolecular bonds were categorized into those occurring to a base more 5' on the target strand and those occurring to a base more 3' of itself (respectively yellow and blue in Figure 4) on the target strand. There are two patterns that emerge from this analysis. The first pattern is the highly deleterious position where the guide strand's 5' most base would pair. It is fairly equally comprised of structures that involve sites that are both 5' and 3' of itself, suggesting guide strand access is not asymmetric. Second, there appears to be a weak symmetry of sites immediate to the 3' of the guide strand binding area, (positions 2 through 7 on the area 3' of the guide strand binding region, Figure 4) on the target strand to be positively correlated with activity if bonding with a 5' more site and negatively correlated with activity if bonding with a 3' more base. This weak symmetry is reflected within the guide strand binding area (positions 13 though 17 in the guide strand, Figure 4) where these positions are weakly positively correlated with activity if bonding with a 3' more site and negatively correlated with activity if bonding to a 5' more base. The overall suggestion might be that structures that hold the 5' most site of the guide strand's pair in a target secondary structure are deleterious whereas nearby target secondary structure stems that hold this position in an unstructured loop are more (weakly) positive for RNAi activity. Since this is an analysis that comprises several thousand guide strand regions, it is necessarily a population average. Therefore, individual cases where this is not observed would not be surprising.

### I e. *N*-grams

Sequence motifs, or *N*-grams, simply a subsequence of *N *items from a given sequence, were then investigated for motif specific correlation with RNAi activity (see supplementary table 3 for complete table of feature *N*-gram correlations with activity). Overall, 10 of the 16 possible 2-grams had *t*-values greater than 2.0, 6 with positive correlations tending to be A and U/T rich ("AA" *R *= 0.090, "AT" *R *= 0.118, "TA" *R *= 0.174, "TC" *R *= 0.047, "TG" *R *= 0.053 and "TT" *R *= 0.153) and 4 with negative being the four possible combinations of both C and G base ("CC" *R *= -0.088, "CG" *R *= -0.089, "GC", *R *= -0.120, "GG" *R *= -0.114). This overall pattern holds true for the 3 through 6 length *N*-grams with a general preference for A and U/T and aversion for C and G. Higher order patterns are seen in the preference or aversion to specific longer motifs as well. For example, there are 64 possible guide strand 3-grams and 39 of these 64 have *t*-values greater than 2.0. Furthermore, there are 114 of the 256 4-Grams with *t*-values for their correlations greater than 2.0. One striking observation is that overall for 3-grams, their individual 3-nucleotide motif associations with RNAi activity negatively correlate with their corresponding codon usage frequency (*R *= -0.221), reverse and complementing the guide strand 3-gram into the target strand codon sequence. Also, the magnitude of deviation for each 3-gram, as measured by *t*, negatively correlates with both the codon usage frequency (*R *= -0.127) and with synonymous codon usage frequency (*R *= -0.156).

**Table 3:** Guide strand position specific base composition (Method 1) for training RBF-epsilon regression SVM model

		train_2431_		train_2431_		train_2431_	
		test_2431_		test_579_		test_2431 _10 × cross val	
				
*t-*test	FN_2431_	*R*	*MSE*	*R*	*MSE*	*R*	*MSE*
0	84(84)	**0.784**	0.016	**0.509**	0.095	0.711	0.026
1	65(64.2)	0.774	0.016	0.500	0.098	**0.712**	0.025
2	45(43.9)	0.742	0.018	0.494	0.100	0.687	0.027
3	32(30.3)	0.706	0.020	0.478	0.103	0.675	0.027
4	20(18.9)	0.658	0.023	0.460	0.103	0.658	0.028
5	15(14.7)	0.627	0.024	0.437	0.101	0.648	0.029
6	11(8.3)	0.599	0.025	0.428	0.105	0.587	0.031
7	5(5)	0.489	0.030	0.340	0.104	0.532	0.034
8	4(3.7)	0.473	0.031	0.337	0.104	0.504	0.035
9	2(2)	0.407	0.033	0.256	0.108	0.454	0.037

Models trained on dataset_2431 _and testing performed with dataset_2431_, dataset_579 _and 10 × cross validation on dataset_2431_, features removed by increasing stringency of *t*-test of individual feature to activity from dataset_2431_.

Feature numbers in parentheses are the average number of features in cross validations.

Entries in **bold **are column maximums and are simply visual landmarks.

The observation that there may be some association between 3 nucleotide motifs associating with RNAi activity and codon usage preferences may be due to several non-mutually exclusive relationships. One possible relationship is the mutual lack of preference for specific dinucleotide pairs and higher order *N*-grams, which are clearly comprised of lower order *N*-grams and the relationships seen within the 3-grams and their underlying 2-grams. It is well known that there is to be an overall under abundance of the dinucleotide pair "CG" in most eukaryotic genomes [52] as well as a reduced preference for codons with the dinucleotide "CG" when compared with the dinucleotide "GC". However, concerning those specific dinucleotides within RNAi guide strands, the occurrence of the dinucleotide "CG" in the guide strand is significantly negatively correlated with activity (*R *= -0.089, *t *= 4.451) as is the dinucleotide "GC" (*R *= -0.120, *t *= 5.993). Furthermore, neither the guide strands nor the target sequences appear to have any specific over or under abundance, beyond what has been previously observed, of the "CG" dinucleotide.

To further investigate dinucleotide composition, there are 256 4-grams. Each 4-gram occurs in the present RNAi sequence dataset and either correlates positively or negatively with RNAi activity. A 2 × 2 contingency analysis was performed looking for an association between a 4-gram's positive or negative correlation with activity and the presence or absence of a specific dinucleotide pair. The results for negatively contributing dinucleotides were similar to the 2-gram correlations in that the dinucleotides "CC" *G *= 15.2, "CG" *G *= 21.2, "GC" *G *= 18.25 and "GG" *G *= 24.9 had significant, *G*-test (*G *> 10.0, *P *< 0.05), overabundance in negative correlated 4-grams when compared to 4-grams that did not contain those dinucleotides. By contrast, with the negative associating 2-grams, only three positively contributing dinucleotides, "AT" *G *= 10.9, "TA" *G *= 22.6 and "TT" *G *= 12.4, showed a significant *G*-test overabundance in positively associated 4-grams when compared to 4-grams that do not contain those dinucleotides.

Again, to more closely investigate dinucleotides in 4-grams with the intention of trying to reduce base composition biases, only those 4-grams with equal base composition were selected. There are 24 4-grams that each contains one of each of the four bases. Of those 24 4-grams, 6 have negative correlations with activity and the remaining 18 have positive correlations with RNAi activity. Performing a 2 × 2 contingency test on the presence versus absence of each of 12 dinucleotides (excluding the 4 homo-dinucleotide pairs: "AA", "CC", "GG" and "TT") for positive versus negative correlation yields a significant interaction (*P *= 0.017, Fischer's Exact Test, *FET*) only for the "CG" dinucleotide. This occurs in the "CG" dinucleotide, where 2 "CG" containing 4-grams positively correlate, 4 "CG" containing 4-grams negatively correlate, while 16 "CG" missing 4-grams positively correlate and the remaining 2 "CG" missing 4-grams negatively correlate with RNAi activity. The remaining hetero-dinucleotide pairs, including "GC" (*P *= 0.137, *FET*), do not suggest significant interactions between dinucleotide presence/absence and positive/negative correlation with RNAi activity in 4-grams with equal base composition. These observations may indicate some differential effect of nucleotide sequences on RNAi activity. The intent of these preliminary *N*-grams analyses is not necessarily to be complete, but to simply provide an initial description of some of the complexity seen in the present data as well as some possible explanatory patterns.

### II a. building predictive SVM models with features correlative with RNAi activity

One aspect of finding features that are correlative with an outcome, in this case RNAi activity, is to better understand the mechanisms that are important in the system. A second aspect of feature finding is determining how well these features, alone or together, are able to model the phenomenon under study and additionally, to determine specifically what features are able to predict outcomes that were not seen during model building.

In our research, we used the 14 feature mapping methods listed below:

1. position specific base composition

2. thermodynamics

3. entropy

4. guide strand structure

5. guide strand structure features

6. *N*-grams *N *= 2

7. *N*-grams *N *= 3

8. *N*-grams *N *= 4

9. *N*-grams *N *= 5

10. *N*-grams *N *= 6,

11. *N*-grams *N *= 2–5

12. target strand structure non-directional pairing

13. target strand structure directional pairing

14. multiple guide strand binding patch energetics on the target strand

We compared these 14 features in their abilities to yield predictive models by Radial Basis Function (RBF) Support Vector Machine (SVM), Table 1. Additional SVM kernels were investigated, but overall the Polynomial and RBF kernels performed slightly better than the Linear and Sigmoid on these data. Briefly, examination of the position specific base composition mapping (method 1) across kernels by 10-fold cross validation within dataset_2431 _resulted in the following kernel performance metrics, implementing a course grid search for kernel parameters:

**Table 1:** Feature mapping methods performance in RBF-epsilon regression SVM model training and testing within dataset_2431_

				train_2431_	
				test_2431 _10 × cross validation	
				
Feature mapping method	FN_2431_	*R*	*MSE*	*R*	*MSE*
1-Position specific base	84	0.784	0.016	0.711	0.026
2-Thermodynamics	23	0.915	0.007	0.640	0.029
3-Entropy	23	0.730	0.021	0.094	0.046
4-Guide strand structure	24	0.430	0.033	0.293	0.041
5-Guide strand features	32	0.266	0.037	0.243	0.042
6-*N*-Grams *N *= 2	16	0.408	0.033	0.291	0.041
7-*N*-Grams *N *= 3	64	0.656	0.024	0.435	0.037
8-*N*-Grams *N *= 4	256	0.590	0.027	0.532	0.034
9-*N*-Grams *N *= 5	1024*	0.590	0.029	0.487	0.036
10-*N*-Grams *N *= 6	4096*	0.621	0.036	0.439	0.036
11-*N*-Grams *N *= 2–5	1360*	0.614	0.026	0.559	0.033
12-Target strand structure-nondirectional	22	0.646	0.024	0.257	0.045
13-Target strand structure-directional	43	0.607	0.025	0.277	0.042
14-Target imprecise thermo	22	0.932	0.007	0.272	0.045

FN_dataset _= Feature Number count from dataset, *R *= Pearson correlation coefficient, *MSE *= mean squared error * Theoretical, but five 5-grams and several 6-grams are absent in the present dataset, reducing the effective feature set size.

Linear *R *= 0.698 *MSE *= 0.026 (epsilon = 0.0081)

Polynomial *R *= 0.708 *MSE *= 0.026 (degree = 2, epsilon = 0.0071)

RBF *R *= 0.710 *MSE *= 0.026 (C = 0, gamma = 0, epsilon = 0.0091)

Sigmoid *R *= 0.695 *MSE *= 0.026 (gamma = 0, epsilon = 0.0041)

Additionally, RBF kernels overall resulted in the largest correlation values between predicted and observed activities in cross validation across kernel methods. RBF kernels used parameters of gamma = 0.01 and p = 0.1, both empirically derived by cross validation as optimal from the method 1 and dataset_2431_. Deviations in these kernel parameter settings across additional methods and datasets had minimal influence on the resulting models, but parameter optimization across feature mapping method, feature subsets and dataset was not investigated in detail.

Across feature mapping methods, overall, the number of features in the model varied from 16 to over 4000, as did the correlation coefficient (*R*) from 0.2 to 0.9, Table 1. However, the entire dataset being used to both train and test the model is not a realistic measure of how well the model might perform on data that was not seen during model building. Therefore, 10-fold cross validation was performed. Additionally, over fitting a model is a concern, particularly when the size of the feature space grows to exceed the size of the dataset. Evidence of this is seen in some of the longer *N*-grams, specifically where *N*= 6, feature set size exceeds 4000 and the dataset size is 2431. During 10-fold cross validation, *R*'s were, as expected, lower than their corresponding complete dataset *R*'s. All mapping methods result in significantly (*P *< 0.05, H_O_: *R *= 0) positively predictive SVM models, on data not seen during model training within dataset_2431_, ranging from *R *= 0.094 to *R *= 0.711.

Comparisons among feature mapping methods suggested that all feature mapping methods provided some degree of predictive utility by cross-validation (Table 1). Additionally, as a compromise between predictive utility and feature set size, the *N*-gram method was limited to *N *= 2 through 5. Method 11-*N*-gram *N *= 2–5 results in greater predictive accuracy than individual *N*-grams where *N *= 2, 3, 4 and 5 separately and results in a moderate feature set size. Finally, comparisons between target secondary structure mapping methods that incorporate directionality of base pairing (method 13) performed better in cross-validation than not incorporating directionality (method 12).

Eight feature mapping methods (1, 2, 3, 4, 5, 11, 13 and 14) were investigated by training and alternatively testing SVM models on two datasets: dataset_2431 _and dataset_579_, Table 2. Five methods, position specific base composition, thermodynamics, guide strand structure, guide strand features and *N*-Grams *N *= 2–5 (methods 1, 2, 4, 5, and 11), resulted in positive predictive models both within and between datasets. Namely, each of these five methods resulted in a significantly positive predictive models by cross validation and when trained on one dataset and tested on the other dataset. Both target strand structure and off target thermo (methods 13 and 14) are consistently predictive within either dataset_2431 _or dataset_579 _by cross-validation but not alternatively between datasets. Finally, the feature of guide strand entropy (method 3) yields positively predictive models when training on dataset_2431_, but not when training on dataset_579_, Table 2.

**Table 2:** Feature mapping method performance in RBF-epsilon regression SVM modeling, alternatively training and testing between dataset_2431 _and datatset_579 _and 10 × cross validation within dataset_2431 _or datatset_579_

		train_2431_				train_579_			
			
		test_579_		test_2431 _10 × cross validation		test_2431_		test_579 _10 × cross validation	
					
Method	FN_2431_ FN_579_	*R*	*MSE*	*R*	*MSE*	*R*	*MSE*	*R*	*MSE*
1-	84	0.510	0.095	0.711	0.026	0.485	0.054	0.562	0.079
2-	23	0.379	0.105	0.640	0.029	0.367	0.069	0.500	0.087
3-	23	0.130	0.115	0.094	0.046	0.017^†^	0.138	0.026^†^	0.118
4-	24	0.202	0.115	0.293	0.041	0.214	0.073	0.214	0.041
5-	32	0.214	0.112	0.243	0.042	0.164	0.046	0.194	0.107
11-	1360	0.247	0.109	0.559	0.033	0.192	0.055	0.469	0.088
13-	43	0.045^†^	0.111	0.277	0.042	0.071	0.104	0.262	0.105
14-	22	0.022^†^	0.107	0.272	0.045	0.020^†^	0.067	0.182	0.118

Method numbers are from Table 1.

FN_dataset _= Feature Number count from dataset

*R *= Pearson correlation coefficient, values with a ^† ^are not able to reject the *H*_O_: *R *= 0.

*MSE *= mean squared error

### II b. feature filtering on individual feature mapping methods

Methods 1, 2, 4, 5 and 11 resulted in positive predictive models both within datasets by cross validation as well as between datasets. Individually, these methods were investigated to examine whether feature filtering, to exclude less significant individual features, could improve predictive models. Feature filtering by *t*-test for individual methods of position specific base composition (method 1) resulted in minor improvements by cross validation on dataset_2431 _(Table 3) but no improvements when training on dataset_579 _(Table 4). By contrast, feature filtering for thermodynamics (method 2) resulted in model improvements for both datasets in either cross validation or reciprocal training and testing, tables 5 and 6. Additional improvements can be seen with guide strand structure (method 4), tables 7 and 8, guide strand structure features (method 5), tables 9 and 10. However, feature filtering for *N*-Grams *N *= 2–5 (method 11) resulted in the most dramatic model improvements for both training on dataset_2431 _(Table 11) as well as training on dataset_579 _(Table 12). Feature filtering for *N*-Grams caused the reciprocal training and testing of the datasets to be more effective when compared to the unfiltered *N*-Grams method. Target strand structure-directional (method 13) generally results in predictive models when performed without feature filtering, but improvements of model building between datasets can occur with feature filtering, tables 13 and 14.

**Table 4:** Guide strand position specific base composition (Method 1) for training RBF-epsilon regression SVM model

		train_579_		train_579_		train_579_	
		test_579_		test_2431_		test_579 _10 × cross val	
				
*t-*test	FN_579_	*R*	*MSE*	*R*	*MSE*	*R*	*MSE*
0	84(84)	0.716	0.048	**0.484**	0.054	**0.562**	0.079
1	45(45.9)	**0.718**	0.048	0.483	0.056	0.541	0.081
2	22(21)	0.645	0.057	0.467	0.058	0.449	0.091
3	8(7.1)	0.489	0.075	0.353	0.055	0.419	0.092
4	4(3.7)	0.418	0.082	0.350	0.052	0.424	0.092
5	3(2.2)	0.397	0.083	0.334	0.051	0.363	0.097
6	2(2)	0.327	0.089	0.304	0.049	0.340	0.099
7	1(0.2)	0.281	0.093	0.176	0.053	-	-
8	0(0)	-	-	-	-	-	-
9	0(0)	-	-	-	-	-	-

Models trained on dataset_579 _and testing performed with dataset_579_, dataset_2431 _and 10 × cross validation on dataset_579_, features removed by increasing stringency of *t*-test of individual feature to activity from dataset_579_.

Feature numbers in parentheses are the average number of features in cross validations.

Entries in **bold **are column maximums and are simply visual landmarks.

**Table 5:** Guide strand thermodynamics (Method 2) for training RBF-epsilon regression SVM model

		train_2431_		train_2431_		train_2431_	
		test_2431_		test_579_		test_2431 _10 × cross val	
				
*t-*test	FN_2431_	*R*	*MSE*	*R*	*MSE*	*R*	*MSE*
0	23(23)	**0.915**	0.007	0.379	0.105	0.640	0.029
1	20(20.2)	0.912	0.007	0.379	0.104	0.642	0.029
2	19(19)	0.911	0.007	0.363	0.113	0.641	0.029
3	17(17)	0.906	0.008	0.383	0.111	0.642	0.029
4	17(15.8)	0.906	0.008	0.383	0.111	0.640	0.029
5	13(10.9)	0.880	0.009	0.366	0.108	**0.650**	0.029
6	9(7.7)	0.808	0.014	0.387	0.111	0.649	0.029
7	7(6.5)	0.740	0.018	0.401	0.108	0.652	0.029
8	5(4.2)	0.666	0.022	**0.425**	0.109	0.597	0.031
9	4(3.9)	0.587	0.026	0.334	0.111	0.586	0.032

Models trained on dataset_2431 _and testing performed with dataset_2431_, dataset_579 _and 10 × cross validation on dataset_2431_, features removed by increasing stringency of *t*-test of individual feature to activity from dataset_2431_.

Feature numbers in parentheses are the average number of features in cross validations.

Entries in **bold **are column maximums and are simply visual landmarks.

**Table 6:** Guide strand thermodynamics (Method 2) for training RBF-epsilon regression SVM model

		train_579_		train_579_		train_579_	
		test_579_		test_2431_		test_579 _10 × cross val	
				
*t-*test	FN_579_	*R*	*MSE*	*R*	*MSE*	*R*	*MSE*
0	23(23)	**0.953**	0.012	0.372	0.065	0.500	0.087
1	17(15.9)	0.943	0.014	0.402	0.061	0.510	0.086
2	11(11.1)	0.886	0.023	0.330	0.061	0.548	0.082
3	8(8.2)	0.782	0.038	0.350	0.067	**0.548**	0.081
4	8(7)	0.782	0.038	0.350	0.067	0.520	0.084
5	4(4)	0.505	0.073	0.262	0.042	0.474	0.089
6	3(2.8)	0.502	0.073	0.460	0.050	0.409	0.095
7	2(1.4)	0.359	0.087	**0.462**	0.047	0.404	0.095
8	1(0.6)	0.339	0.089	0.421	0.051	-	-
9	0(0)	-	-	-	-	-	-

Models trained on dataset_579 _and testing performed with dataset_579_, dataset_2431 _and 10 × cross validation on dataset_579_, features removed by increasing stringency of *t*-test of individual feature to activity from dataset_579_.

Feature numbers in parentheses are the average number of features in cross validations.

Entries in **bold **are column maximums and are simply visual landmarks.

**Table 7:** Guide strand structure features (Method 4) for training RBF-epsilon regression SVM model

		train_2431_		train_2431_		train_2431_	
		test_2431_		test_579_		test_2431 _10 × cross val	
				
*t-*test	FN_2431_	*R*	*MSE*	*R*	*MSE*	*R*	*MSE*
0	24(24)	0.430	0.033	**0.202**	0.115	0.293	0.041
1	21(20.2)	**0.431**	0.033	0.200	0.115	0.295	0.041
2	18(17.1)	0.427	0.033	0.170	0.117	0.296	0.041
3	14(13.4)	0.396	0.034	0.158	0.113	0.291	0.041
4	9(8.1)	0.370	0.035	0.145	0.114	0.305	0.041
5	5(5.1)	0.333	0.036	0.173	0.114	**0.309**	0.041
6	4(4)	0.327	0.036	0.167	0.113	0.305	0.041
7	4(3.7)	0.327	0.036	0.167	0.113	0.304	0.041
8	3(2.9)	0.288	0.037	0.195	0.113	0.298	0.041
9	2(1)	0.270	0.037	0.200	0.113	0.262	0.042

Models trained on dataset_2431 _and testing performed with dataset_2431_, dataset_579 _and 10 × cross validation on dataset_2431_, features removed by increasing stringency of *t*-test of individual feature to activity from dataset_2431_.

Feature numbers in parentheses are the average number of features in cross validations.

Entries in **bold **are column maximums and are simply visual landmarks.

**Table 8:** Guide strand structure features (Method 4) for training RBF-epsilon regression SVM model

		train_579_		train_579_		train_579_	
		test_579_		test_2431_		test_579 _10 × cross val	
				
*t-*test	FN_579_	*R*	*MSE*	*R*	*MSE*	*R*	*MSE*
0	24(24)	**0.445**	0.079	0.214	0.073	0.214	0.106
1	16(15.4)	0.433	0.080	0.187	0.081	0.212	0.106
2	8(7.5)	0.319	0.089	0.230	0.059	0.251	0.105
3	5(4.5)	0.308	0.089	0.210	0.059	**0.259**	0.104
4	3(2.2)	0.259	0.093	**0.235**	0.056	-	-
5	0(0)	-	-	-	-	-	-
6	0(0)	-	-	-	-	-	-
7	0(0)	-	-	-	-	-	-
8	0(0)	-	-	-	-	-	-
9	0(0)	-	-	-	-	-	-

Models trained on dataset_579 _and testing performed with dataset_579_, dataset_2431 _and 10 × cross validation on dataset_579_, features removed by increasing stringency of *t*-test of individual feature to activity from dataset_579_.

Feature numbers in parentheses are the average number of features in cross validations.

Entries in **bold **are column maximums and are simply visual landmarks.

**Table 9:** Guide strand Xue features (Method 5) for training RBF-epsilon regression SVM model

		train_2431_		train_2431_		train_2431_	
		test_2431_		test_579_		test_2431 _10 × cross val	
				
*t-*test	FN_2431_	*R*	*MSE*	*R*	*MSE*	*R*	*MSE*
0	32(32)	**0.266**	0.037	**0.214**	0.112	**0.243**	0.042
1	27(25.4)	0.261	0.037	0.205	0.113	0.233	0.042
2	16(15.2)	0.255	0.038	0.192	0.113	0.226	0.043
3	10(9.4)	0.247	0.038	0.187	0.113	0.221	0.043
4	6(6.4)	0.237	0.038	0.182	0.113	0.217	0.043
5	4(3.9)	0.200	0.038	0.152	0.114	0.202	0.043
6	3(2.6)	0.187	0.039	0.145	0.114	0.196	0.043
7	2(1.7)	0.187	0.039	0.145	0.114	-	-
8	1(0.3)	0.158	0.039	0.089	0.114	-	-
9	0(0)	-	-	-	-	-	-

Models trained on dataset_2431 _and testing performed with dataset_2431_, dataset_579 _and 10 × cross validation on dataset_2431_, features removed by increasing stringency of *t*-test of individual feature to activity from dataset_2431_.

Feature numbers in parentheses are the average number of features in cross validations.

Entries in **bold **are column maximums and are simply visual landmarks.

**Table 10:** Guide strand Xue features (Method 5) for training RBF-epsilon regression SVM model

		train_579_		train_579_		train_579_	
		test_579_		test_2431_		test_579 _10 × cross val	
				
*t-*test	FN_579_	*R*	*MSE*	*R*	*MSE*	*R*	*MSE*
0	32(32)	0.207	0.096	0.164	0.046	0.194	0.107
1	20(18.8)	**0.214**	0.095	0.167	0.046	**0.198**	0.107
2	8(7.9)	0.212	0.095	**0.170**	0.048	**0.198**	0.108
3	1(1.2)	0.141	0.097	0.155	0.047	-	-
4	0(0)	-	-	-	-	-	-
5	0(0)	-	-	-	-	-	-
6	0(0)	-	-	-	-	-	-
7	0(0)	-	-	-	-	-	-
8	0(0)	-	-	-	-	-	-
9	0(0)	-	-	-	-	-	-

Models trained on dataset_579 _and testing performed with dataset_579_, dataset_2431 _and 10 × cross validation on dataset_579_, features removed by increasing stringency of *t*-test of individual feature to activity from dataset_579_.

Feature numbers in parentheses are the average number of features in cross validations.

Entries in **bold **are column maximums and are simply visual landmarks.

**Table 11:** Guide strand *N*-Grams (Method 11) for training RBF-epsilon regression SVM model

		train_2431_		train_2431_		train_2431_	
		test_2431_		test_579_		test_2431 _10 × cross val	
				
*t-*test	FN_2431_	*R*	*MSE*	*R*	*MSE*	*R*	*MSE*
0	1360(1360)	**0.614**	0.026	0.246	0.109	**0.559**	0.033
1	777(771.3)	0.604	0.026	**0.596**	0.069	0.526	0.034
2	424(394.3)	0.590	0.027	0.576	0.070	0.471	0.036
3	174(160.7)	0.533	0.029	0.516	0.075	0.391	0.039
4	71(59.5)	0.490	0.031	0.477	0.077	0.343	0.040
5	27(22.5)	0.404	0.033	0.408	0.082	0.295	0.041
6	9(7.1)	0.319	0.036	0.311	0.090	0.294	0.041
7	5(3.7)	0.291	0.037	0.246	0.094	0.268	0.042
8	2(1.7)	0.228	0.038	0.187	0.096	-	-
9	0(0)	-	-	-	-	-	-

Models trained on dataset_2431 _and testing performed with dataset_2431_, dataset_579 _and 10 × cross validation on dataset_2431_, features removed by increasing stringency of *t*-test of individual feature to activity from dataset_2431_.

Feature numbers in parentheses are the average number of features in cross validations.

Entries in **bold **are column maximums and are simply visual landmarks.

**Table 12:** Guide strand *N*-Grams (Method 11) for training RBF-epsilon regression SVM model

		train_579_		train_579_		train_579_	
		test_579_		test_2431_		test_579 _10 × cross val	
				
*t-*test	FN_579_	*R*	*MSE*	*R*	*MSE*	*R*	*MSE*
0	1360(1360)	0.615	0.070	0.192	0.055	**0.469**	0.088
1	591(586.1)	0.641	0.063	**0.566**	0.028	0.421	0.093
2	195(179.3)	**0.644**	0.060	0.467	0.032	0.431	0.091
3	42(37)	0.502	0.073	0.340	0.035	0.323	0.099
4	7(6.4)	0.370	0.085	0.212	0.038	0.224	0.105
5	3(1.5)	0.250	0.093	0.094	0.040	-	-
6	0(0)	-	-	-	-	**-**	-
7	0(0)	-	-	-	-	-	-
8	0(0)	-	-	-	-	-	-
9	0(0)	-	-	-	-	-	-

Models trained on dataset_579 _and testing performed with dataset_579_, dataset_2431 _and 10 × cross validation on dataset_579_, features removed by increasing stringency of *t*-test of individual feature to activity from dataset_579_.

Feature numbers in parentheses are the average number of features in cross validations.

Entries in **bold **are column maximums and are simply visual landmarks.

**Table 13:** Target strand secondary structure (Method 13) for training RBF-epsilon regression SVM model

		train_2431_		train_2431_		train_2431_	
		test_2431_		test_579_		test_2431 _10 × cross val	
				
*t-*test	FN_2431_	*R*	*MSE*	*R*	*MSE*	*R*	*MSE*
0	43(43)	**0.623**	0.025	0.045	0.111	0.277	0.042
1	28(25.9)	0.612	0.026	0.032	0.111	0.285	0.042
2	13(11.4)	0.530	0.029	0.045	0.110	0.313	0.041
3	8(7.4)	0.479	0.031	**0.071**	0.109	**0.317**	0.041
4	3(3.3)	0.401	0.034	0.048	0.110	0.308	0.041
5	1(1.2)	0.327	0.036	0.045	0.110	0.282	0.041
6	1(1)	0.327	0.036	0.045	0.110	0.287	0.041
7	1(1)	0.327	0.036	0.045	0.110	0.287	0.041
8	1(1)	0.327	0.036	0.045	0.110	0.287	0.041
9	1(1)	0.327	0.036	0.045	0.110	0.287	0.041

Models trained on dataset_2431 _and testing performed with dataset_2431_, dataset_579 _and 10 × cross validation on dataset_2431_, features removed by increasing stringency of *t*-test of individual feature to activity from dataset_2431_.

Feature numbers in parentheses are the average number of features in cross validations.

Entries in **bold **are column maximums and are simply visual landmarks.

**Table 14:** Target strand secondary structure (Method 13) for training RBF-epsilon regression SVM model

		train_579_		train_579_		train_579_	
		test_579_		test_2431_		test_579 _10 × cross val	
				
*t-*test	FN_579_	*R*	*MSE*	*R*	*MSE*	*R*	*MSE*
0	43(43)	**0.673**	0.055	0.070	0.104	**0.262**	0.105
1	21(19.7)	0.408	0.082	0.077	0.059	0.194	0.107
2	5(5.9)	0.282	0.091	**0.100**	0.053	0.095	0.111
3	3(2.1)	0.187	0.097	0.070	0.048	0.068	0.111
4	0(0.1)	-	-	-	-	-	-
5	0(0)	-	-	-	-	-	-
6	0(0)	-	-	-	-	**-**	-
7	0(0)	-	-	-	-	-	-
8	0(0)	-	-	-	-	-	-
9	0(0)	-	-	-	-	-	-

Models trained on dataset_579 _and testing performed with dataset_579_, dataset_2431 _and 10 × cross validation on dataset_579_, features removed by increasing stringency of *t*-test of individual feature to activity from dataset_579_.

Feature numbers in parentheses are the average number of features in cross validations.

Entries in **bold **are column maximums and are simply visual landmarks.

### III a. combining feature mapping methods

The position specific base composition, thermodynamics and *N*-Gram feature-mapping methods yield predictive models on separate training and testing datasets (methods 1, 2 and 11). Combining methods 1, 2 and 11 resulted in the improved accuracy of SVM models during training and testing, tables 15 and 16, when compared to each method individually (Tables 3, 4, 5, 6, 11 and 12). However, pair wise combinations of methods 1, 2 and 11 can result in models that are more effective when compared to models constructed with more features. The modeling results for methods 1 and 2 combined are presented in tables 17 and 18. Likewise the modeling results for methods 2 and 11 combined are in tables 19 and 20 and methods 1 and 11 combined are in tables 21 and 22. Methods that limit the additional modeling features can result in more effective models when compared to models constructed with more features. For example, by CV within dataset_2431 _the maximal predictive model from methods 1, 2 and 11 results in *R *= 0.767 *MSE *= 0.023, Table 15. Whereas a higher predictive model can be generated by CV within dataset_2431 _with just methods 1 and 11 combined, *R *= 0.784 *MSE *= 0.022, Table 21. A similar, but less dramatic, pattern can be seen within dataset_579 _CV, where a model constructed with methods 1, 2 and 11 performed *R *= 0.662, *MSE *= 0.070, table 16, but a model constructed with methods 2 and 11 performed *R *= 0.669, *MSE *= 0.068, table 20.

**Table 15:** Combining position specific composition (Method 1), thermodynamics (Method 2) and *N*-Gram (Method 11) for training RBF-epsilon regression SVM model

		train_2431_		train_2431_		train_2431_	
		test_2431_		test_579_		test_2431 _10 × cross val	
				
*t-*test	FN_2431_	*R*	*MSE*	*R*	*MSE*	*R*	*MSE*
0	1467(1467)	0.793	0.015	0.518	0.098	**0.767**	0.023
1	862(855.7)	0.802	0.014	0.506	0.089	0.764	0.023
2	488(457.2)	0.809	0.014	**0.528**	0.091	0.750	0.023
3	223(208)	0.817	0.013	0.526	0.091	0.728	0.025
4	108(94.2)	**0.840**	0.012	0.498	0.094	0.712	0.026
5	55(48.1)	**0.840**	0.012	0.495	0.096	0.711	0.026
6	29(23.1)	0.817	0.013	0.503	0.098	0.696	0.026
7	17(15.2)	0.770	0.016	0.444	0.099	0.673	0.027
8	11(9.6)	0.702	0.020	0.452	0.106	0.616	0.030
9	6(5.9)	0.606	0.025	0.340	0.109	0.603	0.031

Models trained on dataset_2431 _and testing performed with dataset_2431_, dataset_579 _and 10 × cross validation on dataset_2431_, features removed by increasing stringency of *t*-test of individual feature to activity from dataset_2431_.

Feature numbers in parentheses are the average number of features in cross validations.

Entries in **bold **are column maximums and are simply visual landmarks.

**Table 16:** Combining position specific composition (Method 1), thermodynamics (Method 2) and *N*-Gram (Method 11) for training RBF-epsilon regression SVM model

		train_579_		train_579_		train_579_	
		test_579_		test_2431_		test_579 _10 × cross val	
				
*t-*test	FN_579_	*R*	*MSE*	*R*	*MSE*	R	*MSE*
0	1467(1467)	0.753	0.046	**0.546**	0.064	**0.662**	0.070
1	653(647.9)	0.780	0.041	0.537	0.056	0.647	0.069
2	228(211.4)	**0.792**	0.038	0.521	0.058	0.640	0.071
3	58(52.3)	0.778	0.039	0.491	0.086	0.613	0.074
4	19(17.1)	**0.792**	0.037	0.452	0.077	0.569	0.079
5	10(7.7)	0.581	0.064	0.361	0.041	0.504	0.085
6	5(4.8)	0.525	0.071	0.443	0.052	0.424	0.094
7	3(1.6)	0.374	0.086	0.436	0.050	0.405	0.095
8	1(0.6)	0.339	0.089	0.422	0.051	-	-
9	0(0)	-	-	-	-	-	-

Models trained on dataset_579 _and testing performed with dataset_579_, dataset_2431 _and 10 × cross validation on dataset_579_, features removed by increasing stringency of *t*-test of individual feature to activity from dataset_579_.

Feature numbers in parentheses are the average number of features in cross validations.

Entries in **bold **are column maximums and are simply visual landmarks.

**Table 17:** Combining position specific composition (Method 1) and thermodynamics (Method 2) for training RBF-epsilon regression SVM model

		train_2431_		train_2431_		train_2431_	
		test_2431_		test_579_		test_2431 _10 × cross val	
				
*t-*test	FN_2431_	*R*	*MSE*	*R*	*MSE*	*R*	*MSE*
0	107(107)	0.878	0.009	0.450	0.104	0.701	0.026
1	85(84.5)	0.882	0.009	0.435	0.098	0.705	0.026
2	64(62.9)	0.886	0.009	0.444	0.105	0.704	0.026
3	49(47.3)	0.884	0.009	0.463	0.105	0.699	0.026
4	37(34.7)	**0.889**	0.009	0.447	0.105	0.698	0.026
5	28(25.6)	0.866	0.010	0.453	0.105	**0.707**	0.026
6	20(16)	0.822	0.013	**0.472**	0.106	0.685	0.027
7	12(11.5)	0.757	0.017	0.417	0.107	0.672	0.028
8	9(7.9)	0.684	0.021	0.439	0.107	0.614	0.030
9	6(5.9)	0.606	0.025	0.340	0.109	0.603	0.031

Models trained on dataset_2431 _and testing performed with dataset_2431_, dataset_579 _and 10 × cross validation on dataset_2431_, features removed by increasing stringency of *t*-test of individual feature to activity from dataset_2431_.

Feature numbers in parentheses are the average number of features in cross validations.

Entries in **bold **are column maximums and are simply visual landmarks.

**Table 18:** Combining position specific composition (Method 1) and thermodynamics (Method 2) for training RBF-epsilon regression SVM model

		train_579_		train_579_		train_579_	
		test_579_		test_2431_		test_579 _10 × cross val	
				
*t-*test	FN_579_	*R*	*MSE*	*R*	*MSE*	*R*	*MSE*
0	107(107)	0.874	0.026	**0.493**	0.083	0.533	0.083
1	62(61.8)	**0.889**	0.023	0.486	0.083	0.521	0.085
2	33(32.1)	0.856	0.028	0.466	0.069	0.537	0.083
3	16(15.3)	0.816	0.034	0.388	0.087	**0.559**	0.081
4	12(10.7)	0.796	0.036	0.412	0.072	0.527	0.084
5	7(6.2)	0.537	0.069	0.288	0.051	0.497	0.087
6	5(4.8)	0.525	0.071	0.442	0.052	0.424	0.094
7	3(1.6)	0.374	0.086	0.435	0.050	0.405	0.095
8	1(0.6)	0.339	0.089	0.421	0.051	-	-
9	0(0)	-	-	-	-	-	-

Models trained on dataset_579 _and testing performed with dataset_579_, dataset_2431 _and 10 × cross validation on dataset_579_, features removed by increasing stringency of *t*-test of individual feature to activity from dataset_579_.

Feature numbers in parentheses are the average number of features in cross validations.

Entries in **bold **are column maximums and are simply visual landmarks.

**Table 19:** Combining thermodynamics (Method 2) and *N*-Grams (Method 11) for training RBF-epsilon regression SVM model

		train_2431_		train_2431_		train_2431_	
		test_2431_		test_579_		test_2431 _10 × cross val	
				
*t-*test	FN_2431_	*R*	*MSE*	*R*	*MSE*	*R*	*MSE*
0	1383(1383)	0.746	0.018	**0.491**	0.101	**0.721**	0.025
1	797(791.5)	0.755	0.017	0.480	0.093	0.709	0.026
2	443(413.3)	0.763	0.017	0.480	0.094	0.688	0.027
3	191(177.7)	0.773	0.016	0.462	0.097	0.671	0.028
4	88(75.3)	0.812	0.014	0.448	0.100	0.656	0.028
5	40(33.4)	**0.818**	0.013	0.442	0.097	0.659	0.028
6	18(14.8)	0.789	0.015	0.448	0.096	0.659	0.028
7	12(10.2)	0.759	0.017	0.435	0.100	0.656	0.028
8	7(5.9)	0.686	0.021	0.428	0.106	0.600	0.031
9	4(3.9)	0.587	0.026	0.335	0.111	0.586	0.032

Models trained on dataset_2431 _and testing performed with dataset_2431_, dataset_579 _and 10 × cross validation on dataset_2431_, features removed by increasing stringency of *t*-test of individual feature to activity from dataset_2431_.

Feature numbers in parentheses are the average number of features in cross validations.

Entries in **bold **are column maximums and are simply visual landmarks.

**Table 20:** Combining thermodynamics (Method 2) and *N*-Grams (Method 11) for training RBF-epsilon regression SVM model

		train_579_		train_579_		train_579_	
		test_579_		test_2431_		test_579 _10 × cross val	
				
*t-*test	FN_579_	*R*	*MSE*	*R*	*MSE*	*R*	*MSE*
0	1383(1383)	0.726	0.050	**0.521**	0.067	**0.669**	0.068
1	608(602)	0.759	0.044	0.506	0.052	0.651	0.069
2	206(190.4)	0.779	0.041	0.489	0.051	0.641	0.071
3	50(45.2)	0.758	0.042	0.488	0.067	0.601	0.076
4	15(13.4)	**0.783**	0.038	0.427	0.059	0.551	0.081
5	7(5.5)	0.553	0.068	0.310	0.041	0.486	0.088
6	3(2.8)	0.503	0.073	0.460	0.050	0.409	0.095
7	2(1.4)	0.359	0.087	0.463	0.047	0.404	0.095
8	1(0.6)	0.339	0.089	0.422	0.051	-	-
9	0(0)	-	-	-	-	-	-

Models trained on dataset_579 _and testing performed with dataset_579_, dataset_2431 _and 10 × cross validation on dataset_579_, features removed by increasing stringency of *t*-test of individual feature to activity from dataset_579_.

Feature numbers in parentheses are the average number of features in cross validations.

Entries in **bold **are column maximums and are simply visual landmarks.

**Table 21:** Combining position specific base composition (Method 1) and *N*-Grams (Method 11) for training RBF-epsilon regression SVM model

		train_2431_		train_2431_		train_2431_	
		test_2431_		test_579_		test_2431 _10 × cross val	
				
*t-*test	FN_2431_	*R*	*MSE*	*R*	*MSE*	*R*	*MSE*
0	1444(1444)	**0.783**	0.017	**0.492**	0.096	**0.784**	0.022
1	842(835.5)	0.782	0.016	0.479	0.098	0.782	0.022
2	469(438.2)	0.777	0.016	0.485	0.102	0.765	0.023
3	206(191)	0.754	0.017	0.471	0.102	0.731	0.024
4	91(78.4)	0.736	0.018	0.459	0.103	0.702	0.026
5	42(37.2)	0.700	0.020	0.459	0.102	0.677	0.027
6	20(15.4)	0.658	0.023	0.445	0.106	0.626	0.030
7	10(8.7)	0.553	0.028	0.344	0.105	0.567	0.032
8	6(5.4)	0.512	0.029	0.356	0.103	0.525	0.034
9	2(2)	0.407	0.033	0.257	0.108	0.454	0.037

Models trained on dataset_2431 _and testing performed with dataset_2431_, dataset_579 _and 10 × cross validation on dataset_2431_, features removed by increasing stringency of *t*-test of individual feature to activity from dataset_2431_.

Feature numbers in parentheses are the average number of features in cross validations.

Entries in **bold **are column maximums and are simply visual landmarks.

**Table 22:** Combining position specific base composition (Method 1) and *N*-Grams (Method 11) for training RBF-epsilon regression SVM model

		train_579_		train_579_		train_579_	
		test_579_		test_2431_		test_579 _10 × cross val	
				
*t-*test	FN_579_	*R*	*MSE*	*R*	*MSE*	*R*	*MSE*
0	1444(1444)	0.751	0.054	0.423	0.051	**0.646**	0.070
1	636(632)	**0.760**	0.046	**0.440**	0.057	0.632	0.071
2	217(200.3)	0.746	0.046	0.431	0.062	0.556	0.080
3	50(44.1)	0.636	0.058	0.333	0.064	0.513	0.084
4	11(10.1)	0.532	0.071	0.381	0.055	0.460	0.089
5	6(3.7)	0.465	0.077	0.327	0.052	0.385	0.095
6	2(2)	0.327	0.089	0.305	0.049	0.340	0.099
7	1(0.2)	0.281	0.093	0.176	0.053	-	-
8	0(0)	-	-	-	-	-	-
9	0(0)	-	-	-	-	-	-

Models trained on dataset_579 _and testing performed with dataset_579_, dataset_2431 _and 10 × cross validation on dataset_579_, features removed by increasing stringency of *t*-test of individual feature to activity from dataset_579_.

Feature numbers in parentheses are the average number of features in cross validations.

Entries in **bold **are column maximums and are simply visual landmarks.

Several features such as the guide strand structure and guide strand structure features (methods 4 and 5) individually contribute to positively predictive SVM models when training and testing is within datasets by CV or between datasets. Combining feature mapping methods 4 and 5 with methods 1, 2 and 11 (Tables 23 and 24) had little positive influence on the general predictive ability of the models' ability to predict data not seen during model building. With the exception of training on dataset_579 _and testing on dataset_2431_, performance generally degraded from combining methods 1, 2 and 11 to combining methods 1,2,4,5 and 11, comparing tables 8 and 12. A similar pattern is seen by adding method 13, tables 25 and 26. Overall adding predictive features to models did not result in a deterministic improvement of model performance, but when models incorporate large number of features there are some benefits to feature filtering to improve model performance.

**Table 23:** Combining position specific composition (Method 1), thermodynamics (Method 2), *N*-Gram (Method 11), guide strand structure (Method 4) and Xue features (Method 5) for training RBF-epsilon regression SVM model

		train_2431_		train_2431_		train_2431_	
		test_2431_		test_579_		test_2431 _10 × cross val	
				
*t-*test	FN_2431_	*R*	*MSE*	*R*	*MSE*	*R*	*MSE*
0	1523(1523)	0.797	0.015	0.523	0.099	**0.760**	0.023
1	910(901.3)	0.807	0.014	0.513	0.090	**0.760**	0.023
2	522(489.5)	0.817	0.013	**0.526**	0.092	0.746	0.024
3	247(230.8)	0.825	0.013	0.518	0.092	0.726	0.025
4	123(108.7)	0.850	0.011	0.495	0.097	0.710	0.026
5	64(57.1)	**0.856**	0.011	0.504	0.097	0.709	0.026
6	36(29.7)	0.844	0.012	0.504	0.099	0.695	0.026
7	23(20.6)	0.816	0.013	0.449	0.100	0.675	0.027
8	15(12.8)	0.738	0.018	0.457	0.105	0.618	0.030
9	8(6.9)	0.661	0.022	0.334	0.109	0.606	0.031

Models trained on dataset_2431 _and testing performed with dataset_2431_, dataset_579 _and 10 × cross validation on dataset_2431_, features removed by increasing stringency of *t*-test of individual feature to activity from dataset_2431_.

Feature numbers in parentheses are the average number of features in cross validations.

Entries in **bold **are column maximums and are simply visual landmarks.

**Table 24:** Combining position specific composition (Method 1), thermodynamics (Method 2), *N*-Gram (Method 11), guide strand structure (Method 4) and Xue features (Method 5) for training RBF-epsilon regression SVM model

		train_579_		train_579_		train_579_	
		test_579_		test_2431_		test_579 _10 × cross val	
				
*t-*test	FN_579_	*R*	*MSE*	*R*	*MSE*	*R*	*MSE*
0	1523(1523)	0.754	0.046	**0.548**	0.067	**0.643**	0.070
1	689(682.1)	0.786	0.040	0.543	0.055	0.638	0.071
2	244(226.8)	0.798	0.037	0.516	0.061	0.639	0.071
3	64(58)	0.801	0.036	0.506	0.079	0.612	0.074
4	22(19.3)	**0.819**	0.034	0.453	0.074	0.563	0.080
5	10(7.7)	0.580	0.064	0.360	0.041	0.504	0.085
6	5(4.8)	0.525	0.071	0.442	0.052	0.424	0.094
7	3(1.6)	0.374	0.086	0.435	0.050	0.405	0.095
8	1(0.6)	0.339	0.089	0.421	0.051	-	-
9	0(0)	-	-	-	-	-	-

Models trained on dataset_579 _and testing performed with dataset_579_, dataset_2431 _and 10 × cross validation on dataset_579_, features removed by increasing stringency of *t*-test of individual feature to activity from dataset_579_.

Feature numbers in parentheses are the average number of features in cross validations.

Entries in **bold **are column maximums and are simply visual landmarks.

**Table 25:** Combining position specific composition (Method 1), thermodynamics (Method 2),*N*-Gram (Method 11), guide strand structure (Method 4) Xue features (Method 5) and target secondary structure (Method 13) for training RBF-epsilon regression SVM model

		train_2431_		train_2431_		train_2431_	
		test_2431_		test_579_		test_2431 _10 × cross val	
				
*t-*test	FN_2431_	*R*	*MSE*	*R*	*MSE*	*R*	*MSE*
0	1566(1566)	0.826	0.013	0.523	0.099	0.710	0.027
1	938(927.2)	0.842	0.012	0.514	0.090	0.723	0.026
2	535(500.9)	0.856	0.011	**0.526**	0.092	**0.728**	0.025
3	255(238.2)	0.873	0.010	0.519	0.092	0.718	0.025
4	126(112)	0.902	0.008	0.496	0.097	0.705	0.026
5	65(58.3)	0.912	0.007	0.505	0.097	0.701	0.026
6	37(30.7)	**0.913**	0.007	0.505	0.099	0.687	0.027
7	24(21.6)	0.908	0.007	0.449	0.100	0.672	0.028
8	16(13.8)	0.884	0.009	0.457	0.105	0.615	0.030
9	9(7.9)	0.862	0.011	0.335	0.109	0.605	0.031

Models trained on dataset_2431 _and testing performed with dataset_2431_, dataset_579 _and 10 × cross validation on dataset_2431_, features removed by increasing stringency of *t*-test of individual feature to activity from dataset_2431_.

Feature numbers in parentheses are the average number of features in cross validations.

Entries in **bold **are column maximums and are simply visual landmarks.

**Table 26:** Combining position specific composition (Method 1), thermodynamics (Method 2), *N*-Gram (Method 11), guide strand structure (Method 4) Xue features (Method 5) and target secondary structure (Method 13) for training RBF-epsilon regression SVM model

		train_579_		train_579_		train_579_	
		test_579_		test_2431_		test_579 _10 × cross val	
				
*t-*test	FN_579_	*R*	*MSE*	*R*	*MSE*	*R*	*MSE*
0	1566(1566)	0.791	0.041	**0.549**	0.067	0.613	0.076
1	710(701.8)	0.796	0.039	0.543	0.055	**0.638**	0.071
2	249(232.7)	0.801	0.037	0.517	0.061	0.628	0.072
3	67(60.1)	0.802	0.036	0.507	0.079	0.606	0.075
4	22(19.4)	**0.820**	0.034	0.454	0.074	0.561	0.080
5	10(7.7)	0.581	0.064	0.361	0.041	0.504	0.085
6	5(4.8)	0.525	0.071	0.443	0.052	0.424	0.094
7	3(1.6)	0.374	0.086	0.436	0.050	0.405	0.095
8	1(0.6)	0.339	0.089	0.422	0.051	-	-
9	0(0)	-	-	-	-	-	-

Models trained on dataset_579 _and testing performed with dataset_579_, dataset_2431 _and 10 × cross validation on dataset_579_, features removed by increasing stringency of *t*-test of individual feature to activity from dataset_579_.

Feature numbers in parentheses are the average number of features in cross validations.

Entries in **bold **are column maximums and are simply visual landmarks.

### III b. feature selection on multiple feature derived models

The overall effects of combining feature mapping methods and feature filtering to increase the predictive accuracy of SVM models construction are summarized in Table 27. There are multiple feature set and filtering optima (italicized in table 27), depending on the criteria desired for model construction. For example, if the intent is to construct a model that best predicts dataset_2431 _by CV, choosing methods 1 and 11 without feature filtering results in a maximal *R *= 0.784 and *MSE *= 0.022. However, if the intention is to construct a model that best predicts dataset_2431 _by training on dataset_579_, combining methods 1, 2, 4, 5, 11 and 13 without feature filtering results in a highly predictive model, *R *= 0.549 and *MSE *= 0.067.

**Table 27:** Combining and filtering features for training RBF-epsilon regression SVM model on dataset_2431_ and on dataset_579_

		train_2431_					train_579_				
			test_579_		test_2431 _10 × cross val			test_2431_		test_579 _10 × cross val	
							
Method(s)	*t-*test	FN_2431_	*R*	*MSE*	*R*	*MSE*	FN_579_	*R*	*MSE*	*R*	*MSE*
1	0	84(84)	**0.509**	0.095	0.711	0.026	84(84)	**0.484**	0.054	**0.562**	0.079
1	2	45(43.9)	0.494	0.100	0.687	0.027	22(21)	0.467	0.058	0.449	0.091
2	0	23(23)	0.379	0.105	0.640	0.029	23(23)	0.372	0.065	0.500	0.087
2	2	19(19)	0.363	0.113	0.641	0.029	11(11.1)	0.330	0.061	0.548	0.082
11	0	1360(1360)	0.246	0.109	**0.559**	0.033	1360(1360)	0.192	0.055	**0.469**	0.088
11	2	424(394.3)	0.576	0.070	0.471	0.036	195(179.3)	0.467	0.032	0.431	0.091
1,2	0	107(107)	0.450	0.104	0.701	0.026	107(107)	**0.493**	0.083	0.533	0.083
1,2	2	64(62.9)	0.444	0.105	0.704	0.026	33(32.1)	0.466	0.069	0.537	0.083
2,11	0	1383(1383)	**0.491**	0.101	**0.721**	0.025	1383(1383)	**0.521**	0.067	***0.669***	*0.068*
2,11	2	443(413.3)	0.480	0.094	0.688	0.027	206(190.4)	0.489	0.051	0.641	0.071
1,11	0	1444(1444)	**0.492**	0.096	***0.784***	*0.022*	1444(1444)	0.423	0.051	**0.646**	0.070
1,11	2	469(438.2)	0.485	0.102	0.765	0.023	217(200.3)	0.431	0.062	0.556	0.080
1,2,11	0	1467(1467)	0.518	0.098	**0.767**	0.023	1467(1467)	**0.546**	0.064	**0.662**	0.070
1,2,11	2	488(457.2)	***0.528***	*0.091*	0.750	0.023	228(211.4)	0.521	0.058	0.640	0.071
1,2,4,5,11	0	1523(1523)	0.523	0.099	**0.760**	0.023	1523(1523)	**0.548**	0.067	**0.643**	0.070
1,2,4,5,11	2	522(489.5)	**0.526**	0.092	0.746	0.024	244(226.8)	0.516	0.061	0.639	0.071
1,2,4,5,11,13	0	1566(1566)	0.523	0.099	0.710	0.027	1566(1566)	***0.549***	*0.067*	0.613	0.076
1,2,4,5,11,13	2	535(500.9)	**0.526**	0.092	**0.728**	0.025	249(232.7)	0.517	0.061	0.628	0.072

Method numbers are from Table 1.

Models trained on dataset_2431 _and testing performed with dataset_579 _and 10 × cross validation on dataset_2431_, features removed by increasing stringency of *t*-test of individual feature to activity from dataset_2431_.

Models trained on dataset_579 _and testing performed with dataset_2431 _and 10 × cross validation on dataset_579_, features removed by increasing stringency of *t*-test of individual feature to activity from dataset_579_.

Feature numbers in parentheses are the average number of features in cross validations.

Entries in **bold **are column maximums from their respective tables and are provided as visual indicators. *Italicized *entries are column maximums within the table and are again provided as visual indicators.

Feature set selection and feature subset selection is clearly influential on model performance. We investigated whether the implementation of an algorithmic method of feature subset selection could result in improved model construction. For this exploration, dataset_2431 _and dataset_579 _were used as training and testing sets by CV and all features from methods 1, 2, 4, 5, 11 and 13 were included in the starting pool of 1566 candidate features. Features were then selected for inclusion based on the Correlation Feature Selection (CFS) method. Briefly, CFS is a maximum-relevance minimum-redundancy method and greedily adds features to maximize the growth of equation 3, where the numerator is the feature to outcome correlation and the denominator is comprised of the feature to feature cross correlation. Models constructed in this fashion had an average *R *= 0.720 with *MSE *= 0.024, by CV within dataset_2431 _and models contained on average 680.1 features. Likewise performing CFS on dataset_579 _starting with 1566 features resulted in models with an average *R *= 0.603 and *MSE *= 0.074 and an average of 505.7 features.

**Figure 5:**
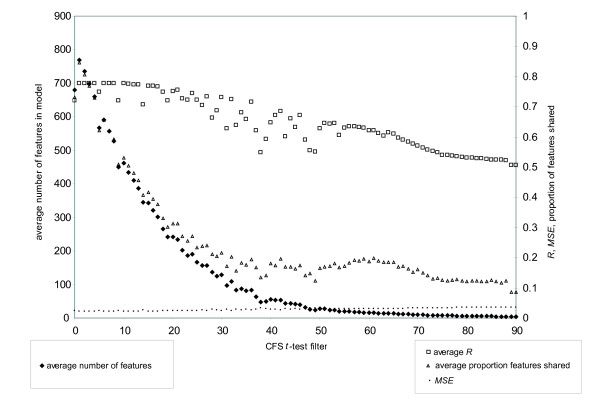
**Correlation based Feature Selection (CFS) filtering by cross validation within dataset_2431_**. Solid diamonds are the average number of features resulting from the CFS models graphed to the left y-axis. Open squares are the average CFS model correlations (*R*) graphed to the right y-axis. Open triangles are the average pair wise fraction of features found in common between CFS models by cross validation, graphed to the right y-axis. Closed small circles are the mean squared errors (*MSE*) of the models, graphed to the right y-axis.

**Figure 6:**
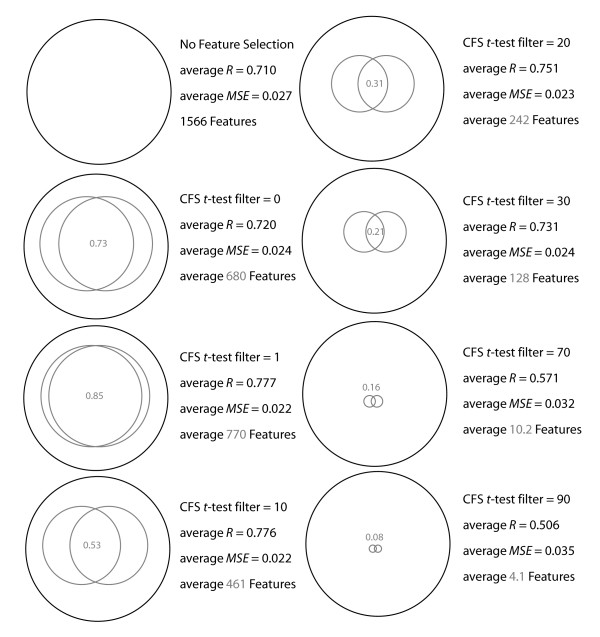
**Venn diagrams representing the relationships among feature sets and subsets, and their model outcomes by cross validation within dataset_2431_**. The black large circles representing the space of all 1566 possible features, formally this is set *S*_*all *_with cardinality of 1566. The smaller grey circles represent the feature subsets found by CFS selection and the intersections of the grey circles represent the features that were on average consistently found between pair wise comparisons among the cross validations. Specifically, *S*_*2431-A *_and *S*_*2431-B *_are the grey circles and they represent the average of pair wise comparisons of feature subsets found by cross validation on dataset_2431 _where *S*_*2431-A*_⊂*S*_*all *_and *S*_*2431-B*_⊂ *S*_*all *_and the average sub set cardinality is represented by the diameter of the grey circle and the *S*_*2431-A *_∩ *S*_*2431-B *_is provided as the average fraction of features shared between sub sets.

To investigate whether improvements in model predictability and model interpretability could be made the candidate feature set was filtered. Features were included in models by retaining only the most significant features, by *t*-test of feature to outcome, in the training model and then testing the resulting model on the naive testing data partition (Figure 5). Eliminating features from the 1566 candidate features at a *t*-test filter of 1 can result in maximally predictive models with an average *R *= 0.777 and an average of 769.6 features in the final model, by cross validation within dataset_2431_. On average the same features were consistently found in 0.845 of pair wise comparisons among models from the training and testing sets within dataset_2431_. Further increasing the stringency for feature inclusion can result in highly predictive models with substantially fewer features (Figure 5). However, as feature inclusion stringency increased, the commonality of features found between models declined. For example, a CFS *t*-test filter of 10, results in a nearly maximal predictive model with *R *= 0.776 and *MSE *= 0.022, and reduces the average number of features in each model to 461.4, but only half (0.531) of the features are consistently found in pair wise comparisons among the resulting predictive models. The effect of feature subset selection and the resulting feature models are summarized in the Venn diagrams in Figure 6. It is evident that reducing the number of features within a model can improve model performance, and can also yield multiple nearly equally predictive models. Feature commonality among equally predictive models decreases as only the most significant features are considered for model inclusion.

**Figure 7:**
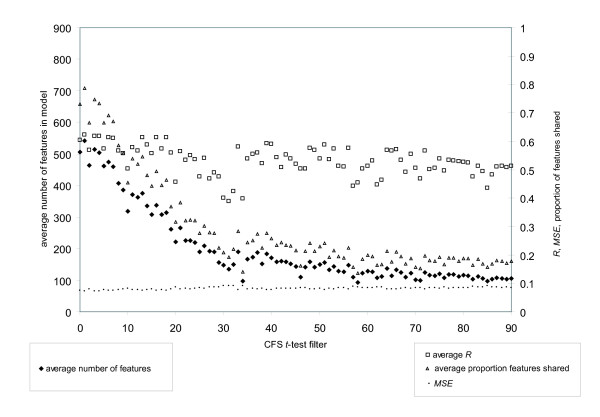
**Correlation based Feature Selection (CFS) filtering by cross validation within dataset_579_**. Solid diamonds are the average number of features resulting from the CFS models graphed to the left y-axis. Open squares are the average CFS model correlations (*R*) graphed to the right y-axis. Open triangles are the average pair wise fraction of features found in common between CFS models by cross validation, graphed to the right y-axis. Closed small circles are the mean squared errors (*MSE*) of the models, graphed to the right y-axis.

Similar findings are found by performing CFS within dataset_579_. First, an improvement to model predictive effectiveness is seen when implementing CFS, when compared to all features, Figure 7. Second, further improvements in generating predictive models by CFS can be realized by eliminating some of the less predictive features. Finally, reducing feature set sizes by using only the most predictive features can result in models with nearly equal effectiveness, but the resulting features subsets tend to be increasingly distinct. Supplementary files contain the distinct feature sets and subsets for dataset_2431 _and dataset_579 _for the cross validations by CFS with feature filtering by *t*-test (tr_2431_CFSfilter.tar.gz and tr_579_CFSfilter.tar.gz).

All features across all training and testing sets were itemized, for dataset_2431 _and dataset_579_, by CFS at the maximally predictive *t*-test value of 1. Totaling these features for dataset_2431 _and dataset_579 _resulted in 1097 and 897 features being used in all training and testing models, respectively. Formally these feature sets can be referred to as *S*_2431 _and *S*_579_, where *S*_2431_⊂ *S*_*all*_, *S*_579_⊂ *S*_*all*_, |*S*_2431_*| *= 1097, |*S*_579_|= 897 and |*S*_*all*_| = 1566. Itemizing the features found in common from data_2431 _and dataset_579_, results in 685 features found in both feature models (|*S*_2431 _⋂ *S*_579_| = 685) and 257 features are in neither feature model (|*S*_*all *_– (*S*_2431_∪ *S*_579_)| = 257). By comparison, 412 features are found only in the dataset_2431 _model (|*S*_2431 _– *S*_579_| = 412) and 212 features are found exclusively in the dataset_579 _model (|*S*_579 _– *S*_2431_| = 212). Comparing the observed values with expected values of 627.0, 199.9, 468.0 and 267.8, in the above presented order, these feature subset congruencies reject the null hypothesis of independence among feature subsets between datasets (×^2 ^= 39.96, *df *= 3, *P *< 0.0001). This indicates that dataset_2431 _and dataset_579 _yield feature subsets by CFS more similar to each other than by chance.

## Discussion

Several previous studies have developed predictive models of RNAi activity based on various methods of statistical association of features with activity or by machine learning methods. There were several intentions of this study. The first intent was to investigate individual features and their contribution to activity in the hopes of finding novel patterns suggestive of RNAi mechanism. The second intent was to compare multiple feature mapping methods in their relative effectiveness in building machine learning models. The third intent was to combine feature-mapping methods to generate useful machine learning models. Finally, the fourth intent was to implement feature filtering and subset selection in machine learning to improve model building and then to begin to provide a set of model building and testing tools to further the research in the properties of small non coding RNA sequences. The results of this study have revealed several features associated with RNAi activity. One feature class includes the identification of novel site-specific nucleotide compositions. A second feature class further elucidates 5' versus 3' biases in guide strand thermodynamics, suggesting a 5' bias in both guide strand and target strand secondary structure. Finally several previously unknown *N*-gram or motif patterns have been identified as features associating with RNAi activity.

### *N*-grams

The negative correlation of each 3-gram to codon usage frequency and to synonymous codon usage frequency suggests that siRNA sites in coding regions that code for rare amino acids and that deviate from using high frequency codons may provide higher RNAi activity. These observations are from using human codon usage preferences. While human and mouse codon usage frequencies show similarities in their relationship [53] and the activity data are derived from human and mouse genes, a larger comparative study with multiple organisms and genome specific motif preferences would be needed to demonstrate convincing evidence of codon association with sequence preference and RNAi activity. Further examination of 4-gram and 2-gram sequences and their association with RNAi activity suggests that a reduced preference for CpG dinucleotides could be solely used to explain these sequence motifs to activity relationships. Some of the higher-order *N*-grams are then consistent with their lower-order *N*-grams, but there appears to be some higher-order effects that influence lower-order *N*-gram observations or vice versa.

### features

The two datasets examined here, dataset_2431 _and dataset_579_, are considered to be acting under the same RNAi mechanism, and consistent with this assumption predictive models built from these datasets converge to an overall common sub set of features. Identifying features that associate with a molecule's functionality allows for the development of a pharmacophore model, namely a molecular framework that carries the essential features responsible for a drug's biological activity [54]. Assuming a broad pharmacophore definition of the set of structural features responsible for that molecule's biological activity, the continued identification of structures, sequences or chemical moieties [55-61] with influence on RNAi activity will continue to enhance the pharmacophore model of small RNAs and the interference pathways. It will also assist in the rational design of artificial RNAi effectors and inhibitors to modulate biologically relevant processes. Furthermore, the continued dissection of specific events within the RNAi pathways, RNAi delivery, dicing, guide strand uptake, target strand turn-over or RISC localization could result in the identification of specific molecular properties associated with discrete events. This would allow the fine-tuning of delivered reagents to specific RNA pathways and locations or allow for avoidance of unintended effects. Some of the similarities and differences in the various RNAi pathways are becoming known as well as the similarities and differences among organisms [16,62-64]. Further development of methods that discriminate among predictive features or feature subsets will be necessary to associate the specific causality of candidate features with their molecular outcomes.

Development of minimally predictive models has several advantages. Advantages include reducing model dimensionality, improving model generalization, reducing the time of model construction and arguably the most important in the case of computational biology enhancing the ability to interpret the model by separating the least useful features from the most useful. Several previous model building efforts have focused on the reduction of the model feature set to a minimal size, enhancing the interpretability of the feature set. While feature set reduction can certainly have a positive influence on the predictability and interpretability of a model, excessive feature set reduction can result in multiple equally predictive models with distinct or minimally intersecting feature sets. Interpretation of these distinct feature sets should then not be based on differences in the underlying biological events or differences between datasets, but can simply be due to multiple nearly equal optimal regions within feature subset space.

Position specific base preferences, as well as other feature preferences for RNA interference, suggest there are some structural biases in either RISC loading or once the guide strand is within RISC, by the short RNA sequences examined here. There are alternative mechanisms for loading RISC other than providing duplex 21 mer with 2 base 3' overhangs to cells. Alternative mechanisms for loading RISC would retain the need for any biases once loaded into RISC. However, the alternative-loading pathway might have different requirements for effective RISC loading, assuming only 2 discrete steps. Comparison between the features that allow predictive effectiveness of RNAi for alternative RISC mechanisms loading mechanisms may allow both consensus rules to establish which features are shared in common as well as the mechanism specific features for providing effective knockdown. An obvious example of siRNA's that share much of their pathway but differ in RISC loading would be to compare 21 mer siRNA sequences to dicer-substrate siRNA [65,66] sequences. Additional experiments that investigate individual events or end points will be necessary to build more realistic predictive models of the entire RNAi pathway as well as for additional organisms.

### target secondary structure

RNAi activity appears to be influenced by the structural stability of the target RNA, with the most influential sites being nearest the binding site of the guide strand's 5' end. Guide strand interaction with target strand is thought to require some minimal amount of base pairing in order to recognize a site within the target strand as effective. The precise degree of base pairing is not well established, but some sites within the guide strand are more influential than others. The seed region, positions 2 through 9 of the guide strand, are thought to provide a large contribution for guide strand to target strand interaction without complete complementarity [67]. This suggests that target accessibility for the seed region might be a primary determinant of RNA interference activity, perhaps limiting the number of target molecules that are able to initiate RNAi guide strand base pairing. Target site structure could then modulate off-target effects as well as the target sequence specific knockdown. Despite the statistically significant association of target strand secondary structure to RNAi activity within dataset_2431 _and the ability of this feature alone to produce predictive models (Figure 3, Figure 4, Table 9), the addition of target strand structure features to models that already contain other predictors of activity do not substantially improve most predictive models (Table 27). We are left with two seemingly contradictory conclusions: i) secondary structure influences RNAi activity and ii) including secondary structure in overall predictive models is not necessary if other feature classes are included. Reconciling these ideas requires additional data, but one possible explanation is that the RNAi activity models may be dominated by one or a few steps of the RNAi activity pathway, namely RISC loading, and these features dominate the signal within the present data.

Furthermore, position specific contributions to guide strand and target strand secondary structure, namely the occurrence of a position being within a Watson-Crick pair, have not been shown to have an overall association with predictive modes, but see Patzel for a case where this is observed in an engineered guide strand structure [31]. By contrast, Patzel *et al*. saw a reduction in guide strand efficacy if either the 5' or 3' end were involved in a secondary structure. However, the present observations across a population of guide strands shows a trend where positions closer the 5' end tend to have more negative influence on activity if it is involved in a secondary structure. For example, the 5' most positions from within the guide strand have the large and negative correlation between sites being within a Watson-Crick pairing event and RNAi activity, with RNAi molecules with sites within pairing events having overall lower potency (Figure 2). A similar trend for a site-specific dependency on target strand structure is seen for the overall target RNA sequence. This trend would appear where the site that is predicted to interact with the 5' most base of the guide strand is within a Watson-Crick pair within the target strand and this is associated with lower predicted RNAi activity. There is also a rough positive correlation between base-pairing occurring within the guide strand or within the target strand. This rough positive correlation occurs despite the folding of the guide strand only accounting for interactions between the 21 bases of the guide strand and the folding of the target strand only accounting for interactions between any sites within the target strand. The observations from both guide and target strand suggest some increased importance of the 5' end of the guide strand or its complement in the target strand, when compared to the 3' end.

### V. comparisons with previous machine learning models for RNAi activity

Several studies have utilized machine-learning methods to develop predictive models given siRNA sequences. Sætrom *et al*. [68] compared a combination of genetic programming and boosting algorithms (GPboost) to develop a string grammar method for learning the differences between 2 classes of sequences, effective and ineffective RNAi. GPboost was compared to SVM based classifiers that used 3 separate feature mapping methods. The results suggested that boosted Genetic Programming produced models with an *R *= 0.46 on the entire dataset (similar to the dataset_579 _used here), *R *= 0.33 in 10-fold cross validation was very effective at classifying effective versus ineffective RNAi when compared to SVM classifiers where the most accurate mapping methods resulted in *R *= 0.30. Both SVM methods were *N*-gram based, with the first being where *N *was length one through 2 and the second where *N *= 4. Care should be used in comparing model correlation values between classification and regression approaches.

Teramoto *et al*. [28] used SVM classification to discriminate between 53 effective and 41 ineffective siRNA sequences with an *N*-gram based feature method and the 3-gram and 1 through 3 grams were most effective, resulting in 87.2% and 86.2% accuracy, respectively. Furthermore, in Leave One Out Cross Validation (LOOCV) there was a correlation *R *= 0.78 between SVM scores developed under the entire dataset versus under LOOCV, but correlations between predictive model and empirical knockdown for the entire RNAi dataset or under LOOCV were not reported.

Huesken *et al*. [23] used an 84 features position specific nucleotide composition to train an artificial neural network (ANN) on dataset_2431 _to build an activity predictor that correlates predicted to observed activities on a continuously distributed dataset to a correlation of *R *= 0.66 on the entire dataset and *R *= 0.66 on a single cross validation.

Shabalina *et al*. [24] used 18 feature parameters including position specific base composition, free energies and dinucleotides to build an ANN with correlation of predicted value within the dataset (similar to the dataset_579 _used here), *R *= 0.522, but selecting the most predictive 4 features improved the correlation, *R *= 0.548, reducing model complexity. Furthermore, using just 3 of the 4 feature parameters, an ANN predicted the data from dataset_2431 _to *R *= 0.75 for model cross-validation.

Vert *et al*. [69] used position specific base composition and *N*-Grams of length 1 through 3 to produce predictive linear model from dataset_2431 _to *R *= 0.67 by cross validation. Individual features relative contributions to the resulting models were able to be evaluated in the linear models, as well as compatibility of the modeling procedure between distinct datasets, with training on dataset_2431 _and testing on a dataset of 19 mers with a size of 653 (similar to dataset_579 _used here) resulted in a model effectiveness of *R *= 0.48. Target site accessibility was also examined for 20 sequences with the largest differences in predicted and observed activities. Some of the discrepancies in predicted activities were attributed to target secondary structures, with particular influence being noted at the site of the 5' end of the guide strand target region.

Ladunga [70] developed and compared several regression SVM models from a potential pool of 572 features of position specific base composition and thermodynamics and 2252 siRNA sequences from the dataset_2431_. Model accuracy rates were 92.3% (as defined by 100 minus the average predictive difference between predicted and observed) with the polynomial kernel and weight-based feature elimination resulting in a final model with 142 features. An accuracy of 92.3% would correspond to an average error of 0.077 then a *MSE *= 0.0059. Model correlations between predicted and observed activities were not reported. Also, when feature set sub sampling was occurring by various methods, single features sets are reported, suggesting that cross validation correlations may not be precisely comparable if the feature selection did not occur within the cross validation.

The methods presented here can result in predictive models, specifically summarized from 10-fold cross validation results on dataset_2431_. First, simply applying an 84 feature position specific base composition method (method 1) can result in an SVM RBF kernel model with *R *= 0.711. Second, filtering these features can result in slight improvements to the model, with an average of 64.2 features and *R *= 0.712. Third, combining and filtering features can result in further model improvements, with an average of 500.9 features filtered from a starting feature set size of 1566 and a *R *= 0.728. Fourth, implementing a CFS method for feature selection and using only significant features at *t*-test of 1 or greater, can result in model improvements with an average of 770 features selected from a starting feature set of 1566 and *R *= 0.777. Finally, maximally predictive within dataset_2431_, but perhaps less applicable to other datasets, 1444 features from methods 1 and 11 combined can result in an average predictive model with *R *= 0.784.

## Conclusion

Here we show several feature mapping methods that reveal features that have associations with RNAi activity. Each of the mapping methods are able to produce, at least somewhat, predictive models by either cross validation or alternatively training and testing between datasets. Many of these features imply biological constraints on the RNAi mechanism previously not studied. For example, position specific base composition tends to be highly localized within the guide strand region of the target RNA but compositional biases exist outside the guide strand region. Additional patterns reveal themselves in the presence or absence of specific short motifs (*N*-grams) associating with activity. Overall stability and position specific base pairing of the secondary structures of the guide strand as well as the target strand also contain predictive features in determining RNAi activity. Secondary structures of the target strand that hold the 5' most position where the guide strand would pair in an open structure are predicted to provide more favorable knockdown than structures where this position is within an energetically stable secondary structure. However, both datasets do not show equal correlates to this structure feature and further validation of features contributing to RNAi activity may yet need more data to further resolve the specific knockdown mechanism. Furthermore, these target sequences and expression knockdown data are from mouse and human genes and cell lines.

Suggestive of the relative importance in the RNAi mechanism, the rank order of features that best model RNAi activity by SVM regression are:

1. position specific base composition

2. guide strand thermodynamics

3. N-grams 2–5

4. guide strand secondary structure features Xue et al. [33]

5. guide strand secondary structure

6. target strand secondary structure

Combining feature mapping methods together resulted in SVM regression kernels that can produce effective predictive models using large numbers of features. For example, with 1566 features and 10-fold cross validation in dataset_2431 _yields models with *R *= 0.710 and *MSE *= 0.027 and dataset_579 _yields models with *R *= 0.613 and *MSE *= 0.076. Furthermore, combining CFS and filtering features can improve model performance and reduce the number of features being considered in model building, at *t*-test of 1, dataset_2431 _yields models with 769 features, *R *= 0.777 and *MSE *= 0.022 and dataset_579 _yields models with 542 features, *R *= 0.622 and *MSE *= 0.072. Predictive SVM models are able to be produced from individual or combinations of features, and methods such as feature filtering or CFS can improve model performance. However, minimizing feature sets sizes can result in distinct sub sets of features being selected with nearly equal model performance among feature subsets.

## Availability and requirements

Project name: SEQ2SVM;

Project download: ;

Operating system(s): GNU compliant, Linux tested;

Programming language: C/C++;

License: GNU GPL;

Any restrictions to use by non-academics: none.

## Supplementary Material

Additional file 1 suppl1_comparison_position_specific_base_composition. Sites and bases within the guide strand found from several studies and datasets to be either significant or not significant in their influence of RNAi activity. Click here for file

Additional file 2 suppl2_all_features_corr_descr_tval. Features with their associated descriptions, correlations with RNAi activity and t-test values of significance. Click here for file

Additional file 3 supplementary_figure_1. The base composition bias within the localized target site of the siRNA guide strand, for 100 bases upstream and downstream of the guide strand target area. Click here for file

Additional file 4 supplementary_figure_2. The base composition bias within the localized target site of the siRNA guide strand, for 21 bases upstream and downstream of the guide strand target area. Click here for file

Additional file 5 tr_2431_cfsfilters. The features found to be useful by Correlation based Feature in training and testing the 2431 dataset by cross validation, at t-test values from 0 to 90. Click here for file

Additional file 6 tr_579_cfsfilters. The features found to be useful by Correlation based Feature in training and testing the 579 dataset by cross validation, at t-test values from 0 to 90. Click here for file

Additional file 7 seq2svm_0.3. An GNU platform deployable GPL code base for performing SVM modeling on small RNA sequences, with examples. Deploy by unzipping, untarring, and building with configure and make. See the included readme files. Updated versions will be available at . Click here for file

## References

[B1] Fire A, Xu SQ, Montgomery MK, Kostas SA, Driver SE, Mello CC (1998). Potent and specific genetic interference by double-stranded RNA in Caenorhabditis elegans. Nature.

[B2] Matzke MA, Birchler JA (2005). RNAi-mediated pathways in the nucleus. Nat Rev Genet.

[B3] Kawasaki H, Taira K (2005). Transcriptional gene silencing by short interfering RNAs. Curr Opin Mol Ther.

[B4] Weinberg MS, Villeneuve LM, Ehsani A, Amarzguioui M, Aagaard L, Chen ZX, Riggs AD, Rossi JJ, Morris KV (2006). The antisense strand of small interfering RNAs directs histone methylation and transcriptional gene silencing in human cells. Rna.

[B5] Filipowicz W, Jaskiewicz L, Kolb FA, Pillai RS (2005). Post-transcriptional gene silencing by siRNAs and miRNAs. Curr Opin Struct Biol.

[B6] Tomari Y, Zamore PD (2005). Perspective: machines for RNAi. Genes Dev.

[B7] Hannon GJ, Rossi JJ (2004). Unlocking the potential of the human genome with RNA interference. Nature.

[B8] Li LC, Okino ST, Zhao H, Pookot D, Place RF, Urakami S, Enokida H, Dahiya R (2006). Small dsRNAs incude transcriptional activation in human cells. Proc Natl Acad Sci U S A.

[B9] Schwarz DS, Hutvagner G, Du T, Xu Z, Aronin N, Zamore PD (2003). Asymmetry in the Assembly of the RNAi Enzyme Complex. Cell.

[B10] Khvorova A, Reynolds A, Jayasena SD (2003). Functional siRNAs and miRNAs exhibit strand bias. Cell.

[B11] Ui-Tei K, Naito Y, Takahashi F, Haraguchi T, Ohki-Hamazaki H, Juni A, Ueda R, Saigo K (2004). Guidelines for the selection of highly effective siRNA sequences for mammalian and chick RNA interference. Nucleic Acids Res.

[B12] Amarzguioui M, Prydz H (2004). An algorithm for selection of functional siRNA sequences. Biochemical and Biophysical Research Communications.

[B13] Hsieh AC, Bo R, Manola J, Vazquez F, Bare O, Khvorova A, Scaringe S, Sellers WR (2004). A library of siRNA duplexes targeting the phosphoinositide 3-kinase pathway: determinants of gene silencing for use in cell-based screens. Nucleic Acids Res.

[B14] Reynolds A, Leake D, Boese Q, Scaringe S, Marshall WS, Khvorova A (2004). Rational siRNA design for RNA interference. Nat Biotechnol.

[B15] Ying SY, Chang DC, Miller JD, Lin SL (2006). The microRNA: overview of the RNA gene that modulates gene functions. Methods Mol Biol.

[B16] Hall TM (2005). Structure and function of argonaute proteins. Structure.

[B17] Kerschen A, Napoli CA, Jorgensen RA, Muller AE (2004). Effectiveness of RNA interference in transgenic plants. FEBS Lett.

[B18] Walters DK, Jelinek DF (2002). The effectiveness of double-stranded short inhibitory RNAs (siRNAs) may depend on the method of transfection. Antisense Nucleic Acid Drug Dev.

[B19] Sontheimer EJ (2005). Assembly and function of RNA silencing complexes. Nat Rev Mol Cell Biol.

[B20] Takasaki S, Kotani S, Konagaya A (2004). An Effective Method for Selecting siRNA Target Sequences in Mammalian Cells. Cell Cycle.

[B21] Luo KQ, Chang DC (2004). The gene-silencing efficiency of siRNA is strongly dependent on the local structure of mRNA at the targeted region. Biochem Biophys Res Commun.

[B22] Ge G, Wong GW, Luo B (2005). Prediction of siRNA knockdown efficacy using artificial neural network models. Biochem Biophys Res Commun.

[B23] Huesken D, Lange J, Mickanin C, Weiler J, Asselbergs F, Warner J, Meloon B, Engel S, Rosenberg A, Cohen D, Labow M, Reinhardt M, Natt F, Hall J (2005). Design of a genome-wide siRNA library using an artificial neural network. Nat Biotechnol.

[B24] Shabalina SA, Spiridonov AN, Ogurtsov AY (2006). Computational models with thermodynamic and composition features improve siRNA design. BMC Bioinformatics.

[B25] Sætrom P (2004). Predicting the efficacy of short oligonucleotides in antisense and RNAi experiments with boosted genetic programming. Bioinformatics.

[B26] Chalk AM, Wahlestedt C, Sonnhammer EL (2004). Improved and automated prediction of effective siRNA. Biochem Biophys Res Commun.

[B27] Jagla B, Aulner N, Kelly PD, Song D, Volchuk A, Zatorski A, Shum D, Mayer T, De Angelis DA, Ouerfelli O, Rutishauser U, Rothman JE (2005). Sequence characteristics of functional siRNAs. RNA.

[B28] Teramoto R, Aoki M, Kimura T, Kanaoka M (2005). Prediction of siRNA functionality using generalized string kernel and support vector machine. FEBS Lett.

[B29] Jia P, Shi T, Cai Y, Li Y (2006). Demonstration of two novel methods for predicting functional siRNA efficiency. BMC Bioinformatics.

[B30] Du Q, Thonberg H, Wang J, Wahlestedt C, Liang Z (2005). A systematic analysis of the silencing effects of an active siRNA at all single-nucleotide mismatched target sites. Nucleic Acids Res.

[B31] Patzel V, Rutz S, Dietrich I, Koberle C, Scheffold A, Kaufmann SH (2005). Design of siRNAs producing unstructured guide-RNAs results in improved RNA interference efficiency. Nat Biotechnol.

[B32] Xue C, Li F, He T, Liu GP, Li Y, Zhang X (2005). Classification of real and pseudo microRNA precursors using local structure-sequence features and support vector machine. BMC Bioinformatics.

[B33] Bohula EA SAJ (2003). The efficacy of small interfering RNAs targeted to the type 1 insulin-like growth factor receptor (IGF1R) is influenced by secondary structure in the IGF1R transcript. J Biol Chemistry.

[B34] Vickers TA, Koo S, Bennett CF, Crooke ST, Dean NM, Baker BF (2003). Efficient reduction of target RNAs by small interfering RNA and RNase H-dependent antisense agents. A comparative analysis. J Biol Chem.

[B35] Kretschmer-Kazemi Far R, Sczakiel G (2003). The activity of siRNA in mammalian cells is related to structural target accessibility: a comparison with antisense oligonucleotides. Nucleic Acids Res.

[B36] Yoshinari K, Miyagishi M, Taira K (2004). Effects on RNAi of the tight structure, sequence and position of the targeted region. Nucleic Acids Res.

[B37] Heale BS, Soifer HS, Bowers C, Rossi JJ (2005). siRNA target site secondary structure predictions using local stable substructures. Nucleic Acids Res.

[B38] Schubert S, Grunweller A, Erdmann VA, Kurreck J (2005). Local RNA target structure influences siRNA efficacy: systematic analysis of intentionally designed binding regions. J Mol Biol.

[B39] Overhoff M, Alken M, Far RK, Lemaitre M, Lebleu B, Sczakiel G, Robbins I (2005). Local RNA target structure influences siRNA efficacy: a systematic global analysis. J Mol Biol.

[B40] Brown KM, Chu CY, Rana TM (2005). Target accessibility dictates the potency of human RISC. Nat Struct Mol Biol.

[B41] Vapnik V (1998). Statistical Learning Theory.

[B42] Joachims T (2002). Learning to classify test using support vector machines: methods theory and algorithms.

[B43] Haasdonk B (2005). Feature space interpretation of SVMs with indefinite kernels. IEEE Trans Pattern Anal Mach Intell.

[B44] Huesken D, Lange J, Mickanin C, Weiler J, Asselbergs F, Warner J, Meloon B, Engel S, Rosenberg A, Cohen D, Labow M, Reinhardt M, Natt F, Hall J (2005). Corrigendum: Design of a genome-wide siRNA library using an artificial neural network. Nat Biotechnol.

[B45] NCBI. http://www.ncbi.nlm.nih.gov/.

[B46] Xia T, SantaLucia JJ, Burkard ME, Kierzek R, Schroeder SJ, Jiao X, Cox C, Turner DH (1998). Thermodynamic parameters for an extended nearest-neighbor model for formation of RNA duplexes with Watson-Crick base pairs. Biochemistry.

[B47] Shannon CE (1948). A mathematical theory of communication. Bell System Technical Journal.

[B48] Hofacker IL (2003). Vienna RNA secondary structure server. Nucleic Acids Res.

[B49] Chang CC, Lin CJ (2001). Training nu-support vector classifiers: theory and algorithms. Neural Comput.

[B50] Hall M (1999). Correlation-based Feature Selection for Machine Learning. Department of Computer Science.

[B51] SEQ2SVM. ftp://scitoolsftp.idtdna.com/SEQ2SVM/.

[B52] Caiafa P, Zampieri M (2005). DNA methylation and chromatin structure: the puzzling CpG islands. J Cell Biochem.

[B53] Jorgensen FG, Hobolth A, Hornshoj H, Bendixen C, Fredholm M, Schierup MH (2005). Comparative analysis of protein coding sequences from human, mouse and the domesticated pig. BMC Biol.

[B54] Gund P, Hahn FE (1977). Three-dimensional pharmacophoric pattern searching. Progress in Molecular and Subcellular Biology.

[B55] Amarzguioui M, Holen T, Babaie E, Prydz H (2003). Tolerance for mutations and chemical modifications in a siRNA. Nucleic Acids Research.

[B56] Chiu YL, Rana TM (2003). siRNA function in RNAi: a chemical modification analysis. RNA.

[B57] Harborth J, Elbashir SM, Vandenburgh K, Manninga H, Scaringe SA, Weber K, Tuschl T (2003). Sequence, chemical, and structural variation of small interfering RNAs and short hairpin RNAs and the effect on mammalian gene silencing. Antisense Nucleic Acid Drug Dev.

[B58] Li ZY, Mao H, Kallick DA, Gorenstein DG (2005). The effects of thiophosphate substitutions on native siRNA gene silencing. Biochem Biophys Res Commun.

[B59] Hoshika S, Minakawa N, Kamiya H, Harashima H, Matsuda A (2005). RNA interference induced by siRNAs modified with 4'-thioribonucleosides in cultured mammalian cells. FEBS Lett.

[B60] Dowler T, Bergeron D, Tedeschi AL, Paquet L, Ferrari N, Damha MJ (2006). Improvements in siRNA properties mediated by 2'-deoxy-2'-fluoro-beta-D-arabinonucleic acid (FANA). Nucleic Acids Res.

[B61] Zhang HY, Du Q, Wahlestedt C, Liang Z (2006). RNA Interference with chemically modified siRNA. Curr Top Med Chem.

[B62] Collins RE, Cheng X (2006). Structural and biochemical advances in mammalian RNAi. J Cell Biochem.

[B63] Saumet A, Lecellier CH (2006). Anti-viral RNA silencing: do we look like plants?. Retrovirology.

[B64] Pham JW, Sontheimer EJ (2005). Molecular requirements for RNA-induced silencing complex assembly in the Drosophila RNA interference pathway. J Biol Chem.

[B65] Kim DH, Behlke MA, Rose SD, Chang MS, Choi S, Rossi JJ (2005). Synthetic dsRNA Dicer substrates enhance RNAi potency and efficacy. Nat Biotechnol.

[B66] Rose SD, Kim DH, Amarzguioui M, Heidel JD, Collingwood MA, Davis ME, Rossi JJ, Behlke MA (2005). Functional polarity is introduced by Dicer processing of short substrate RNAs. Nucleic Acids Res.

[B67] Birmingham A, Anderson EM, Reynolds A, Ilsley-Tyree D, Leake D, Fedorov Y, Baskerville S, Maksimova E, Robinson K, Karpilow J, Marshall WS, Khvorova A (2006). 3' UTR seed matches, but not overall identity, are associated with RNAi off-targets. Nat Methods.

[B68] Sætrom P, Snove O (2004). A comparison of siRNA efficacy predictors. Biochem Biophys Res Commun.

[B69] Vert JP, Foveau N, Lajaunie C, Vandenbrouck Y (2006). An accurate and interpretable model for siRNA efficacy prediction. BMC Bioinformatics.

[B70] Ladunga I (2007). More complete gene silencing by fewer siRNAs: transparent optimized design and biophysical signature. Nucleic Acids Res.

