# Supplementary figures and images for: Improving model predictions for RNA interference activities that use support vector machine regression by combining and filtering features
Source: BMC Bioinformatics. 2007 Jun 6;8:182. doi: 10.1186/1471-2105-8-182 (PMC1906837; doi:10.1186/1471-2105-8-182)

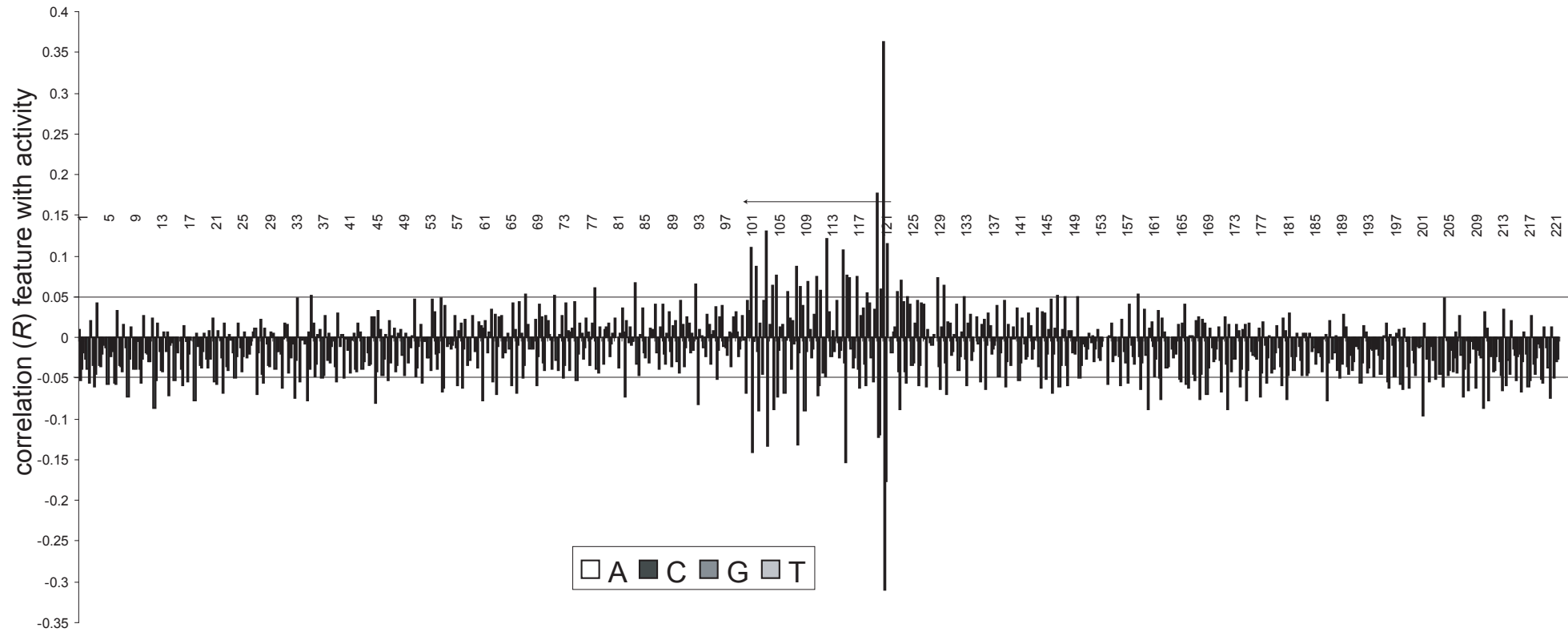

Supplement: Additional file 3 — supplementary_figure_1. The base composition bias within the localized target site of the siRNA guide strand, for 100 bases upstream and downstream of the guide strand target area. [file 1471-2105-8-182-S3.pdf]

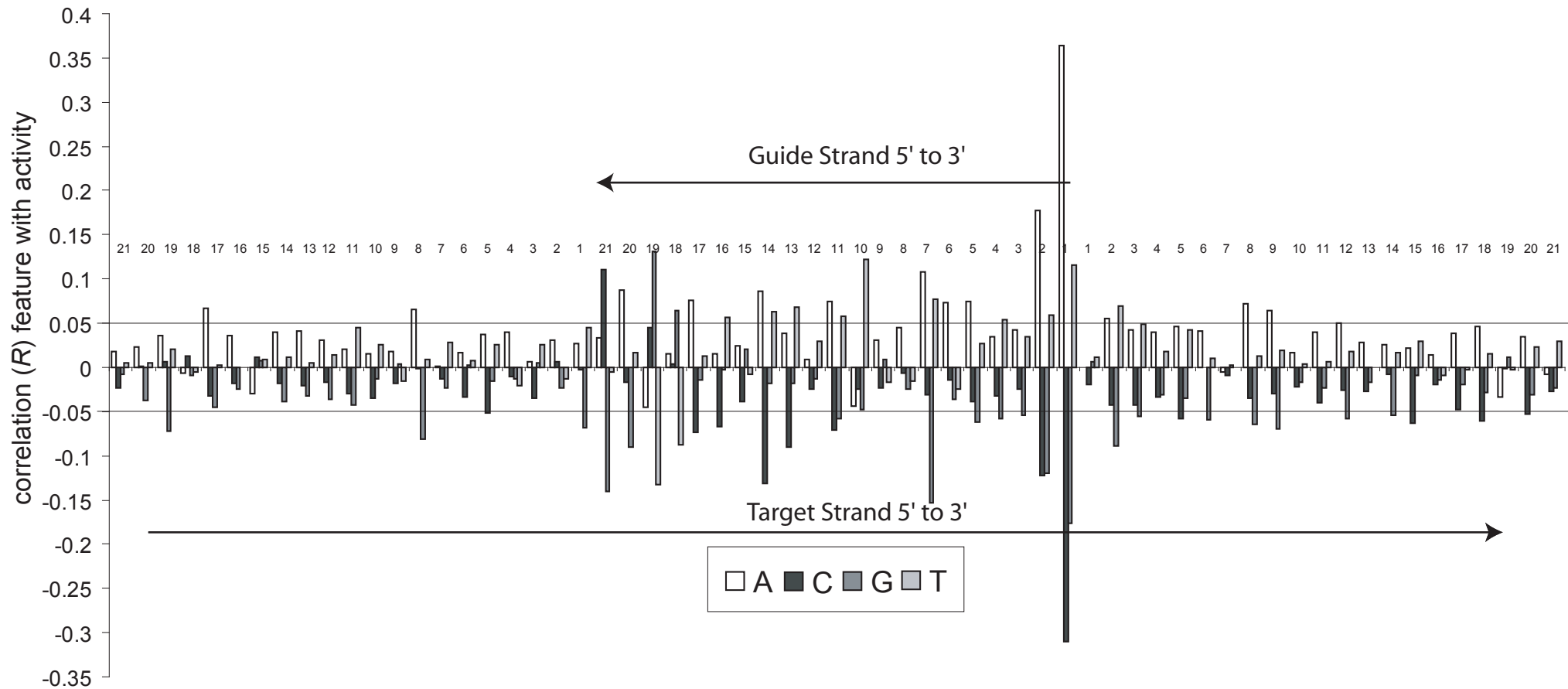

Supplement: Additional file 4 — supplementary_figure_2. The base composition bias within the localized target site of the siRNA guide strand, for 21 bases upstream and downstream of the guide strand target area. [file 1471-2105-8-182-S4.pdf]
