# Supplementary material for: Improving model predictions for RNA interference activities that use support vector machine regression by combining and filtering features
Source: BMC Bioinformatics. 2007 Jun 6;8:182. doi: 10.1186/1471-2105-8-182 (PMC1906837; doi:10.1186/1471-2105-8-182)
Supplement: Additional file 7 — seq2svm_0.3. An GNU platform deployable GPL code base for performing SVM modeling on small RNA sequences, with examples. Deploy by unzipping, untarring, and building with configure and make. See the included readme files. Updated versions will be available at . [file 1471-2105-8-182-S7.gz › seq2svm/src/libsvm-2.71/FAQ.html]

LIBSVM FAQ

**# LIBSVM FAQ**
**last modified :** 
Sun, 24 Oct 2004 01:42:17 GMT
- All Questions(48)

**- Q1:\_Some\_courses\_which\_have\_used\_libsvm\_as\_a\_tool(1)
- Q2:\_Installation\_and\_running\_the\_program(8)
- Q3:\_Data\_preparation(3)
- Q4:\_Training\_and\_prediction(25)
- Q5:\_Graphic\_Interface(3)
- Q6:\_Java\_version\_of\_libsvm(4)
- Q7:\_Python\_Interface(4)**


- Some courses which have used libsvm as a tool
- Where can I find documents of libsvm ?
- What are changes in previous versions?
- I would like to cite libsvm. Which paper should I cite ?
- I would like to use libsvm in my software. Is there any license problem?
- Is there a repository of additional tools based on libsvm?
- On unix machines, I got "error in loading shared libraries" or "cannot open shared object file." What happened ?
- I have modified the source and would like to build the graphic interface "svm-toy" on MS windows. How should I do it ?
- I am an MS windows user but why only one (SVM\_toy) of those precompiled .exe actually runs ?
- Why sometimes not all attributes of a data appear in the training/model files ?
- What if my data are non-numerical ?
- Why do you consider sparse format ? Will the training of dense data be much slower ?
- The output of training C-SVM is like the following. What do they mean ?
- Can you explain more about the model file ?
- Should I use float or double to store numbers in the cache ?
- How do I choose the kernel ?
- Does libsvm have special treatments for linear SVM ?
- The number of free support vectors is large. What should I do ?
- Should I scale training and testing data in a similar way ?
- Does it make a big difference if I scale each attribute to [0,1] instead of [-1,1] ?
- The prediction rate is low. How could I improve it ?
- My data are unbalanced. Could libsvm handle such problems ?
- What is the difference between nu-SVC and C-SVC ?
- The program keeps running without showing any output. What should I do ?
- The program keeps running (with output, i.e. many dots). What should I do ?
- The training time is too long. What should I do ?
- How do I get the decision value(s) ?
- For some problem sets if I use a large cache (i.e. large -m) on a linux machine, why sometimes I get "segmentation fault ?"
- How do I disable screen output of svm-train and svm-predict ?
- I would like to use my own kernel but find out that there are two subroutines for kernel evaluations: k\_function() and kernel\_function(). Which one should I modify ?
- What method does libsvm use for multi-class SVM ? Why don't you use the "1-against-the rest" method ?
- After doing cross validation, why there is no model file outputted ?
- I would like to try different random partition for cross validation, how could I do it ?
- I would like to solve L2-SVM (i.e., error term is quadratic). How should I modify the code ?
- There seems to be a zero division ?
- How do I choose parameters for one-class svm as training data are in only one class ?
- Why training a probability model (i.e., -b 1) takes longer time ?
- How can I save images drawn by svm-toy?
- I press the "load" button to load data points but why svm-toy does not draw them ?
- I would like svm-toy to handle more than three classes of data, what should I do ?
- What is the difference between Java version and C++ version of libsvm?
- Is the Java version significantly slower than the C++ version?
- While training I get the following error message: java.lang.OutOfMemoryError. What is wrong?
- Why you have the main source file svm.m4 and then transform it to svm.java?
- Using python on MS windows, it fails to load the dll file.
- How to modify the python interface on MS windows and rebuild the dll file ?
- Except the python-C++ interface provided, could I use Jython to call libsvm ?
- How could I install the python interface on Mac OS?

---

**Q: Some courses which have used libsvm as a tool**
  

- Institute for Computer Science,
  Faculty of Applied Science, University of Freiburg, Germany
  - Division of Mathematics and Computer Science.
    Faculteit der Exacte Wetenschappen
    Vrije Universiteit, The Netherlands. - Electrical and Computer Engineering Department,
      University of Wisconsin-Madison
      - Technion (Israel Institute of Technology), Israel.- Computer and Information Sciences Dept., University of Florida- The Institute of Computer Science,
            University of Nairobi, Kenya.- Applied Mathematics and Computer Science, University of Iceland.

[Go Top]


---


**Q: Where can I find documents of libsvm ?**
  

In the package there is a README file which
details all options, data format, and library calls.
The model selection tool and the python interface
have a separate README under the directory python.
The guide
A practical guide to support vector classification
 shows beginners how to train/test their data.
The paper LIBSVM
: a library for support vector machines discusses the implementation of
libsvm in detail.

[Go Top]


---


**Q: What are changes in previous versions?**
  

See the change log.

[Go Top]


---


**Q: I would like to cite libsvm. Which paper should I cite ?** 
  

Please cite the following document:

Chih-Chung Chang and Chih-Jen Lin, LIBSVM
: a library for support vector machines, 2001.
Software available at http://www.csie.ntu.edu.tw/~cjlin/libsvm

The bibtex format is as follows

```
@Manual{CC01a,
  author =	 {Chih-Chung Chang and Chih-Jen Lin},
  title =	 {{LIBSVM}: a library for support vector machines},
  year =	 {2001},
  note =	 {Software available at {\tt http://www.csie.ntu.edu.tw/\verb"~"cjlin/libsvm}},
}
```

[Go Top]


---


**Q: I would like to use libsvm in my software. Is there any license problem?**
  

The libsvm license ("the modified BSD license")
is compatible with many
free software licenses such as GPL. Hence, it is very easy to
use libsvm in your software.
It can also be used in commercial products.

[Go Top]


---


**Q: Is there a repository of additional tools based on libsvm?**
  

Yes, see libsvm
tools

[Go Top]


---


**Q: On unix machines, I got "error in loading shared libraries" or "cannot open shared object file." What happened ?** 
  

This usually happens if you compile the code
on one machine and run it on another which has incompatible
libraries.
Try to recompile the program on that machine or use static linking.

[Go Top]


---


**Q: I have modified the source and would like to build the graphic interface "svm-toy" on MS windows. How should I do it ?**
  

Build it as a project by choosing "Win32 Application."
On the other hand, for "svm-train" and "svm-predict"
you want to choose "Win32 Console Application."
After libsvm 2.5, you can also use the file Makefile.win.
See details in README.

If you are not using Makefile.win and see the following
link error

```
LIBCMTD.lib(wwincrt0.obj) : error LNK2001: unresolved external symbol
_wWinMain@16
```

you may have selected a wrong project type.

[Go Top]


---


**Q: I am an MS windows user but why only one (SVM\_toy) of those precompiled .exe actually runs ?** 
  

You need to open a command window
and type svmtrain.exe to see all options.
Some examples are in README file.

[Go Top]


---


**Q: Why sometimes not all attributes of a data appear in the training/model files ?**
  

libsvm uses the so called "sparse" format where zero
values do not need to be stored. Hence a data with attributes

```
1 0 2 0
```

is represented as

```
1:1 3:2
```

[Go Top]


---


**Q: What if my data are non-numerical ?**
  

Currently libsvm supports only numerical data.
You may have to change non-numerical data to
numerical. For example, you can use several
binary attributes to represent a categorical
attribute.

[Go Top]


---


**Q: Why do you consider sparse format ? Will the training of dense data be much slower ?**
  

This is a controversial issue. The kernel
evaluation (i.e. inner product) of sparse vectors is slower
so the total training time can be at least twice or three times
of that using the dense format.
However, we cannot support only dense format as then we CANNOT
handle extremely sparse cases. Simplicity of the code is another
concern. Right now we decide to support
the sparse format only.

[Go Top]


---


**Q: The output of training C-SVM is like the following. What do they mean ?**
  
  
optimization finished, #iter = 219
  
nu = 0.431030
  
obj = -100.877286, rho = 0.424632
  
nSV = 132, nBSV = 107
  
Total nSV = 132

obj is the optimal objective value of the dual SVM problem.
rho is the bias term in the decision function
sgn(w^Tx - rho).
nSV and nBSV are number of support vectors and bounded support
vectors (i.e., alpha\_i = C). nu-svm is a somewhat equivalent
form of C-SVM where C is replaced by nu. nu simply shows the
corresponding parameter. More details are in
libsvm document.

[Go Top]


---


**Q: Can you explain more about the model file ?**
