# Supplementary material for: Improving model predictions for RNA interference activities that use support vector machine regression by combining and filtering features
Source: BMC Bioinformatics. 2007 Jun 6;8:182. doi: 10.1186/1471-2105-8-182 (PMC1906837; doi:10.1186/1471-2105-8-182)
Supplement: Additional file 7 — seq2svm_0.3. An GNU platform deployable GPL code base for performing SVM modeling on small RNA sequences, with examples. Deploy by unzipping, untarring, and building with configure and make. See the included readme files. Updated versions will be available at . [file 1471-2105-8-182-S7.gz › seq2svm/src/libRNAfold-2.4/ViennaRNA-1.4/man/RNAlib.html]

Vienna RNA Package


# Vienna RNA Package

---

# Introduction

The core of the Vienna RNA Package is formed by a collection of routines
for the prediction and comparison of RNA secondary structures. These
routines can be accessed through stand-alone programs, such as RNAfold,
RNAdistance etc., which should be sufficient for most users. For those who
wish to develop their own programs we provide a library which can be linked
to your own code.

This document only describes the library and will be primarily useful to
programmers. The stand-alone programs are described in separate man
pages. The latest version of the package including source code and html
versions of the documentation can be found at
the ViennaRNA page.
This manual documents version 1.3.

Please send comments and bug reports to
<Ivo.Hofacker@tbi.univie.ac.at>.

# Folding Routines

## Minimum free Energy Folding

The library provides a fast dynamic programming minimum free energy
folding algorithm as described by Zuker & Stiegler (1981).
Associated functions are:

Function: float **fold** *(char\* sequence, char\* structure)*: folds the sequence and returns the minimum free energy in kcal/mol; the mfe structure in bracket notation (see section Representations of Secondary Structures) is returned in structure. Sufficient space for string of the same length as sequence must be allocated for structure before calling `fold()`. If `fold_constrained` (see section Global Variables for the Folding Routines) is 1, the structure string is interpreted on input as a list of constraints for the folding. The characters " | x < > " mark bases that are paired, unpaired, paired upstream, or downstream, respectively; matching brackets " ( ) " denote base pairs, dots "." are used for unconstrained bases. Constrained folding works by assigning bonus energies to all structures compliing with the constraint.

Function: float **energy\_of\_struct** *(char\* sequence, char\* structure)*: calculates the energy of sequence on the structure

Function: void **initialize\_fold** *(int length)*: allocates memory for folding sequences not longer than length; sets up pairing matrix and energy parameters. Has to be called before the first call to `fold()`.

Function: void **free\_arrays** *()*: frees the memory allocated by `initialize_fold()`.

Function: void **update\_fold\_params** *()*: call this to recalculate the pair matrix and energy parameters after a change in folding parameters like `temperature` (see section Global Variables for the Folding Routines).

Prototypes for these functions are declared in `fold.h'.

## Partition Function Folding

Instead of the minimum free energy structure the partition function of
all possible structures and from that the pairing probability for every
possible pair can be calculated, using a dynamic programming algorithm
as described by McCaskill (1990). The following
functions are provided:

Function: float **pf\_fold** *(char\* sequence, char\* structure)*: calculates the partition function *Z* of sequence and returns the free energy of the ensemble *F* in kcal/mol, where *F=-kT ln(Z)*. If structure is not a NULL pointer on input, it contains on return a string consisting of the letters " . , | { } ( ) " denoting bases that are essentially unpaired, weakly paired, strongly paired without preference, weakly upstream (downstream) paired, or strongly up- (down-)stream paired bases, respectively. If `fold_constrained` (see section Global Variables for the Folding Routines) is 1, the structure string is interpreted on input as a list of constraints for the folding. The character "x" marks bases that must be unpaired, matching brackets " ( ) " denote base pairs, all other characters are ignored. Any pairs conflicting with the constraint will be forbidden. This usually sufficient to ensure the constraints are honored. If `do_backtrack` (see section Global Variables for the Folding Routines) has been set to 0 base pairing probabilities will not be computed (saving CPU time), otherwise the `pr[iindx[i]-j]` (see section Global Variables for the Folding Routines) will contain the probability that bases i and j pair.

Function: void **init\_pf\_fold** *(int length)*: allocates memory for folding sequences not longer than length; sets up pairing matrix and energy parameters. Has to be called before the first call to `pf_fold()`.

Function: void **free\_pf\_arrays** *(void)*: frees the memory allocated by `init_pf_fold()`.

Function: void **update\_pf\_params** *(int length)*: Call this function to recalculate the pair matrix and energy parameters after a change in folding parameters like temperature (see section Global Variables for the Folding Routines).

Prototypes for these functions are declared in `part\_func.h'.

## Inverse Folding

We provide two functions that search for sequences with a given
structure, thereby inverting the folding routines.

Function: float **inverse\_fold** *(char\* start, char\* target)*: searches for a sequence with minimum free energy structure target, starting with sequence start. It returns 0 if the search was successful, otherwise a structure distance to target is returned. The found sequence is returned in start. If `give_up` is set to 1, the function will return as soon as it is clear that the search will be unsuccessful, this speeds up the algorithm if you are only interested in exact solutions. Since `inverse_fold()` calls `fold()` you have to allocate memory for folding by calling `initialize_fold()`

Function: float **inverse\_pf\_fold** *(char\* start, char\* target)*: searches for a sequence with maximum probability to fold into structure target using the partition function algorithm. It returns -kT log(p) where p is the frequency of target in the ensemble of possible structures. This is usually much slower than `inverse_fold()`. Since `inverse_pf_fold()` calls `pf_fold()` you have to allocate memory for folding by calling `init_pf_fold()`

Variable: char **\*symbolset**: The global variable `char *symbolset` points to the allowed bases, initially `"AUGC"`. It can be used to design sequences from reduced alphabets.

Prototypes for these functions are declared in `inverse.h'.

## Global Variables for the Folding Routines

The following global variables change the behavior the folding
algorithms or contain additional information after folding.

Variable: int **noGU**: do not allow GU pairs if equal 1; default is 0.

Variable: int **no\_closingGU**: if 1 allow GU pairs only inside stacks, not as closing pairs; default is 0.

Variable: int **noLonelyPairs**: Disallow all pairs which can **only** occur as lonely pairs (i.e. as helix of length 1). This avoids lonely base pairs in the predicted structures in most cases.

Variable: int **tetra\_loop**: include special stabilizing energies for some tetra loops; default is 1.

Variable: int **energy\_set**: if 1 or 2: fold sequences from an artificial alphabet ABCD..., where A pairs B, C pairs D, etc. using either GC (1) or AU parameters (2); default is 0, you probably don't want to change it.

Variable: float **temperature**: rescale energy parameters to a temperature of `temperature` C. Default is 37C. You have to call the update\_...\_params() functions after changing this parameter.

Variable: int **dangles**: if set to 0 no stabilizing energies are assigned to bases adjacent to helices in free ends and multiloops (so called dangling ends). Normally (`dangles = 1`) dangling end energies are assigned only to unpaired bases and a base cannot participate simultaneously in two dangling ends. In the partition function algorithm `pf_fold()` these checks are neglected. If `dangles` is set to 2, the `fold()` and `energy_of_struct()` function will also follow this convention. This treatment of dangling ends gives more favorable energies to helices directly adjacent to one another, which can be beneficial since such helices often do engage in stabilizing interactions through co-axial stacking. If `dangles = 3` co-axial stacking is explicitely included for adjacent helices in mutli-loops. The option affects only mfe folding and energy evaluation (`fold()` and `energy_of_struct()`), as well as suboptimal folding via re-evaluation of energies. Co-axial stacking with one intervening mismatch is not considered so far. Default is 1, `pf_fold()` treats 1 as 2.

Variable: char\* **nonstandards**: Lists additional base pairs that will be allowed to form in addition to GC, CG, AU, UA, GU and UG. Nonstandard base pairs are given a stacking energy of 0.

Variable: struct bond { int i,j;} **base\_pair**: Contains a list of base pairs after a call to `fold()`. `base_pair[0].i` contains the total number of pairs.

Variable: double\* **pr**: contains the base pair probability matrix after a call to `pf_fold()`.

Variable: int\* **iindx**: index array to move through pr. The probability for base i and j to form a pair is in `pr[iindx[i]-j]`.

Variable: float **pf\_scale**: a scaling factor used by `pf_fold()` to avoid overflows. Should be set to approximately exp*((-F/kT)/length)*, where *F* is an estimate for the ensemble free energy, for example the minimum free energy. You must call `update_pf_params()` or `init_pf_fold()` after changing this parameter. If pf\_scale is -1 (the default) , an estimate will be provided automatically when calling `init_pf_fold()` or `update_pf_params()`. The automatic estimate is usually insufficient for sequences more than a few hundred bases long.

Variable: int **fold\_constrained**: If 1, calculate constrained minimum free energy structures. See section Minimum free Energy Folding, for more information. Default is 0;

Variable: int **do\_backtrack**: if 0, do not calculate pair probabilities in `pf_fold()`; this is about twice as fast. Default is 1.

Variable: char **backtrack\_type**: only for use by `inverse_fold()`; 'C': force (1,N) to be paired, 'M' fold as if the sequence were inside a multi-loop. Otherwise the usual mfe structure is computed.

include `fold\_vars.h' if you want to change any of these variables
from their defaults.

## Energy Parameter Files

A default set of parameters, identical to the one described in Mathews
et.al. (1999), is compiled into the library.
Alternately, parameters can be read from and written to a file.

Function: void **read\_parameter\_file** *(const char fname[])*: reads energy parameters from file fname. See below for the format of the parameter file.

Function: void **write\_parameter\_file** *(const char fname[])*: writes current energy parameters to the file fname.

The following describes the file format expected by
`read_parameter_file()`. All energies should be given as integers in
units of 0.01kcal/mol.

Various loop parameters depend in general on the pairs closing the loops,
as well as unpaired bases in the loops. Internally, the library
distinguishes 8 types of pairs (CG=1, GC=2, GU=3, UG=4, AU=5, UA=6,
nonstandard=7, 0= no pair), and 5 types of bases (A=1, C=2, G=3, U=4 and 0
for anything else). Parameters belonging to pairs of type 0 are not listed
in the parameter files, but values for nonstandard pairs (type 7) and
nonstandard bases (type 0) are. Thus, a table for symmetric size 2 interior
loops would have 7\*7\*5\*5 entries (2 pairs, two unpaired bases).

The order of entries always uses the closing pair or pairs as the
first indices followed by the unpaired bases in 5' to 3' direction.
To determine the type of a pair consider the base at 5' end of each strand
first, i.e. use the pairs *(i,j)* and *(q,p)* for an interior
loop with *i<p<q<j* . This is probably better explained by an
example. Consider the symmetric size 4 interior loop

```
                      5'-GAUA-3' 
                      3'-CGCU-5'
```

the first pair is GC, the second UA (not AU!) the unpaired bases are (in 5'
to 3' direction, starting at the first pair) A U C G. Thus we need entry
[2,6,1,4,2,3] of the corresponding table. Because the loop is
symmetric you could equally well describe it by UA GC C G A U, i.e. entry
[6,2,2,3,1,4]. Be careful to preserve this symmetry when editing
parameter tables!

The first line of the file should read
  
## RNAfold parameter file

lines of the form  
# token  
mark the beginning of a list of energy parameters of the type specified by
token. The following tokens are recognized:

# stack\_energies  
The list of free energies for stacked pairs, indexed by the two closing
pairs. The list should be formated as symmetric an 7\*7 matrix,conforming
to the order explained above. As an example the stacked pair

```
                      5'-GU-3'        5'-AC-3'
                      3'-CA-5'        3'-UG-5'
```

corresponds to the entry [2,5], which should be identical to [5,2].
Note that the format has changed from previous releases, to make it
consistent with other loop parameters.

# stack\_enthalpies  
enthalpies for stacked pairs, used to rescale stacking energies to
temperatures other than 37C. Same format as stack\_energies.

# hairpin  
Free energies of hairpin loops as a function of size. The list should
contain 31 entries on one or more lines. Since the minimum size of a
hairpin loop is 3 and we start counting with 0, the first three values
should be INF to indicate a forbidden value.

# bulge  
Free energies of bulge loops. Should contain 31 entries, the first one
being INF.

# internal\_loop  
Free energies of internal loops. Should contain 31 entries, the first 4
being INF (since smaller loops are tabulated).

# mismatch\_interior  
Free energies for the interaction between the closing pair of an interior
loop and the two unpaired bases adjacent to the helix. This is a three
dimensional array indexed by the type of the closing pair (see above) and
the two unpaired bases. Since we distinguish 5 bases the list contains
8\*5\*5 entries and should be formated either as an 8\*25 matrix or 8 5\*5
matrices. The order is such that for example the mismatch

```
                               5'-CU-3'
                               3'-GC-5'
```

corresponds to entry [1,4,2] (CG=1, U=4, C=2), (in this notation
the first index runs from 1 to 7, second and third from 0 to 4)

# mismatch\_hairpin  
Same as above for hairpin loops.

# mismatch\_enthalpies  
Corresponding enthalpies for rescaling to temperatures other than 37C.

# int11\_energies  
Free energies for symmetric size 2 interior loops. 7\*7\*5\*5 entries formated
as 49 5\*5 matrices, or seven 7\*25 matrices. Example:

```
                               5'-CUU-3'
                               3'-GCA-5'
```

corresponds to entry [1,5,4,2], which should be identical to [5,1,2,4].

# int21\_energies  
Free energies for size 3 (2+1) interior loops. 7\*7\*5\*5\*5 entries formated
in 5\*5 or 5\*25 matrices. The strand with a single unpaired base is listed
first, example:

```
                               5'-CU U-3'
                               3'-GCCA-5'
```

corresponds to entry [1,5,4,2,2].

# int22\_energies  
Free energies for symmetric size 4 interior loops. To reduce the size of
parameter files this table only lists canonical bases (A,C,G,U) resulting in
a 7\*7\*4\*4\*4\*4 table. See above for an example.

# dangle5  
Energies for the interaction of an unpaired base on the 5' side and
adjacent to a helix in multiloops and free ends (the equivalent of mismatch
energies in interior and hairpin loops). The array is indexed by the type
of pair closing the helix and the unpaired base and, therefore, forms a 8\*5
matrix. For example the dangling base in

```
                               5'-C-3'
                               3'-GC-5'
```

corresponds to entry [1,2] (CG=1, C=2);

# dangle3  
Same as above for bases on the 3' side of a helix.

# ML\_params  
For the energy of a multi-loop a function of the form
`E = cu*n_unpaired + ci*loop_degree + cc`
is used where n\_unpaired is the number of unpaired bases in the loop and
loop\_degree is the number of helices forming the loop. In addition a
"terminal AU" penalty is applied to AU and GU pairs in the loop.
The line following the token should contain these four values, in the order
`cu cc ci termAU`. Ther terminal AU penalty is also used for the
exterior loop and size 3 hairpins, for other loop types it is already
included in the mismatch energies.

# Tetraloops  
Some tetraloops particularly stable tetraloops are assigned an energy
bonus. Up to forty tetraloops and their bonus energies can be listed
following the token, one sequence per line. For example:

```
       GAAA    -200
```

assigns a bonus energy of -2 kcal/mol to tetraloops containing
the sequence GAAA.

# END  
Anything beyond this token will be ignored.

A parameter file need not be complete, it might may contain only a subset
of interaction parameters, such as only stacking energies. However, for
each type of interaction listed, all entries have to be present.
A `` `*' `` may be used to indicate entries of a list that are to retain
their default value. For loop energies a `` `x' `` may be used to indicate that
the value is to be extrapolated from the values for smaller loop sizes.
Parameter files may contain C-style comments, i.e. any text between
`/*` and `*/` will be ignored. However, you may have no more
than one comment per line and no multi-line comments.

A parameter file listing the default parameter set should accompany your
distribution as `default.par', the file `old.par' contains
parameters used in version 1.1b of the Package.

# Parsing and Comparing of Structures

## Representations of Secondary Structures

The standard representation of a secondary structure is the "bracket
notation", where matching brackets symbolize base pairs and unpaired
bases are shown as dots. Alternatively, one may use two types of node
labels, 'P' for paired and 'U' for unpaired; a dot is then replaced by
'(U)', and each closed bracket is assigned an additional identifier 'P'.
We call this the expanded notation. In Fontana et al. (1993) a
condensed
representation of the secondary structure is proposed, the so-called
homeomorphically irreducible tree (HIT) representation. Here a stack is
represented as a single pair of matching brackets labeled 'P' and
weighted by the number of base pairs. Correspondingly, a contiguous
strain of unpaired bases is shown as one pair of matching brackets
labeled 'U' and weighted by its length. Generally any string consisting
of matching brackets and identifiers is equivalent to a plane tree with
as many different types of nodes as there are identifiers.

Bruce Shapiro (1988) proposed a coarse grained representation, which,
does not retain the full information of the secondary structure. He
represents the different structure elements by single matching brackets
and labels them as 'H' (hairpin loop), 'I' (interior loop), 'B'
(bulge), 'M' (multi-loop), and 'S' (stack). We extend his alphabet by an
extra letter for external elements 'E'. Again these identifiers may be
followed by a weight corresponding to the number of unpaired bases or
base pairs in the structure element. All tree representations (except
for the dot-bracket form) can be encapsulated into a virtual root
(labeled 'R'), see the example below.

The following example illustrates the different linear tree representations
used by the package. All lines show the same secondary structure.

```
a) .((((..(((...)))..((..)))).)).
   (U)(((((U)(U)((((U)(U)(U)P)P)P)(U)(U)(((U)(U)P)P)P)P)(U)P)P)(U)
b) (U)(((U2)((U3)P3)(U2)((U2)P2)P2)(U)P2)(U)
c) (((H)(H)M)B)
   ((((((H)S)((H)S)M)S)B)S)
   (((((((H)S)((H)S)M)S)B)S)E)
d) ((((((((H3)S3)((H2)S2)M4)S2)B1)S2)E2)R)
```

Above: Tree representations of secondary structures. a) Full structure:
the first line shows the more convenient condensed notation which is
used by our programs; the second line shows the rather clumsy expanded
notation for completeness, b) HIT structure, c) different versions of
coarse grained structures: the second line is exactly Shapiro's
representation, the first line is obtained by neglecting the stems.
Since each loop is closed by a unique stem, these two lines are
equivalent. The third line is an extension taking into account also the
external digits. d) weighted coarse structure, this time including the
virtual root.

For the output of aligned structures from string editing, different
representations are needed, where we put the label on both sides.
The above examples for tree representations would then look like:

```
a) (UU)(P(P(P(P(UU)(UU)(P(P(P(UU)(UU)(UU)P)P)P)(UU)(UU)(P(P(UU)(U...
b) (UU)(P2(P2(U2U2)(P2(U3U3)P3)(U2U2)(P2(U2U2)P2)P2)(UU)P2)(UU)
c) (B(M(HH)(HH)M)B)
   (S(B(S(M(S(HH)S)(S(HH)S)M)S)B)S)
   (E(S(B(S(M(S(HH)S)(S(HH)S)M)S)B)S)E)
d) (R(E2(S2(B1(S2(M4(S3(H3)S3)((H2)S2)M4)S2)B1)S2)E2)R)
```

Aligned structures additionally contain the gap character '\_'.

## Parsing and Coarse Graining of Structures

Several functions are provided for parsing structures and converting to
different representations.

Function: char\* **expand\_Full** *(char\* full)*: converts the full structure from bracket notation to the expanded notation including root.

Function: char\* **b2HIT** *(char\* full)*: converts the full structure from bracket notation to the HIT notation including root.

Function: char\* **b2C** *(char\* full)*: converts the full structure from bracket notation to the a coarse grained notation using the 'H' 'B' 'I' 'M' and 'R' identifiers.

Function: char\* **b2Shapiro** *(char\* full)*: converts the full structure from bracket notation to the *weighted* coarse grained notation using the 'H' 'B' 'I' 'M' 'S' 'E' and 'R' identifiers.

Function: char\* **expand\_Shapiro** *(char\* coarse)*: inserts missing 'S' identifiers in unweighted coarse grained structures as obtained from `b2C()`.

Function: char\* **add\_root** *(char\* any)*: adds a root to an un-rooted tree in any except bracket notation.

Function: char\* **unexpand\_Full** *(char\* expanded)*: restores the bracket notation from an expanded full or HIT tree, that is any tree using only identifiers 'U' 'P' and 'R'.

Function: char\* **unweight** *(char\* expanded)*: strip weights from any weighted tree.

All the above functions allocate memory for the strings they return.

Function: void **unexpand\_aligned\_F** *(char\* align[2])*: converts two aligned structures in expanded notation as produced by `tree_edit_distance()` function back to bracket notation with '\_' as the gap character. The result overwrites the input.

Function: void **parse\_structure** *(char\* full)*: Collects a statistic of structure elements of the full structure in bracket notation, writing to the following global variables:

Variable: int **loop\_size[]**: contains a list of all loop sizes. `loop_size[0]` contains the number of external bases.

Variable: int **loop\_degree[]**: contains the corresponding list of loop degrees.

Variable: int **helix\_size[]**: contains a list of all stack sizes.

Variable: int **loops**: contains the number of loops ( and therefore of stacks ).

Variable: int **pairs**: contains the number of base pairs in the last parsed structure.

Variable: int **unpaired**: contains the number of unpaired bases.

Prototypes for the above functions can be found in `RNAstruct.h'.

## Distance Measures

A simple measure of dissimilarity between secondary structures of equal
length is the base pair distance, given by the number of pairs present in
only one of the two structures being compared. I.e. the number of base
pairs that have to be opened or closed to transform one structure into the
other. It is therefore particularly useful for comparing structures on the
same sequence. It is implemented by

Function: int **bp\_distance** *(char\* s1, char\* s2)*: returns the "base pair" distance between two secondary structures s1 and s2, which should have the same length.

For other cases a distance measure that allows for gaps is preferable.
We can define distances between structures as edit distances between
trees or their string representations. In the case of string distances
this is the same as "sequence alignment". Given a set of edit operations
and edit costs, the edit distance is given by the minimum sum of the
costs along an edit path converting one object into the other. Edit
distances like these always define a metric. The edit operations used by us
are insertion, deletion and replacement of nodes.
String editing does not pay attention to the matching of brackets, while
in tree editing matching brackets represent a single node of the tree.
Tree editing is therefore usually preferable, although somewhat
slower. String edit distances are always smaller or equal to tree edit
distances.

The different level of detail in the structure representations defined
above naturally leads to different measures of distance. For full
structures we use a cost of 1 for deletion or insertion of an unpaired
base and 2 for a base pair. Replacing an unpaired base for a pair incurs
a cost of 1.

Two cost matrices are provided for coarse grained structures:

```
/*  Null,   H,   B,   I,   M,   S,   E     */
   {   0,   2,   2,   2,   2,   1,   1},   /* Null replaced */
   {   2,   0,   2,   2,   2, INF, INF},   /* H    replaced */
   {   2,   2,   0,   1,   2, INF, INF},   /* B    replaced */
   {   2,   2,   1,   0,   2, INF, INF},   /* I    replaced */
   {   2,   2,   2,   2,   0, INF, INF},   /* M    replaced */
   {   1, INF, INF, INF, INF,   0, INF},   /* S    replaced */
   {   1, INF, INF, INF, INF, INF,   0},   /* E    replaced */

/*  Null,   H,   B,   I,   M,   S,   E    */
   {   0, 100,   5,   5,  75,   5,   5},   /* Null replaced */
   { 100,   0,   8,   8,   8, INF, INF},   /* H    replaced */
   {   5,   8,   0,   3,   8, INF, INF},   /* B    replaced */
   {   5,   8,   3,   0,   8, INF, INF},   /* I    replaced */
   {  75,   8,   8,   8,   0, INF, INF},   /* M    replaced */
   {   5, INF, INF, INF, INF,   0, INF},   /* S    replaced */
   {   5, INF, INF, INF, INF, INF,   0},   /* E    replaced */
```

The lower matrix uses the costs given in Shapiro (1990).
All distance functions use the following global variables:

Variable: int **cost\_matrix**: if 0, use the default cost matrix (upper matrix in example); otherwise use Shapiro's costs (lower matrix).

Variable: int **edit\_backtrack**: produce an alignment of the two structures being compared by tracing the editing path giving the minimum distance.

Variable: char\* **aligned\_line[2]**: contains the two aligned structures after a call to one of the distance functions with `edit_backtrack` set to 1. See section Representations of Secondary Structures, for details on the representation of structures.

### Functions for Tree Edit Distances

Function: Tree\* **make\_tree** *(char\* xstruc)*: constructs a `Tree` ( essentially the postorder list ) of the structure xstruc, for use in `tree_edit_distance()`. xstruc may be any rooted structure representation.

Function: float **tree\_edit\_distance** *(Tree\* T1, Tree\* T2)*: calculates the edit distance of the two trees T1 and T2.

Function: void **free\_tree** *(Tree\* t)*: frees the memory allocated for t.

Prototypes for the above functions can be found in `treedist.h'. The
type `Tree` is defined in `dist\_vars.h', which is automatically
included with `treedist.h'

### Functions for String Alignment

Function: swString\* **Make\_swString** *(char\* xstruc)*: converts the structure xstruc into a format suitable for `string_edit_distance()`.

Function: float **string\_edit\_distance** *(swString\* T1, swString\* T2)*: calculates the string edit distance of T1 and T2.

Prototypes for the above functions can be found in `stringdist.h'.

### Functions for Comparison of Base Pair Probabilities

For comparison of base pair probability matrices, the matrices are first
condensed into probability profiles which are the compared by alignment.

Function: float\*\* **Make\_bp\_profile** *(int length)*: reads the base pair probability matrix `pr` (see section Global Variables for the Folding Routines) and calculates a profile, i.e. a vector containing for each base the probabilities of being unpaired, upstream, or downstream paired, respectively. The returned array is suitable for `profile_edit_distance`.

Function: float **profile\_edit\_distance** *(float\*\* T1, float\*\* T2)*: calculates an alignment distance of the two profiles T1 and T2.

Function: void **free\_profile** *(float\*\* T)*: frees the memory allocated for the profile T.

Prototypes for the above functions can be found in `profiledist.h'.

# Utilities

The following utilities are used and therefore provided by the library:

Function: int **PS\_dot\_plot** *(char\* sequence, char\* filename)*: reads base pair probabilities produced by `pf_fold()` from the global array `pr` and the pair list `base_pair` produced by `fold()` and produces a postscript "dot plot" that is written to filename. The "dot plot" represents each base pairing probability by a square of corresponding area in a upper triangle matrix. The lower part of the matrix contains the minimum free energy structure.

Function: int **PS\_rna\_plot** *(char\* sequence, char\* structure, char\* filename)*: produces a secondary structure graph in PostScript and writes it to filename. Note that this function has changed from previous versions and now expects the structure to be plotted in dot-bracket notation as an argument. It does not make use of the global `base_pair` array anymore.

Function: int **gmlRNA** *(char\* sequence, char\* structure, char\* filename, char option)*: produces a secondary structure graph in the Graph Meta Language gml and writes it to filename. If `option` is an uppercase letter the `sequence` is used to label nodes, if `option` equals `'X'` or `'x'` the resulting file will coordinates for an initial layout of the graph.

Variable: int **rna\_plot\_type**: switches between different layout algorithms for drawing secondary structures in `PS_rna_plot` and `gmlRNA`. Current possibility are 0 for a simple radial drawing or 1 for the modified radial drawing taken from the `naview` program of Bruccoleri & Heinrich (1988).

Function: char\* **random\_string** *(int l, char\* symbols)*: generates a "random" string of characters from symbols with length l.

Function: int **hamming** *(char\* s1, char\* s2)*: returns the number of positions in which s1 and s2 differ, the so called "Hamming" distance. s1 and s2 should have the same length.

Function: unsigned char\* **pack\_structure** *(char\* struc)*: returns a binary string encoding the secondary structure struc using a 5:1 compression scheme. The string is NULL terminated and can therefore be used with standard string functions such as strcmp(). Useful for programs that need to keep many structures in memory.

Function: char\* **unpack\_structure** *(unsigned char\* packed)*: translate a compressed binary string produced by pack\_structure() back into the familiar dot bracket notation.

Function: short\* **make\_pair\_table** *(char\* structure)*: returns a newly allocated table, such that: table[i]=j if (i.j) pair or 0 if i is unpaired, table[0] contains the length of the structure.

Function: char\* **time\_stamp** *(void)*: returns a string containing the current date in the format "Fri Mar 19 21:10:57 1993".

Function: void **nrerror** *(char\* message)*: writes message to stderr and aborts the program.

Function: double **urn** *()*: returns a pseudo random number in the range [0..1[, usually implemented by calling `erand48()`.

Variable: unsigned short **xsubi[3]**: is used by `urn ()`. These should be set to some random number seeds before the first call to `urn ()`.

Function: int **int\_urn** *(int from, int to)*: generates a pseudo random integer in the range [from, to].

Function: void\* **space** *(unsigned int size)*: returns a pointer to size bytes of allocated and 0 initialized memory; aborts with an error if memory is not available.

Function: char\* **get\_line** *(FILE\* fp)*: reads a line of arbitrary length from the stream \*fp, and returns a pointer to the resulting string. The necessary memory is allocated and should be released using `free()` when the string is no longer needed.

Prototypes for `PS_rna_plot()` and `PS_dot_plot()` reside in
`PS\_dot.h', the other functions are declared in `utils.h'.

# A Small Example Program

The following program exercises most commonly used functions of the library.
The program folds two sequences using both the mfe and partition function
algorithms and calculates the tree edit and profile distance of the
resulting structures and base pairing probabilities.

```
#include  <stdio.h>
#include  <math.h>
#include  "utils.h"
#include  "fold_vars.h"
#include  "fold.h"
#include  "part_func.h"
#include  "inverse.h"
#include  "RNAstruct.h"
#include  "treedist.h"
#include  "stringdist.h"
#include  "profiledist.h"

void main()
{
   char *seq1="CGCAGGGAUACCCGCG", *seq2="GCGCCCAUAGGGACGC",
        *struct1,* struct2,* xstruc;
   float e1, e2, tree_dist, string_dist, profile_dist, kT;
   Tree *T1, *T2;
   swString *S1, *S2;
   float **pf1, **pf2;

   /* fold at 30C instead of the default 37C */
   temperature = 30.;      /* must be set *before* initializing  */
   /* allocate memory for fold(), could be skipped */
   initialize_fold(strlen(seq1)); 

   /* allocate memory for structure and fold */
   struct1 = (char* ) space(sizeof(char)*(strlen(seq1)+1)); 
   e1 =  fold(seq1, struct1);

   struct2 = (char* ) space(sizeof(char)*(strlen(seq2)+1));
   e2 =  fold(seq2, struct2);

   free_arrays();     /* free arrays used in fold() */

   /* produce tree and string representations for comparison */
   xstruc = expand_Full(struct1);
   T1 = make_tree(xstruc);
   S1 = Make_swString(xstruc);
   free(xstruc);

   xstruc = expand_Full(struct2);
   T2 = make_tree(xstruc);
   S2 = Make_swString(xstruc);
   free(xstruc);

   /* calculate tree edit distance and aligned structures with gaps */
   edit_backtrack = 1;   
   tree_dist = tree_edit_distance(T1, T2);  
   free_tree(T1); free_tree(T2);
   unexpand_aligned_F(aligned_line);  
   printf("%s\n%s  %3.2f\n", aligned_line[0], aligned_line[1], tree_dist);

   /* same thing using string edit (alignment) distance */
   string_dist = string_edit_distance(S1, S2);
   free(S1); free(S2);
   printf("%s  mfe=%5.2f\n%s  mfe=%5.2f  dist=%3.2f\n",
        aligned_line[0], e1, aligned_line[1], e2, string_dist);

   /* for longer sequences one should also set a scaling factor for
      partition function folding, e.g: */
   kT = (temperature+273.15)*1.98717/1000.;  /* kT in kcal/mol */
   pf_scale = exp(-e1/kT/strlen(seq1));  
   init_pf_fold(strlen(seq1));  

   /* calculate partition function and base pair probabilities */
   e1 = pf_fold(seq1, struct1);
   pf1 = Make_bp_profile(strlen(seq1));

   e2 = pf_fold(seq2, struct2);
   pf2 = Make_bp_profile(strlen(seq2));
   
   free_pf_arrays();  /* free space allocated for pf_fold() */

   profile_dist = profile_edit_distance(pf1, pf2);
   printf("%s  free energy=%5.2f\n%s  free energy=%5.2f  dist=%3.2f\n",
        aligned_line[0], e1, aligned_line[1], e2, profile_dist);
   
   free_profile(pf1); free_profile(pf2);
}
```

In a typical Unix environment you would compile this program using:
`cc -c example.c -Ihpath`
and link using
`cc -o example -Llpath -lRNA -lm`
where hpath and lpath point to the location of the header
files and library, respectively.

# References

- D.H. Mathews, J. Sabina, M. Zucker and H. Turner (1999)  
  Expanded sequence dependence of thermodynamic parameters provides
  robust prediction of RNA secondary structure, JMB, 288: 911-940- M. Zuker and P. Stiegler (1981)  
    Optimal computer folding of large RNA sequences using
    thermodynamic and auxiliary information, Nucl Acid Res 9: 133-148- J.S. McCaskill (1990)  
      The equilibrium partition function and base pair binding
      probabilities for RNA secondary structures, Biopolymers 29: 1105-1119- D.H. Turner, N. Sugimoto and S.M. Freier (1988)  
        RNA structure prediction, Ann Rev Biophys Biophys Chem 17: 167-192- J.A. Jaeger, D.H. Turner and M. Zuker (1989)  
          Improved predictions of secondary structures for RNA,
          Proc. Natl. Acad. Sci. 86: 7706-7710- L. He, R. Kierzek, J. SantaLucia, A.E. Walter and D.H. Turner (1991)  
            Nearest-Neighbor Parameters For GU Mismatches,
            Biochemistry 30: 11124-11132- A.E. Peritz, R. Kierzek, N, Sugimoto, D.H. Turner (1991)  
              Thermodynamic Study of Internal Loops in Oligoribonucleotides ... ,
              Biochemistry 30: 6428--6435- A. Walter, D. Turner, J. Kim, M. Lyttle, P. M@"uller, D. Mathews and M. Zuker (1994)  
                Coaxial stacking of helices enhances binding of Oligoribonucleotides..,
                Proc. Natl. Acad. Sci. 91: 9218-9222- B.A. Shapiro, (1988)  
                  An algorithm for comparing multiple RNA secondary structures,
                  CABIOS 4, 381-393- B.A. Shapiro and K. Zhang (1990)  
                    Comparing multiple RNA secondary structures using tree comparison,
                    CABIOS 6, 309-318- R. Bruccoleri and G. Heinrich (1988)  
                      An improved algorithm for nucleic acid secondary structure display,
                      CABIOS 4, 167-173- W. Fontana , D.A.M. Konings, P.F. Stadler, P. Schuster (1993)   
                        Statistics of RNA secondary structures, Biopolymers 33, 1389-1404- W. Fontana, P.F. Stadler, E.G. Bornberg-Bauer, T. Griesmacher, I.L.
                          Hofacker, M. Tacker, P. Tarazona, E.D. Weinberger, P. Schuster (1993)  
                          RNA folding and combinatory landscapes, Phys. Rev. E 47: 2083-2099- I.L. Hofacker, W. Fontana, P.F. Stadler, S. Bonhoeffer, M. Tacker, P.
                            Schuster (1994) Fast Folding and Comparison of RNA Secondary Structures.
                            Monatshefte f. Chemie 125: 167-188- I.L. Hofacker (1994) The Rules of the Evolutionary Game for RNA:
                              A Statistical Characterization of the Sequence to Structure Mapping in RNA.
                              PhD Thesis, University of Vienna.- D. Adams (1979)   
                                The hitchhiker's guide to the galaxy, Pan Books, London

# Function Index

Jump to:
a
-
b
-
e
-
f
-
g
-
h
-
i
-
m
-
n
-
p
-
r
-
s
-
t
-
u
-
w

## a

- add\_root

## b

- b2C- b2HIT- b2Shapiro- bp\_distance

## e

- energy\_of\_struct- expand\_Full- expand\_Shapiro

## f

- fold- free\_arrays- free\_pf\_arrays- free\_profile- free\_tree

## g

- get\_line- gmlRNA

## h

- hamming

## i

- init\_pf\_fold- initialize\_fold- int\_urn- inverse\_fold- inverse\_pf\_fold

## m

- Make\_bp\_profile- make\_pair\_table- Make\_swString- make\_tree

## n

- nrerror

## p

- pack\_structure- parse\_structure- pf\_fold- profile\_edit\_distance- PS\_dot\_plot- PS\_rna\_plot

## r

- random\_string- read\_parameter\_file

## s

- space- string\_edit\_distance

## t

- time\_stamp- tree\_edit\_distance

## u

- unexpand\_aligned\_F- unexpand\_Full- unpack\_structure- unweight- update\_fold\_params- update\_pf\_params- urn

## w

- write\_parameter\_file

# Variable Index

Jump to:
\*
-
a
-
b
-
c
-
d
-
e
-
f
-
g
-
h
-
i
-
l
-
n
-
p
-
r
-
t
-
u
-
x

## \*

- \*symbolset

## a

- aligned\_line[2]

## b

- backtrack\_type- base\_pair

## c

- cost\_matrix

## d

- dangles- do\_backtrack

## e

- edit\_backtrack- energy\_set

## f

- fold\_constrained

## g

- give\_up

## h

- helix\_size[]

## i

- iindx

## l

- loop\_degree[]- loop\_size[]- loops

## n

- no\_closingGU- noGU- noLonelyPairs- nonstandards

## p

- pairs- pf\_scale- pr

## r

- rna\_plot\_type

## t

- temperature- tetra\_loop

## u

- unpaired

## x

- xsubi[3]

---

This document was generated on 28 September 2000 using
texi2html 1.56k.
