# Supplementary material for: Improving model predictions for RNA interference activities that use support vector machine regression by combining and filtering features
Source: BMC Bioinformatics. 2007 Jun 6;8:182. doi: 10.1186/1471-2105-8-182 (PMC1906837; doi:10.1186/1471-2105-8-182)
Supplement: Additional file 7 — seq2svm_0.3. An GNU platform deployable GPL code base for performing SVM modeling on small RNA sequences, with examples. Deploy by unzipping, untarring, and building with configure and make. See the included readme files. Updated versions will be available at . [file 1471-2105-8-182-S7.gz › seq2svm/src/libRNAfold-2.4/ViennaRNA-1.4/man/RNAlib.pdf]

Vienna RNA Package

A Library for folding and comparing RNA secondary structures

Copyright ©1994-2000

Ivo Hofacker and Peter Stadler

Revision $Id: RNAlib.texinfo,v 1.6 1998/07/07 16:46:21 ivo Exp ivo $

1 Introduction

The core of the Vienna RNA Package is formed by a collection of routines for the prediction and comparison of RNA secondary structures. These routines can be accessed through stand-alone programs, such as RNAfold, RNAdistance etc., which should be sufficient for most users. For those who wish to develop their own programs we provide a library which can be linked to your own code.

This document only describes the library and will be primarily useful to programmers. The stand-alone programs are described in separate man pages. The latest version of the package including source code and html versions of the documentation can be found at http://www.tbi.univie.ac.at/~ivo/RNA/ . This manual documents version 1.3.

Please send comments and bug reports to ivo@tbi.univie.ac.at.

2 Folding Routines

2.1 Minimum free Energy Folding

The library provides a fast dynamic programming minimum free energy folding algorithm as described by *Zuker & Stiegler (1981)*. Associated functions are:

`float` **fold** (`char*` *sequence*, `char*` *structure*) Function

folds the *sequence* and returns the minimum free energy in kcal/mol; the mfe structure in bracket notation (see Section 3.1 [notations], page 8) is returned in *structure*. Sufficient space for string of the same length as *sequence* must be allocated for *structure* before calling `fold()`. If `fold_constrained` (see Section 2.4 [Variables], page 4) is 1, the *structure* string is interpreted on input as a list of constraints for the folding. The characters " `|` x `<` `>` " mark bases that are paired, unpaired, paired upstream, or downstream, respectively; matching brackets " ( ) " denote base pairs, dots "." are used for unconstrained bases. Constrained folding works by assigning bonus energies to all structures compliing with the constraint.

`float` **energy_of_struct** (`char*` *sequence*, `char*` *structure*) Function

calculates the energy of *sequence* on the *structure*

`void` **initialize_fold** (`int` *length*) Function

allocates memory for folding sequences not longer than *length*; sets up pairing matrix and energy parameters. Has to be called before the first call to `fold()`.

`void` **free_arrays** () Function

frees the memory allocated by `initialize_fold()`.

`void` **update_fold_params** () Function

call this to recalculate the pair matrix and energy parameters after a change in folding parameters like `temperature` (see Section 2.4 [Variables], page 4).

Prototypes for these functions are declared in '`fold.h`'.

2.2 Partition Function Folding

Instead of the minimum free energy structure the partition function of all possible structures and from that the pairing probability for every possible pair can be calculated, using a dynamic programming algorithm as described by *McCaskill (1990)*. The following functions are provided:

`float` **pf_fold** (`char*` *sequence*, `char*` *structure*) Function

calculates the partition function Z of *sequence* and returns the free energy of the ensemble F in kcal/mol, where $F = -kTln(Z)$. If *structure* is not a NULL pointer on input, it contains on return a string consisting of the letters " . , `|` `{` `}` ( ) " denoting bases that are essentially unpaired, weakly paired, strongly paired without preference, weakly upstream (downstream) paired, or strongly up- (down-)stream paired bases, respectively. If `fold_constrained` (see Section 2.4 [Variables], page 4) is 1, the *structure* string is interpreted on

input as a list of constraints for the folding. The character "x" marks bases that must be unpaired, matching brackets " ( ) " denote base pairs, all other characters are ignored. Any pairs conflicting with the constraint will be forbidden. This usually sufficient to ensure the constraints are honored. If `do_backtrack` (see Section 2.4 [Variables], page 4) has been set to 0 base pairing probabilities will not be computed (saving CPU time), otherwise the `pr[iindx[i]-j]` (see Section 2.4 [Variables], page 4) will contain the probability that bases i and j pair.

`void` **init_pf_fold** (`int` *length*) Function

allocates memory for folding sequences not longer than *length*; sets up pairing matrix and energy parameters. Has to be called before the first call to `pf_fold()`.

`void` **free_pf_arrays** (`void`) Function

frees the memory allocated by `init_pf_fold()`.

`void` **update_pf_params** (`int` *length*) Function

Call this function to recalculate the pair matrix and energy parameters after a change in folding parameters like temperature (see Section 2.4 [Variables], page 4).

Prototypes for these functions are declared in '`part_func.h`'.

2.3 Inverse Folding

We provide two functions that search for sequences with a given structure, thereby inverting the folding routines.

`float` **inverse_fold** (`char*` *start*, `char*` *target*) Function

searches for a sequence with minimum free energy structure *target*, starting with sequence *start*. It returns 0 if the search was successful, otherwise a structure distance to *target* is returned. The found sequence is returned in *start*. If `give_up` is set to 1, the function will return as soon as it is clear that the search will be unsuccessful, this speeds up the algorithm if you are only interested in exact solutions. Since `inverse_fold()` calls `fold()` you have to allocate memory for folding by calling `initialize_fold()`

`float` **inverse_pf_fold** (`char*` *start*, `char*` *target*) Function

searches for a sequence with maximum probability to fold into structure *target* using the partition function algorithm. It returns -kT log(p) where p is the frequency of *target* in the ensemble of possible structures. This is usually much slower than `inverse_fold()`. Since `inverse_pf_fold()` calls `pf_fold()` you have to allocate memory for folding by calling `init_pf_fold()`

`char` ***symbolset** Variable

The global variable `char *symbolset` points to the allowed bases, initially `"AUGC"`. It can be used to design sequences from reduced alphabets.

Prototypes for these functions are declared in '`inverse.h`'.

2.4 Global Variables for the Folding Routines

The following global variables change the behavior the folding algorithms or contain additional information after folding.

`int` **noGU** Variable

do not allow GU pairs if equal 1; default is 0.

`int` **no_closingGU** Variable

if 1 allow GU pairs only inside stacks, not as closing pairs; default is 0.

`int` **noLonelyPairs** Variable

Disallow all pairs which can **only** occur as lonely pairs (i.e. as helix of length 1). This avoids lonely base pairs in the predicted structures in most cases.

`int` **tetra_loop** Variable

include special stabilizing energies for some tetra loops; default is 1.

`int` **energy_set** Variable

if 1 or 2: fold sequences from an artificial alphabet ABCD..., where A pairs B, C pairs D, etc. using either GC (1) or AU parameters (2); default is 0, you probably don't want to change it.

`float` **temperature** Variable

rescale energy parameters to a temperature of `temperature` C. Default is 37C. You have to call the update_..._params() functions after changing this parameter.

`int` **dangles** Variable

if set to 0 no stabilizing energies are assigned to bases adjacent to helices in free ends and multiloops (so called dangling ends). Normally (`dangles = 1`) dangling end energies are assigned only to unpaired bases and a base cannot participate simultaneously in two dangling ends. In the partition function algorithm `pf_fold()` these checks are neglected. If `dangles` is set to 2, the `fold()` and `energy_of_struct()` function will also follow this convention. This treatment of dangling ends gives more favorable energies to helices directly adjacent to one another, which can be beneficial since such helices often do engage in stabilizing interactions through co-axial stacking.
If `dangles = 3` co-axial stacking is explicitely included for adjacent helices in mutli-loops. The option affects only mfe folding and energy evaluation (`fold()` and `energy_of_struct()`), as well as suboptimal folding via re-evaluation of energies. Co-axial stacking with one intervening mismatch is not considered so far.

Default is 1, `pf_fold()` treats 1 as 2.

`char*` **nonstandards** Variable

Lists additional base pairs that will be allowed to form in addition to GC, CG, AU, UA, GU and UG. Nonstandard base pairs are given a stacking energy of 0.

`struct bond { int i,j;}` **base_pair** Variable

Contains a list of base pairs after a call to `fold()`. `base_pair[0].i` contains the total number of pairs.

`double* pr` **pr** Variable

contains the base pair probability matrix after a call to `pf_fold()`.

`int*` **iindx** Variable

index array to move through pr. The probability for base i and j to form a pair is in `pr[iindx[i]-j]`.

`float` **pf_scale** Variable

a scaling factor used by `pf_fold()` to avoid overflows. Should be set to approximately $\exp((-F/kT)/length)$, where F is an estimate for the ensemble free energy, for example the minimum free energy. You must call `update_pf_params()` or `init_pf_fold()` after changing this parameter. If pf_scale is -1 (the default) , an estimate will be provided automatically when calling `init_pf_fold()` or `update_pf_params()`. The automatic estimate is usually insufficient for sequences more than a few hundred bases long.

`int` **fold_constrained** Variable

If 1, calculate constrained minimum free energy structures. See Section 2.1 [mfe Fold], page 2, for more information. Default is 0;

`int` **do_backtrack** Variable

if 0, do not calculate pair probabilities in `pf_fold()`; this is about twice as fast. Default is 1.

`char` **backtrack_type** Variable

only for use by `inverse_fold()`; 'C': force (1,N) to be paired, 'M' fold as if the sequence were inside a multi-loop. Otherwise the usual mfe structure is computed.

include '`fold_vars.h`' if you want to change any of these variables from their defaults.

2.5 Energy Parameter Files

A default set of parameters, identical to the one described in *Mathews et.al. (1999)*, is compiled into the library. Alternately, parameters can be read from and written to a file.

`void` **read_parameter_file** (`const char` *fname*[]) Function

reads energy parameters from file *fname*. See below for the format of the parameter file.

`void` **write_parameter_file** (`const char` *fname*[]) Function

writes current energy parameters to the file *fname*.

The following describes the file format expected by `read_parameter_file()`. All energies should be given as integers in units of 0.01kcal/mol.

Various loop parameters depend in general on the pairs closing the loops, as well as unpaired bases in the loops. Internally, the library distinguishes 8 types of pairs (CG=1, GC=2, GU=3, UG=4, AU=5, UA=6, nonstandard=7, 0= no pair), and 5 types of bases (A=1, C=2, G=3, U=4 and 0 for anything else). Parameters belonging to pairs of type 0 are not listed in the parameter files, but values for nonstandard pairs (type 7) and nonstandard bases (type 0) are. Thus, a table for symmetric size 2 interior loops would have 7*7*5*5 entries (2 pairs, two unpaired bases).

The order of entries always uses the closing pair or pairs as the first indices followed by the unpaired bases in 5' to 3' direction. To determine the type of a pair consider the base at 5' end of each strand first, i.e. use the pairs (i,j) and (q,p) for an interior loop with $i<p<q<j$. This is probably better explained by an example. Consider the symmetric size 4 interior loop

```
5'-GAUA-3'
3'-CGCU-5'
```

the first pair is GC, the second UA (not AU!) the unpaired bases are (in 5' to 3' direction, starting at the first pair) A U C G. Thus we need entry [2,6,1,4,2,3] of the corresponding table. Because the loop is symmetric you could equally well describe it by UA GC C G A U, i.e. entry [6,2,2,3,1,4]. Be careful to preserve this symmetry when editing parameter tables!

The first line of the file should read

`## RNAfold parameter file`

lines of the form

`# token`

mark the beginning of a list of energy parameters of the type specified by token. The following tokens are recognized:

`# stack_energies`

The list of free energies for stacked pairs, indexed by the two closing pairs. The list should be formated as symmetric an 7*7 matrix,conforming to the order explained above. As an example the stacked pair

```
5'-GU-3'        5'-AC-3'
3'-CA-5'        3'-UG-5'
```

corresponds to the entry [2,5], which should be identical to [5,2]. Note that the format has changed from previous releases, to make it consistent with other loop parameters.

`# stack_enthalpies`

enthalpies for stacked pairs, used to rescale stacking energies to temperatures other than 37C. Same format as stack_energies.

`# hairpin`

Free energies of hairpin loops as a function of size. The list should contain 31 entries on one or more lines. Since the minimum size of a hairpin loop is 3 and we start counting with 0, the first three values should be INF to indicate a forbidden value.

`# bulge`

Free energies of bulge loops. Should contain 31 entries, the first one being INF.

`# internal_loop`

Free energies of internal loops. Should contain 31 entries, the first 4 being INF (since smaller loops are tabulated).

`# mismatch_interior`

Free energies for the interaction between the closing pair of an interior loop and the two unpaired bases adjacent to the helix. This is a three dimensional array indexed by the type of the closing pair (see above) and the two unpaired bases. Since we distinguish 5 bases the list contains 8*5*5 entries and should be formated either as an 8*25 matrix or 8 5*5 matrices. The order is such that for example the mismatch

```
5'-CU-3'
3'-GC-5'
```

corresponds to entry [1,4,2] (CG=1, U=4, C=2), (in this notation the first index runs from 1 to 7, second and third from 0 to 4)

`# mismatch_hairpin`

Same as above for hairpin loops.

`# mismatch_enthalpies`

Corresponding enthalpies for rescaling to temperatures other than 37C.

`# int11_energies`

Free energies for symmetric size 2 interior loops. 7*7*5*5 entries formated as 49 5*5 matrices, or seven 7*25 matrices. Example:

```
5'-CUU-3'
3'-GCA-5'
```

corresponds to entry [1,5,4,2], which should be identical to [5,1,2,4].

`# int21_energies`
Free energies for size 3 (2+1) interior loops. 7*7*5*5*5 entries formated in 5*5 or 5*25 matrices. The strand with a single unpaired base is listed first, example:

```
5'-CU U-3'
3'-GCCA-5'
```

corresponds to entry [1,5,4,2,2].

`# int22_energies`
Free energies for symmetric size 4 interior loops. To reduce the size of parameter files this table only lists canonical bases (A,C,G,U) resulting in a 7*7*4*4*4*4 table. See above for an example.

`# dangle5`
Energies for the interaction of an unpaired base on the 5' side and adjacent to a helix in multiloops and free ends (the equivalent of mismatch energies in interior and hairpin loops). The array is indexed by the type of pair closing the helix and the unpaired base and, therefore, forms a 8*5 matrix. For example the dangling base in

```
5'-C-3'
3'-GC-5'
```

corresponds to entry [1,2] (CG=1, C=2);

`# dangle3`
Same as above for bases on the 3' side of a helix.

`# ML_params`
For the energy of a multi-loop a function of the form `E = cu*n_unpaired + ci*loop_degree + cc` is used where n_unpaired is the number of unpaired bases in the loop and loop_degree is the number of helices forming the loop. In addition a "terminal AU" penalty is applied to AU and GU pairs in the loop. The line following the token should contain these four values, in the order `cu cc ci termAU`. Ther terminal AU penalty is also used for the exterior loop and size 3 hairpins, for other loop types it is already included in the mismatch energies.

`# Tetraloops`
Some tetraloops particularly stable tetraloops are assigned an energy bonus. Up to forty tetraloops and their bonus energies can be listed following the token, one sequence per line. For example:

```
GAAA    -200
```

assigns a bonus energy of -2 kcal/mol to tetraloops containing the sequence GAAA.

`# END`
Anything beyond this token will be ignored.

A parameter file need not be complete, it might may contain only a subset of interaction parameters, such as only stacking energies. However, for each type of interaction listed, all entries have to be present. A '`*`' may be used to indicate entries of a list that are to retain their default value. For loop energies a '`x`' may be used to indicate that the value is to be extrapolated from the values for smaller loop sizes. Parameter files may contain C-style comments, i.e. any text between `/*` and `*/` will be ignored. However, you may have no more than one comment per line and no multi-line comments.

A parameter file listing the default parameter set should accompany your distribution as '`default.par`', the file '`old.par`' contains parameters used in version 1.1b of the Package.

3 Parsing and Comparing of Structures

3.1 Representations of Secondary Structures

The standard representation of a secondary structure is the "bracket notation", where matching brackets symbolize base pairs and unpaired bases are shown as dots. Alternatively, one may use two types of node labels, 'P' for paired and 'U' for unpaired; a dot is then replaced by '(U)', and each closed bracket is assigned an additional identifier 'P'. We call this the expanded notation. In *Fontana et al. (1993)* a condensed representation of the secondary structure is proposed, the so-called homeomorphically irreducible tree (HIT) representation. Here a stack is represented as a single pair of matching brackets labeled 'P' and weighted by the number of base pairs. Correspondingly, a contiguous strain of unpaired bases is shown as one pair of matching brackets labeled 'U' and weighted by its length. Generally any string consisting of matching brackets and identifiers is equivalent to a plane tree with as many different types of nodes as there are identifiers.

Bruce Shapiro (1988) proposed a coarse grained representation, which, does not retain the full information of the secondary structure. He represents the different structure elements by single matching brackets and labels them as 'H' (hairpin loop), 'I' (interior loop), 'B' (bulge), 'M' (multi-loop), and 'S' (stack). We extend his alphabet by an extra letter for external elements 'E'. Again these identifiers may be followed by a weight corresponding to the number of unpaired bases or base pairs in the structure element. All tree representations (except for the dot-bracket form) can be encapsulated into a virtual root (labeled 'R'), see the example below.

The following example illustrates the different linear tree representations used by the package. All lines show the same secondary structure.

```
a) .((((..(((...)))..((..)))).)).
   (U)((((U)(U)(((U)(U)(U)P)P)P)(U)(U)(((U)(U)P)P)P)P)(U)P)P)(U)
b) (U)(((U2)((U3)P3)(U2)((U2)P2)P2)(U)P2)(U)
c) (((H)(H)M)B)
   ((((((H)S)((H)S)M)S)B)S)
   (((((((H)S)((H)S)M)S)B)S)E)
d) ((((((((H3)S3)((H2)S2)M4)S2)B1)S2)E2)R)
```

Above: Tree representations of secondary structures. a) Full structure: the first line shows the more convenient condensed notation which is used by our programs; the second line shows the rather clumsy expanded notation for completeness, b) HIT structure, c) different versions of coarse grained structures: the second line is exactly Shapiro's representation, the first line is obtained by neglecting the stems. Since each loop is closed by a unique stem, these two lines are equivalent. The third line is an extension taking into account also the external digits. d) weighted coarse structure, this time including the virtual root.

For the output of aligned structures from string editing, different representations are needed, where we put the label on both sides. The above examples for tree representations would then look like:

```
a) (UU)(P(P(P(P(UU)(UU)(P(P(P(UU)(UU)(UU)P)P)P)(UU)(UU)(P(P(UU)(U...
b) (UU)(P2(P2(U2U2)(P2(U3U3)P3)(U2U2)(P2(U2U2)P2)P2)(UU)P2)(UU)
c) (B(M(HH)(HH)M)B)
   (S(B(S(M(S(HH)S)(S(HH)S)M)S)B)S)
   (E(S(B(S(M(S(HH)S)(S(HH)S)M)S)B)S)E)
d) (R(E2(S2(B1(S2(M4(S3(H3)S3)((H2)S2)M4)S2)B1)S2)E2)R)
```

Aligned structures additionally contain the gap character '_'.

3.2 Parsing and Coarse Graining of Structures

Several functions are provided for parsing structures and converting to different representations.

`char*` **expand_Full** (`char*` *full*) Function
: converts the *full* structure from bracket notation to the expanded notation including root.

`char*` **b2HIT** (`char*` *full*) Function
: converts the *full* structure from bracket notation to the HIT notation including root.

`char*` **b2C** (`char*` *full*) Function
: converts the *full* structure from bracket notation to the a coarse grained notation using the 'H' 'B' 'I' 'M' and 'R' identifiers.

`char*` **b2Shapiro** (`char*` *full*) Function
: converts the *full* structure from bracket notation to the *weighted* coarse grained notation using the 'H' 'B' 'I' 'M' 'S' 'E' and 'R' identifiers.

`char*` **expand_Shapiro** (`char*` *coarse*) Function
: inserts missing 'S' identifiers in unweighted coarse grained structures as obtained from `b2C()`.

`char*` **add_root** (`char*` *any*) Function
: adds a root to an un-rooted tree in any except bracket notation.

`char*` **unexpand_Full** (`char*` *expanded*) Function
: restores the bracket notation from an expanded full or HIT tree, that is any tree using only identifiers 'U' 'P' and 'R'.

`char*` **unweight** (`char*` *expanded*) Function
: strip weights from any weighted tree.

All the above functions allocate memory for the strings they return.

`void` **unexpand_aligned_F** (`char*` *align*[2]) Function
: converts two aligned structures in expanded notation as produced by `tree_edit_distance()` function back to bracket notation with '_' as the gap character. The result overwrites the input.

`void` **parse_structure** (`char*` *full*) Function
: Collects a statistic of structure elements of the *full* structure in bracket notation, writing to the following global variables:

`int` **loop_size**[] Variable
: contains a list of all loop sizes. `loop_size[0]` contains the number of external bases.

`int` **loop_degree**[] Variable
: contains the corresponding list of loop degrees.

`int` **helix_size**[] Variable

contains a list of all stack sizes.

`int` **loops** Variable

contains the number of loops (and therefore of stacks).

`int` **pairs** Variable

contains the number of base pairs in the last parsed structure.

`int` **unpaired** Variable

contains the number of unpaired bases.

Prototypes for the above functions can be found in '`RNAstruct.h`'.

3.3 Distance Measures

A simple measure of dissimilarity between secondary structures of equal length is the base pair distance, given by the number of pairs present in only one of the two structures being compared. I.e. the number of base pairs that have to be opened or closed to transform one structure into the other. It is therefore particularly useful for comparing structures on the same sequence. It is implemented by

`int` **bp_distance** (`char*` *s1*, `char*` *s2*) Function

returns the "base pair" distance between two secondary structures *s1* and *s2*, which should have the same length.

For other cases a distance measure that allows for gaps is preferable. We can define distances between structures as edit distances between trees or their string representations. In the case of string distances this is the same as "sequence alignment". Given a set of edit operations and edit costs, the edit distance is given by the minimum sum of the costs along an edit path converting one object into the other. Edit distances like these always define a metric. The edit operations used by us are insertion, deletion and replacement of nodes. String editing does not pay attention to the matching of brackets, while in tree editing matching brackets represent a single node of the tree. Tree editing is therefore usually preferable, although somewhat slower. String edit distances are always smaller or equal to tree edit distances.

The different level of detail in the structure representations defined above naturally leads to different measures of distance. For full structures we use a cost of 1 for deletion or insertion of an unpaired base and 2 for a base pair. Replacing an unpaired base for a pair incurs a cost of 1.

Two cost matrices are provided for coarse grained structures:

```
/*  Null,   H,   B,   I,   M,   S,   E     */
   {   0,   2,   2,   2,   2,   1,   1},   /* Null replaced */
   {   2,   0,   2,   2,   2, INF, INF},   /* H    replaced */
   {   2,   2,   0,   1,   2, INF, INF},   /* B    replaced */
   {   2,   2,   1,   0,   2, INF, INF},   /* I    replaced */
   {   2,   2,   2,   2,   0, INF, INF},   /* M    replaced */
   {   1, INF, INF, INF, INF,   0, INF},   /* S    replaced */
   {   1, INF, INF, INF, INF, INF,   0},   /* E    replaced */


/*  Null,   H,   B,   I,   M,   S,   E    */
   {   0, 100,   5,   5,  75,   5,   5},   /* Null replaced */
```

```
{ 100,   0,   8,   8,   8, INF, INF},   /* H   replaced */
{   5,   8,   0,   3,   8, INF, INF},   /* B   replaced */
{   5,   8,   3,   0,   8, INF, INF},   /* I   replaced */
{  75,   8,   8,   8,   0, INF, INF},   /* M   replaced */
{   5, INF, INF, INF, INF,   0, INF},   /* S   replaced */
{   5, INF, INF, INF, INF, INF,   0},   /* E   replaced */
```

The lower matrix uses the costs given in *Shapiro (1990)*. All distance functions use the following global variables:

`int` **cost_matrix** — Variable

if 0, use the default cost matrix (upper matrix in example); otherwise use Shapiro's costs (lower matrix).

`int` **edit_backtrack** — Variable

produce an alignment of the two structures being compared by tracing the editing path giving the minimum distance.

`char*` **aligned_line[2]** — Variable

contains the two aligned structures after a call to one of the distance functions with `edit_backtrack` set to 1. See Section 3.1 [notations], page 8, for details on the representation of structures.

3.3.1 Functions for Tree Edit Distances

`Tree*` **make_tree** (`char*` *xstruc*) — Function

constructs a `Tree` (essentially the postorder list) of the structure *xstruc*, for use in `tree_edit_distance()`. *xstruc* may be any rooted structure representation.

`float` **tree_edit_distance** (`Tree*` *T1*, `Tree*` *T2*) — Function

calculates the edit distance of the two trees *T1* and *T2*.

`void` **free_tree** (`Tree*` *t*) — Function

frees the memory allocated for *t*.

Prototypes for the above functions can be found in '`treedist.h`'. The type `Tree` is defined in '`dist_vars.h`', which is automatically included with '`treedist.h`'

3.3.2 Functions for String Alignment

`swString*` **Make_swString** (`char*` *xstruc*) — Function

converts the structure *xstruc* into a format suitable for `string_edit_distance()`.

`float` **string_edit_distance** (`swString*` *T1*, `swString*` *T2*) — Function

calculates the string edit distance of *T1* and *T2*.

Prototypes for the above functions can be found in '`stringdist.h`'.

3.3.3 Functions for Comparison of Base Pair Probabilities

For comparison of base pair probability matrices, the matrices are first condensed into probability profiles which are the compared by alignment.

`float**` **Make_bp_profile** (`int` *length*) Function

reads the base pair probability matrix `pr` (see Section 2.4 [Variables], page 4) and calculates a profile, i.e. a vector containing for each base the probabilities of being unpaired, upstream, or downstream paired, respectively. The returned array is suitable for `profile_edit_distance`.

`float` **profile_edit_distance** (`float**` *T1*, `float**` *T2*) Function

calculates an alignment distance of the two profiles *T1* and *T2*.

`void` **free_profile** (`float**` *T*) Function

frees the memory allocated for the profile *T*.

Prototypes for the above functions can be found in ‘`profiledist.h`’.

4 Utilities

The following utilities are used and therefore provided by the library:

`int` **PS_dot_plot** (`char*` *sequence*, `char*` *filename*) Function

reads base pair probabilities produced by `pf_fold()` from the global array `pr` and the pair list `base_pair` produced by `fold()` and produces a postscript "dot plot" that is written to *filename*. The "dot plot" represents each base pairing probability by a square of corresponding area in a upper triangle matrix. The lower part of the matrix contains the minimum free energy structure.

`int` **PS_rna_plot** (`char*` *sequence*, `char*` *structure*, `char*` *filename*) Function

produces a secondary structure graph in PostScript and writes it to *filename*. Note that this function has changed from previous versions and now expects the structure to be plotted in dot-bracket notation as an argument. It does not make use of the global `base_pair` array anymore.

`int` **gmlRNA** (`char*` *sequence*, `char*` *structure*, `char*` *filename*, `char` *option*) Function

produces a secondary structure graph in the Graph Meta Language gml and writes it to *filename*. If `option` is an uppercase letter the `sequence` is used to label nodes, if `option` equals *'X'* or *'x'* the resulting file will coordinates for an initial layout of the graph.

`int` **rna_plot_type** Variable

switches between different layout algorithms for drawing secondary structures in `PS_rna_plot` and `gmlRNA`. Current possibility are 0 for a simple radial drawing or 1 for the modified radial drawing taken from the `naview` program of *Bruccoleri & Heinrich (1988)*.

`char*` **random_string** (`int` *l*, `char*` *symbols*) Function

generates a "random" string of characters from *symbols* with length *l*.

`int` **hamming** (`char*` *s1*, `char*` *s2*) Function

returns the number of positions in which *s1* and *s2* differ, the so called "Hamming" distance. *s1* and *s2* should have the same length.

`unsigned char*` **pack_structure** (`char*` *struc*) Function

returns a binary string encoding the secondary structure *struc* using a 5:1 compression scheme. The string is NULL terminated and can therefore be used with standard string functions such as strcmp(). Useful for programs that need to keep many structures in memory.

`char*` **unpack_structure** (`unsigned char*` *packed*) Function

translate a compressed binary string produced by pack_structure() back into the familiar dot bracket notation.

`short*` **make_pair_table** (`char*` *structure*) Function

returns a newly allocated table, such that: table[i]=j if (i.j) pair or 0 if i is unpaired, table[0] contains the length of the *structure*.

`char*` **time_stamp** (`void`) Function

returns a string containing the current date in the format "Fri Mar 19 21:10:57 1993".

void **nrerror** (char* *message*) Function

writes *message* to stderr and aborts the program.

double **urn** () Function

returns a pseudo random number in the range [0..1[, usually implemented by calling `erand48()`.

unsigned short **xsubi[3]** Variable

is used by `urn ()`. These should be set to some random number seeds before the first call to `urn ()`.

int **int_urn** (int *from*, int *to*) Function

generates a pseudo random integer in the range [*from*, *to*].

void* **space** (unsigned int *size*) Function

returns a pointer to *size* bytes of allocated and 0 initialized memory; aborts with an error if memory is not available.

char* **get_line** (FILE* *fp*) Function

reads a line of arbitrary length from the stream **fp*, and returns a pointer to the resulting string. The necessary memory is allocated and should be released using `free()` when the string is no longer needed.

Prototypes for `PS_rna_plot()` and `PS_dot_plot()` reside in '`PS_dot.h`', the other functions are declared in '`utils.h`'.

5 A Small Example Program

The following program exercises most commonly used functions of the library. The program folds two sequences using both the mfe and partition function algorithms and calculates the tree edit and profile distance of the resulting structures and base pairing probabilities.

```
#include  <stdio.h>
#include  <math.h>
#include  "utils.h"
#include  "fold_vars.h"
#include  "fold.h"
#include  "part_func.h"
#include  "inverse.h"
#include  "RNAstruct.h"
#include  "treedist.h"
#include  "stringdist.h"
#include  "profiledist.h"

void main()
{
   char *seq1="CGCAGGGAUACCCGCG", *seq2="GCGCCCAUAGGGACGC",
        *struct1,* struct2,* xstruc;
   float e1, e2, tree_dist, string_dist, profile_dist, kT;
   Tree *T1, *T2;
   swString *S1, *S2;
   float **pf1, **pf2;

   /* fold at 30C instead of the default 37C */
   temperature = 30.;      /* must be set *before* initializing  */
   /* allocate memory for fold(), could be skipped */
   initialize_fold(strlen(seq1));

   /* allocate memory for structure and fold */
   struct1 = (char* ) space(sizeof(char)*(strlen(seq1)+1));
   e1 =  fold(seq1, struct1);

   struct2 = (char* ) space(sizeof(char)*(strlen(seq2)+1));
   e2 =  fold(seq2, struct2);

   free_arrays();     /* free arrays used in fold() */

   /* produce tree and string representations for comparison */
   xstruc = expand_Full(struct1);
   T1 = make_tree(xstruc);
   S1 = Make_swString(xstruc);
   free(xstruc);

   xstruc = expand_Full(struct2);
   T2 = make_tree(xstruc);
   S2 = Make_swString(xstruc);
   free(xstruc);
```

```
    /* calculate tree edit distance and aligned structures with gaps */
    edit_backtrack = 1;
    tree_dist = tree_edit_distance(T1, T2);
    free_tree(T1); free_tree(T2);
    unexpand_aligned_F(aligned_line);
    printf("%s\n%s  %3.2f\n", aligned_line[0], aligned_line[1], tree_dist);

    /* same thing using string edit (alignment) distance */
    string_dist = string_edit_distance(S1, S2);
    free(S1); free(S2);
    printf("%s  mfe=%5.2f\n%s  mfe=%5.2f  dist=%3.2f\n",
         aligned_line[0], e1, aligned_line[1], e2, string_dist);

    /* for longer sequences one should also set a scaling factor for
       partition function folding, e.g: */
    kT = (temperature+273.15)*1.98717/1000.;  /* kT in kcal/mol */
    pf_scale = exp(-e1/kT/strlen(seq1));
    init_pf_fold(strlen(seq1));

    /* calculate partition function and base pair probabilities */
    e1 = pf_fold(seq1, struct1);
    pf1 = Make_bp_profile(strlen(seq1));

    e2 = pf_fold(seq2, struct2);
    pf2 = Make_bp_profile(strlen(seq2));

    free_pf_arrays();  /* free space allocated for pf_fold() */

    profile_dist = profile_edit_distance(pf1, pf2);
    printf("%s  free energy=%5.2f\n%s  free energy=%5.2f  dist=%3.2f\n",
         aligned_line[0], e1, aligned_line[1], e2, profile_dist);

    free_profile(pf1); free_profile(pf2);
}
```

In a typical Unix environment you would compile this program using: `cc -c example.c -Ihpath` and link using `cc -o example -Llpath -lRNA -lm` where *hpath* and *lpath* point to the location of the header files and library, respectively.

6 References

- D.H. Mathews, J. Sabina, M. Zucker and H. Turner (1999)
  Expanded sequence dependence of thermodynamic parameters provides robust prediction of RNA secondary structure, JMB, 288: 911-940
- M. Zuker and P. Stiegler (1981)
  Optimal computer folding of large RNA sequences using thermodynamic and auxiliary information, Nucl Acid Res 9: 133-148
- J.S. McCaskill (1990)
  The equilibrium partition function and base pair binding probabilities for RNA secondary structures, Biopolymers 29: 1105-1119
- D.H. Turner, N. Sugimoto and S.M. Freier (1988)
  RNA structure prediction, Ann Rev Biophys Biophys Chem 17: 167-192
- J.A. Jaeger, D.H. Turner and M. Zuker (1989)
  Improved predictions of secondary structures for RNA, Proc. Natl. Acad. Sci. 86: 7706-7710
- L. He, R. Kierzek, J. SantaLucia, A.E. Walter and D.H. Turner (1991)
  Nearest-Neighbor Parameters For GU Mismatches, Biochemistry 30: 11124-11132
- A.E. Peritz, R. Kierzek, N, Sugimoto, D.H. Turner (1991)
  Thermodynamic Study of Internal Loops in Oligoribonucleotides ... , Biochemistry 30: 6428–6435
- A. Walter, D. Turner, J. Kim, M. Lyttle, P. Müller, D. Mathews and M. Zuker (1994)
  Coaxial stacking of helices enhances binding of Oligoribonucleotides.., Proc. Natl. Acad. Sci. 91: 9218-9222
- B.A. Shapiro, (1988)
  An algorithm for comparing multiple RNA secondary structures, CABIOS 4, 381-393
- B.A. Shapiro and K. Zhang (1990)
  Comparing multiple RNA secondary structures using tree comparison, CABIOS 6, 309-318
- R. Bruccoleri and G. Heinrich (1988)
  An improved algorithm for nucleic acid secondary structure display, CABIOS 4, 167-173
- W. Fontana , D.A.M. Konings, P.F. Stadler, P. Schuster (1993)
  Statistics of RNA secondary structures, Biopolymers 33, 1389-1404
- W. Fontana, P.F. Stadler, E.G. Bornberg-Bauer, T. Griesmacher, I.L. Hofacker, M. Tacker, P. Tarazona, E.D. Weinberger, P. Schuster (1993)
  RNA folding and combinatory landscapes, Phys. Rev. E 47: 2083-2099
- I.L. Hofacker, W. Fontana, P.F. Stadler, S. Bonhoeffer, M. Tacker, P. Schuster (1994) Fast Folding and Comparison of RNA Secondary Structures. Monatshefte f. Chemie 125: 167-188
- I.L. Hofacker (1994) The Rules of the Evolutionary Game for RNA: A Statistical Characterization of the Sequence to Structure Mapping in RNA. PhD Thesis, University of Vienna.
- D. Adams (1979)
  The hitchhiker's guide to the galaxy, Pan Books, London

Function Index

A

add_root 9

B

b2C 9
b2HIT 9
b2Shapiro 9
bp_distance 10

E

energy_of_struct 2
expand_Full 9
expand_Shapiro 9

F

fold 2
free_arrays 2
free_pf_arrays 3
free_profile 12
free_tree 11

G

get_line 14
gmlRNA 13

H

hamming 13

I

init_pf_fold 3
initialize_fold 2
int_urn 14
inverse_fold 3
inverse_pf_fold 3

M

Make_bp_profile 12
make_pair_table 13
Make_swString 11
make_tree 11

N

nrerror 14

P

pack_structure 13
parse_structure 9
pf_fold 2
profile_edit_distance 12
PS_dot_plot 13
PS_rna_plot 13

R

random_string 13
read_parameter_file 5

S

space 14
string_edit_distance 11

T

time_stamp 13
tree_edit_distance 11

U

unexpand_aligned_F 9
unexpand_Full 9
unpack_structure 13
unweight 9
update_fold_params 2
update_pf_params 3
urn 14

W

write_parameter_file 5

Variable Index

*

*symbolset 3

A

aligned_line[2] 11

B

backtrack_type 5
base_pair 4

C

cost_matrix 11

D

dangles 4
do_backtrack 5

E

edit_backtrack 11
energy_set 4

F

fold_constrained 5

G

give_up 3

H

helix_size[] 10

I

iindx 5

L

loop_degree[] 9
loop_size[] 9
loops 10

N

no_closingGU 4
noGU 4
noLonelyPairs 4
nonstandards 4

P

pairs 10
pf_scale 5
pr 5

R

rna_plot_type 13

T

temperature 4
tetra_loop 4

U

unpaired 10

X

xsubi[3] 14

Table of Contents

1 Introduction 1

2 Folding Routines 2
- 2.1 Minimum free Energy Folding 2
- 2.2 Partition Function Folding 2
- 2.3 Inverse Folding 3
- 2.4 Global Variables for the Folding Routines 4
- 2.5 Energy Parameter Files 5

3 Parsing and Comparing of Structures 8
- 3.1 Representations of Secondary Structures 8
- 3.2 Parsing and Coarse Graining of Structures 9
- 3.3 Distance Measures 10
  - 3.3.1 Functions for Tree Edit Distances 11
  - 3.3.2 Functions for String Alignment 11
  - 3.3.3 Functions for Comparison of Base Pair Probabilities .. 12

4 Utilities 13

5 A Small Example Program 15

6 References 17

Function Index 18

Variable Index 19
